# Secreted Autotransporter Toxin (Sat) Mediates Innate Immune System Evasion

**DOI:** 10.3389/fimmu.2022.844878

**Published:** 2022-02-17

**Authors:** Claudia A. Freire, Rosa M. Silva, Rita C. Ruiz, Daniel C. Pimenta, Jack A. Bryant, Ian R. Henderson, Angela S. Barbosa, Waldir P. Elias

**Affiliations:** ^1^ Laboratório de Bacteriologia, Instituto Butantan, São Paulo, Brazil; ^2^ Institute of Microbiology and Infection, University of Birmingham, Birmingham, United Kingdom; ^3^ Departamento de Microbiologia, Imunologia e Parasitologia, Escola Paulista de Medicina, Universidade Federal de São Paulo, São Paulo, Brazil; ^4^ Laboratório de Bioquímica, Instituto Butantan, São Paulo, Brazil; ^5^ Institute for Molecular Bioscience, University of Queensland, Brisbane, QLD, Australia

**Keywords:** SPATE, extraintestinal *Escherichia coli*, complement system, immune evasion, Sat, sepsis

## Abstract

Several strategies are used by *Escherichia coli* to evade the host innate immune system in the blood, such as the cleavage of complement system proteins by secreted proteases. Members of the Serine Proteases Autotransporters of Enterobacteriaceae (SPATE) family have been described as presenting proteolytic effects against complement proteins. Among the SPATE-encoding genes *sat* (secreted autotransporter toxin) has been detected in high frequencies among strains of *E. coli* isolated from bacteremia. Sat has been characterized for its cytotoxic action, but the possible immunomodulatory effects of Sat have not been investigated. Therefore, this study aimed to evaluate the proteolytic effects of Sat on complement proteins and the role in pathogenesis of BSI caused by extraintestinal *E. coli* (ExPEC). *E. coli* EC071 was selected as a Sat-producing ExPEC strain. Whole-genome sequencing showed that *sat* sequences of EC071 and uropathogenic *E. coli* CFT073 present 99% identity. EC071 was shown to be resistant to the bactericidal activity of normal human serum (NHS). Purified native Sat was used in proteolytic assays with proteins of the complement system and, except for C1q, all tested substrates were cleaved by Sat in a dose and time-dependent manner. Moreover, *E. coli* DH5α survived in NHS pre-incubated with Sat. EC071-derivative strains harboring *sat* knockout and in trans complementations producing either active or non-active Sat were tested in a murine sepsis model. Lethality was reduced by 50% when mice were inoculated with the *sat* mutant strain. The complemented strain producing active Sat partially restored the effect caused by the wild-type strain. The results presented in this study show that Sat presents immunomodulatory effects by cleaving several proteins of the three complement system pathways. Therefore, Sat plays an important role in the establishment of bloodstream infections and sepsis.

## Introduction

Bloodstream infections (BSI) result from the presence of viable bacteria or other microorganisms in the blood that trigger an inflammatory response in the host (1). After gaining access to the bloodstream, bacteria face the host innate immune system and the pathogen is efficiently eliminated. However, in some cases the pathogen can prevail, and the host response becomes unbalanced and harmful, leading to the development of sepsis (2). The clinical outcome of a BSI is influenced by several factors, such as the agent causing the infection and its arsenal of virulence traits, the frequency of bloodstream invasion by the pathogen, availability of diagnostic resources, host response to the infection and the delivery of appropriate early treatment (3).

Extraintestinal *Escherichia coli* (ExPEC) is among the most frequent pathogens causing BSI whose primary sources of infection can be the intestines or intravascular medical devices or be secondary to other extraintestinal infections (4–8). To survive in the bloodstream, *E. coli* must resist the bactericidal activity of the complement system. The complement system consists of a regulated network of proteins in the serum or associated with cell membranes involved in host defense and inflammation (9, 10). Pathogens may employ several mechanisms to evade the complement system, by targeting different steps of activation and/or regulation of this cascade. The secretion of proteases that directly degrade complement proteins is an efficient strategy to avoid complement activation (6, 11, 12). Two members of the Serine Protease Autotransporters of Enterobacteriaceae (SPATE) family, EspP and Pic, are involved in the cleavage of complement molecules and together with other members of this family play an important role in *E. coli* pathogenesis (13–16).

SPATE is a superfamily of secreted virulence factors highly prevalent in enteropathogens, including *E. coli* and *Shigella*. These proteases are responsible for the degradation of intra or extracellular substrates (14, 17, 18) and their structure is remarkably similar, composed of three domains: an N-terminal signal peptide, a passenger domain, and a C-terminal translocator domain. The passenger domain, which is entirely secreted to the extracellular milieu, constitutes the mature form of the SPATE proteins and is responsible for their biological activity (14, 19, 20).

Amino acid sequence analysis of the passenger domains classified SPATEs in two different groups: class 1, comprising SPATEs with cytotoxic effects, and class 2, comprising immunomodulatory SPATEs (14, 19). Although these specific biological functions were observed among the members of the same class, studies have shown that both biological activities can be displayed by some SPATEs. EspP, a class 1-SPATE, cleaves complement proteins, indicating that this SPATE can also exert an immunomodulatory activity (13). Moreover, the class-2 SPATE SepA was shown to be involved in barrier disruption, facilitating bacterial translocation and epithelium invasion (21).

SPATE-encoding genes are present in mobile elements, such as plasmids, pro-phages and pathogenicity islands, allowing their dissemination among different *E. coli* lineages. In a previous study, our group characterized a collection of *E. coli* isolated from human bacteremia in terms of frequency of SPATE-encoding genes, phylogeny and genetic markers for intrinsic virulence (22). *sat* was the most frequent gene (34.2%), similarly to the frequency observed by others, with *sat* frequencies ranging between 25 to 70% (22–32). Further to this, *sat* is among the most frequent SPATE-encoding genes found in uropathogenic *E. coli* (UPEC) (33–37).

The Secreted autotransporter toxin (Sat) is a 107-kDa protein firstly described in the prototype UPEC strain CFT073, encoded by a 3.9-kb gene located in the pathogenicity island II (38). Sat is a class 1-SPATE and its cytotoxic effects on urinary tract cells (Vero, HK-2, CRL-1749, CRL-1573 and HEK-293) are well characterized, showing its role in the pathogenesis of UTI caused by UPEC (38–40). This SPATE also displays enterotoxic activity (41), promotes reorganization of tight-junction associated proteins in Caco-2/TC7 cells, and increases cellular permeability (42) and cellular detachment in HeLa cells (43). Recently, it was reported that Sat causes intense cytotoxic effects on human umbilical vein endothelial (HUVEC) cells, indicating a possible role in the pathogenesis of BSI and sepsis (44). Further functional characterizations have shown that Sat has a proteolytic activity on spectrin and coagulation factor V (45). However, no other studies assessing possible immunomodulatory effects or extracellular substrates of Sat have been reported so far.

The high frequency of *sat* in *E. coli* strains isolated from bacteremia and Sat cytotoxic effects on endothelial and urinary tract cells suggest that this SPATE may be involved in different steps of BSI and sepsis pathogenesis. Considering that the complement system is the first barrier of the host innate immune response faced by *E. coli* in the bloodstream, we hypothesized that Sat could contribute to bacterial immune evasion by inactivating complement molecules. In the present work, we have assayed Sat proteolytic activity over complement proteins and evaluated its role in the pathogenesis of sepsis.

## Material and Methods

### Bacterial Strains and Growth Conditions

The *E. coli* strain EC071 was isolated from the blood of a patient with bacteremia and verified for the presence of SPATE-encoding genes, intrinsic virulence genetic markers and phylogroup classification (22). EC071 harbors no SPATE-encoding genes but *sat* and is classified as an ExPEC+ strain, according to the criteria determined by Johnson et al. (46). It belongs to phylogroup F, according to the revisited Clermont method (47). Further bacterial strains used in this work are described in the **Supplementary Table 1**.

All strains were routinely grown in Luria-Bertani (LB) broth at 37°C supplemented with ampicillin (100 µg/ml), kanamycin (150 µg/ml), or tetracycline (15 µg/mL), when indicated. Bacterial stocks were kept on LB supplemented with glycerol 20% (vol/vol) at –80°C.

### Detection of Sat Production by *E. coli* EC071

*E. coli* EC071 was grown for 18 h in 5 mL of LB broth at 37°C under constant shaking (250 rpm). The culture was harvested at 2.000 x g for 15 min at 4°C and 1 mL aliquots of the supernatant were precipitated with 10% trichloroacetic acid (TCA) (Sigma-Aldrich, USA), as described elsewhere (48). Culture supernatants of enteroaggregative *E. coli* (EAEC) EC233/93 and diffusely-adherent *E. coli* (DAEC) FBC 114 were prepared as described above and used as Sat-producing strains (positive controls). *Shigella flexneri* M90T culture supernatant, similarly prepared, was used as a negative control (41, 44, 49).

The resulting precipitated supernatants were denatured with β-mercaptoethanol at 96°C for 5 min for further analysis by 10% SDS-PAGE (2 independent gels) (50). The first gel was stained by silver nitrate (51) and the second one was used for immunoblotting assays, employing polyclonal anti-Sat serum (44) and peroxidase-conjugated anti-rabbit IgG as secondary antibody (Sigma-Aldrich). Signal detection was performed using SuperSignal^®^ West Pico Enhanced Chemiluminescent Substrate (ThermoFisher Scientific) and the Alliance Image System (UVITEC, UK).

### *E. coli* EC071 Whole Genome Sequencing and Plasmid Analysis

EC071 genomic DNA was extracted from an overnight culture in LB broth at 37°C using the GeneJet Genomic DNA Purification Kit (ThermoFisher Scientific, USA), according to the manufacturer´s instructions. Following extraction, DNA was analyzed by electrophoresis in a 1% agarose gel and quantified using the Qubit™dsDNA HS Assay Kit (Invitrogen, USA), according to the manufacturer’s instructions. EC071 genomic DNA was submitted to the MicrobesNG (University of Birmingham, UK) facility where DNA libraries were prepared using Nextera XT Library Prep Kit (Illumina, USA) and sequenced by the Illumina HiSeq 2500 platform, using a 250 bp paired-end protocol. Reads were adapter trimmed using Trimmomatic 0.30 with a sliding window quality cutoff of Q15 (52). *De novo* assembly and annotation of the genome were performed using SPAdes version 3.7 (53) and Prokka 1.11 (54), respectively. The genome assembly metric was calculated using QUAST (55).

After sequencing, EC071 whole genome was analyzed for the presence/absence of SPATE-encoding genes, intrinsic virulence gene markers, and bactericidal serum activity resistance-related genes by alignment with the respective genetic sequences available in the ecoli VF collection database (https://github.com/aleimba/ecoli_VF_collection) using BLAST (https://ncbi.nlm.nih.gov/genbank/). The phylogenetic classification was accessed by the ClermontTyping online tool (http://clermontyping.iame-research.center/) (56), while serotype, sequence type (ST) and presence of plasmids were verified by the SerotypeFinder 2.0, MLST 2.0 and PlasmidFinder 2.0, respectively, at the Center for Genomic Epidemiology webpage (http://www.genomicepidemiology.org/services/). *sat* nucleotide sequence of EC071 and its predicted amino acid sequence were also compared to the corresponding sequences of prototype UPEC CFT073 (accession numbers AF289092.1 and AAG30168.1).

The plasmid profile of *E. coli* EC071 was determined by alkaline lysis (57) followed by agarose gel (0.8%) electrophoresis analysis.

### Resistance of *E. coli* EC071 to the Bactericidal Activity of Human Serum

Resistance of *E. coli* EC071 to the bactericidal activity of normal human serum (NHS) was assessed as previously described (30, 58). Briefly, EC071 was grown in 50 mL of LB broth and incubated at 37°C under constant shaking (250 rpm) until the optical density at 600 nm (OD600) of 0.5 was reached. *E. coli* DH5α, a negative control, was submitted to the same protocol.

Simultaneously, 100 µL of NHS (Sigma-Aldrich, USA) were added to 80 µL of sterile 0.01 M PBS in duplicates for each strain. The first tube was incubated at 37°C for 30 min before the test. Heat inactivated human serum (heat-IHS) was obtained by incubating the second tube at 56°C for 30 min. A third tube containing 180 µL of sterile 0.01M PBS (viability control) was also incubated at 37°C for 30 min. Then, 20 µL of the bacterial inoculum were added to each tube. NHS and heat-IHS tubes were then incubated at 37°C and 20 µL of each were collected after 30 and 60 min of incubation. The collected volume of each time point, as well as the viability control tube, were serial diluted and plated onto MacConkey agar plates. The plates were incubated at 37°C for 18 h for colony-forming unity (CFU)/mL enumeration. Results obtained for each tested condition at each period of incubation were compared using ANOVA and Tukey’s multiple comparison tests, using a 95% confidence interval

### Purification of Sat From *E. coli* EC071

Sat purification was carried as described by Maroncle et al. (40) with slight modifications. Initially, *E. coli* EC071 was grown statically in 20 mL of LB broth at 37°C for 18 h and then subcultured to 1 L of LB broth at 37°C under constant shaking (250 rpm), until the optical density at 600 nm (OD600) of 1.0 was reached. The large-scale culture was centrifuged at 8.000 x g for 15 min at 4°C and the supernatant was vacuum filtered in a 0.22 µm membrane (Millipore, USA). The filtered supernatant was first 100-fold concentrated in a 30-kDa cutoff centrifugal device (Millipore, USA) in successive centrifugations at 5.000 x g for 20 min at 4°C. The crude concentrate was then 10-fold concentrated in a 50-kDa centrifugal device (Millipore, USA) in single centrifugation at 5.000 x g for 20 min at 4°C. The refined concentrate was diluted in an anion exchange buffer (0.025 M NaCl, 0.025 M Tris-HCl, pH 7.5) to 15 mL and then submitted to a Q Sepharose Fast Flow column for anionic exchange (GE Healthcare, USA), previously washed with 5 column-volumes of anion exchange buffer. Elution was carried with 15 mL of 0.025 M Tris-HCl buffers pH 7.5 containing different concentrations of NaCl (0.025 M, 0.100 M, 0.175 M, 0.250 M, 0.325 M, 0.400 M and 0.500 M) at a flow rate of 2 mL/min. Fractions collected during the elution were analyzed by SDS-PAGE, silver nitrate staining and immunoblotting as described above for Sat detection in culture supernatants. Fractions containing Sat were quantified using the Pierce™ BCA Protein Assay Kit (ThermoFisher Scientific), as instructed by the manufacturer.

The identity of Sat in each of these fractions was confirmed by mass spectrometry analysis, through a gel-based proteomics approach. The in-gel digestion was conducted as described elsewhere (59) with small modifications. Firstly, the gel bands were selected, excised, and transferred to a 1.5-mL microtube. Subsequently, a solution of 75 mM ammonium bicarbonate (in 40% ethanol) was added to destain the bands. Thereafter, the supernatant was removed, 5 mM dithiothreitol (in 25 mM ammonium bicarbonate) was added, and all samples were incubated at 60°C for 30 min (reduction step); next, we added 55 mM iodoacetamide (in 25 mM ammonium bicarbonate) and incubated all samples at room temperature for 30 min in the absence of light. The supernatant of all individual samples was removed, and the gel pieces were dehydrated by adding acetonitrile (ACN). Subsequently, 10 µL of proteomic grade trypsin solution (10 ng/µL in 50 mM ammonium bicarbonate) was added to each sample, and digestion was allowed for 45 min on ice. Thereafter, supernatants were removed, gel pieces were covered with 50 mM ammonium bicarbonate and incubated overnight at 30°C. Finally, each sample was suspended in 20 µL of ACN/5% trifluoroacetic acid (TFA) (1:1, v/v) and sonicated for 10 min. The supernatant was removed and dispensed in a separate tube. We repeated this step three times and combined the supernatants of the same samples. Lastly, we repeated the process using ACN instead of ACN/5% TFA. The obtained supernatant was combined with the previously obtained supernatants.

The tryptic peptides were analyzed by liquid chromatography-mass spectrometry (LC–MS) using an electrospray-ion trap-time of flight (ESI-IT-TOF) system coupled to a binary ultra-fast liquid chromatography system (UFLC) (20A Prominence, ShimadzuKyoto, Japan). Briefly, samples were dried, resuspended in 0.1% acetic acid, and loaded onto a C18 column (Discovery C18, 5 μm, 50 × 2.1 mm) operating with a binary solvent system: (A) water:acetic acid (999:1, v/v) and (B) ACN:water:acetic acid (900:99:1, v/v/v). The column was eluted at a constant flow rate of 0.2 mL/min with a 0 to 40% linear gradient of solvent B for 40 min. The eluates were monitored by a Shimadzu SPD-M20A PDA detector before introduction into the mass spectrometer. The interface voltage was set to 4.5 kV, the capillary voltage used was 1.8 kV at 200°C, and the fragmentation was induced by argon collision at 50% ‘energy’. The MS spectra were acquired under the positive mode and collected in the range of 350 to 1400 m/z. The MS/MS spectra were collected in the range of 50 to 1950 m/z.

Raw LCD LCMSolution Shimadzu data were converted into MGF by the LCMSolution tool and then loaded into Peaks Studio V7.0 (BSI, Canada). Data were processed according to the following parameters: MS and MS/MS error mass were 0.1 Da; methionine oxidation and carbamidomethylation as variable and fixed modification, respectively; trypsin as cleaving enzyme; maximum missed cleavages (3), maximum variable PTMs per peptide (3) and non-specific cleavage (both). Data were analyzed against the whole UniProt protein database.

### Proteolysis of Complement System Proteins

Initially, the proteolytic activity of purified Sat was tested against the following purified complement proteins (Complement Technology, USA): C1q, C2, C3 and C3b, C4 and C4b, C5, C6, C7, C8 and C9. To identify possible Sat substrates among these complement proteins, 5 µg of Sat were incubated with 0.5-1.0 µg of each complement molecule in the presence of MOPS buffer (0,1 M MOPS, 0,2 M NaCl and 0,01 mM ZnSO4, pH 7,3) (40) at 37°C for 5 or 24 h. As a control for spontaneous cleavage, complement molecules diluted in MOPS buffer were incubated under the same conditions. Incubation products were analyzed by immunoblotting using specific antibodies to each complement protein (Complement Technology, USA), and peroxidase-conjugated anti-goat IgG as the secondary antibody (Sigma-Aldrich). Signal detection was performed using the SuperSignal^®^ West Pico Enhanced Chemiluminescent Substrate (ThermoFisher Scientific) and the Alliance Image System (UVITEC, UK).

Dose dependency of Sat-induced cleavage of the substrates was evaluated using lower concentration of purified Sat (0.5 or 1.0 µg). Also, inhibition of Sat proteolytic activity was assessed by incubating purified Sat (0.5 or 1.0 µg) with 1.0 mM phenylmethylsulfonyl fluoride (PMSF) for 30 min at room temperature before the addition of complement proteins. Incubation products were analyzed as described above.

### *E. coli* DH5α Resistance in Sat-Treated Human Serum

The capacity of *E. coli* DH5α to survive in Sat pre-treated NHS (Sat-NHS) was assessed. Considering that 0.5 µg of Sat cleaved 0.5 µg of C4 and the C4 concentration in NHS (0.6 µg/µL), 56 µL of one fraction of purified Sat (0.270 µg/µL), resulting in a total of 15 µg of purified Sat, were added to 25 µL of NHS (15 µg of C4) and enough volume of sterile 0.01 M PBS to complete 100 µL (serum final concentration: 25%). Reactions were incubated at 37°C for 5 h and then 10 µL of *E. coli* DH5α inoculum (OD600 0.5) were added to each tube, including the controls NHS without Sat, heat-IHS and 0.01 M PBS without serum. The tubes were then incubated at 37°C, and after 30 and 60 min 20 µL of each incubation were collected, serial diluted and plated onto MacConkey agar plates for CFU/mL enumeration, after incubation at 37°C for 18 h. Results obtained for each tested condition at each period of incubation were compared using ANOVA and Tukey’s multiple comparison tests, using a 95% confidence interval.

### *E. coli* EC071-Based Genetic Constructions

Mutagenesis of *sat* in *E. coli* EC071 was achieved by homologous recombination using the suicide vector pJP5003 (60). Briefly, a 930-bp fragment of *sat* was amplified by PCR with *sat* primers (**Supplementary Table 2**) and genomic DNA of EC071 as template, prepared using the GeneJet Genomic DNA Purification Kit (ThermoFisher Scientific, USA), according to the manufacturer’s instructions. PCR cycling was conducted as follows: 94°C/5 min (1 cycle); 94°C/1 min; 59°C/1 min; 72°C/1 min (30 cycles); 72°C/5 min (1 cycle). Amplification products were analyzed by electrophoresis in a 0.7% agarose gel in Tris borate-EDTA (TBE) buffer (0.5X). After gel analysis, the corresponding product was purified using the Monarch^®^ PCR & DNA Cleanup Kit (New England Biolabs, USA).

The amplified fragment was cloned in pGEM-T Easy (Promega, USA), according to the manufacturer’s instructions. Ligation products were transformed in chemically competent *E. coli* DH5α (61) and transformants harboring the insert were selected on LB agar containing ampicillin (100 µg/mL). One selected transformant was named DH5α(pCF1). pJP5603 and pCF1 were purified with Pure Yield™ Plasmid Miniprep System Kit (Promega) and digested with EcoRI (Invitrogen, USA). Digestion products were analyzed in a 0.7% agarose gel and the insert released from pCF1 was gel extracted with Monarch^®^ DNA Gel Extraction Kit (New England Biolabs), while pJP5603 was purified with Monarch^®^ PCR & DNA Cleanup Kit (New England Biolabs). Insert and pJP5603 were submitted to ligation with T4 DNA ligase (Invitrogen, USA) and transformed in S17-λpir chemically competent cells (61). Transformants were selected on LB agar containing kanamycin (150 µg/mL). One selected transformant was named S17-λpir (pCF2). S17-λpir (pCF2) and EC071 were submitted to conjugation, as previously described (62). One transconjugant was selected on MacConkey agar containing kanamycin (150 µg/mL) and tetracycline (15 µg/mL) and named EC071::pCF2. The correct insertion of pCF2 in the genome of EC071 was checked by PCR with different combinations of *sat* and M13 primers (**Supplementary Table 2**). PCR cycling was conducted as follows: 94°C/5 min (1 cycle); 94°C/1 min; 59°C/1 min; 72°C/1 min (30 cycles); 72°C/5 min (1 cycle). Amplification products were analyzed by electrophoresis in a 0.7% agarose gel in TBE buffer (0.5X). After gel analysis, the corresponding product was excised from the agarose gel and purified using the Monarch^®^ PCR & DNA Cleanup Kit (New England Biolabs, USA) and Sanger sequenced with their respective primers. The absence of Sat production in EC071::pCF2 was also confirmed by immunoblotting as described above for Sat production by EC071.

A *sat* minimal clone was also obtained for complementation purposes. Primers *sat NdeI*(F)/*sat XhoI*(R) and *NdeI pettac*(F)/*XhoI pettac*(R) were designed (**Supplementary Table 2**) for amplification of the complete sequence of *sat* and the vector *pettac* (63), respectively. The 3.9 kb and 5.3 kb fragments, corresponding to *sat* and *pettac*, respectively, were amplified using Phusion^®^ High-fidelity DNA polymerase (New England Biolabs). PCR cycling was conducted as follows: 98°C/2 min (1 cycle); 98°C/30 sec; 60°C/30 sec; 72°C/5 min (30 cycles); 72°C/5 min (1 cycle). Amplification products were analyzed by electrophoresis in a 0.7% agarose gel in TBE buffer (0.5X). The insert and the vector were then purified using QIAquick^®^ PCR Purification Kit (Qiagem, Germany) and digested with NdeI and XhoI at 37°C for 3 h. During the final hour of incubation, the vector was also dephosphorylated by CIP (New England Biolabs). Digestion products were purified using QIAquick^®^ PCR Purification Kit (Qiagem) and submitted to ligation with T4 DNA ligase (New England Biolabs), according to the manufacturer’s instructions. Ligation products were transformed in chemically competent *E. coli* DH5α cells (61). One transformant was selected on LB agar containing ampicillin (100 µg/mL) and named DH5α(pCF3). Plasmid pCF3 was extracted using the QIAprep^®^ Spin Miniprep Kit (Qiagen), and correct cloning was confirmed by Sanger sequencing of the 5’ and 3’ ends of the insert using primers *pettac* (F) and *T7 terminator* (R) (**Supplementary Table 2**), respectively. pCF3 was also transformed in chemically competent *E. coli* MG1655 cells (61). Sat expression by DH5α(pCF3) and MG1655(pCF3) was confirmed by immunoblotting as described above for Sat production by EC071.

Site-directed mutagenesis was performed to inactivate the serine protease active site of Sat, by exchanging the serine residues in positions 256 and 258 for an isoleucine and an alanine, respectively (S256I/S258A) (40). Primers *sat sdm* were designed (**Supplementary Table 2**) based on the sequence of the active site with two nucleotide changes corresponding to the mentioned amino acid changes and annealing at the same site from both DNA strands, to complete amplify pCF3 containing the exchanges in the amplification products. Amplifications were performed using pCF3 as template and the Velocity DNA Polymerase (Bioline, USA). PCR cycling was conducted as follows: 98°C/30 sec (1 cycle); 98°C/30 sec; 60°C/30 sec; 72°C/5 min 30 sec (20 cycles); 72°C/1 min (1 cycle). Amplified products were treated with DpnI at 37°C for 1 h (New England Biolabs), for further transformation in chemically competent *E. coli* DH5α cells (61). One transformant was selected on LB agar containing ampicillin (100 µg/mL) and named pCF4. Plasmid pCF4 was extracted using the QIAprep^®^ Spin Miniprep Kit (Qiagem) and Sanger sequenced with *sat2* primers (**Supplementary Table 2**) to confirm the mutation. pCF4 was also transformed in chemically competent *E. coli* MG1655 cells (61). Sat production by DH5α(pCF4) and MG1655(pCF4) was confirmed by immunoblotting as described above.

Sat mutation in EC071 (EC071::pCF2) was complemented in trans. Chemically competent EC071::pCF2 cells were prepared (61) and transformed with pCF3 and pCF4. Transformants were selected on LB agar containing ampicillin (100 µg/mL) and kanamycin (150 µg/mL) and named as EC071::pCF2 (pCF3) and EC071::pCF2 (pCF4). Sat production by these strains was confirmed by immunoblotting as described above.

### Growth Curve of CFT073, MG1655, EC071 and Derived Strains

Each strain was grown statically in 3 mL of LB broth or LB broth with the appropriate antibiotic at 37°C for 18 h. Then, 500 µL of each culture were transferred to 50 mL of LB broth, LB broth with kanamycin (150 µg/mL) and/or ampicillin (100 µg/mL) and incubated at 37°C under constant shaking (250 rpm). Bacterial growth was monitored by OD600 readings every 30 min for 6 h. Biological replicates were performed for each strain. Also, aliquots correspondent to each time point were serial diluted and plated onto MacConkey agar plates containing the appropriate antibiotic to determine the correspondence between the absorbance and CFU/mL at each time point.

### Murine Sepsis Model

The sepsis murine model was employed to assess the lethality of *E. coli* EC071 and its derived strains following the protocol described by Picard et al. (64). This experimentation was approved by the Ethics Committee on Animal Use of the Butantan Institute (CEUAIB protocol number 5743060220).

*E. coli* EC071, its derivative strains and *E. coli* MG1655(pCF3) harboring *sat* minimal clone were tested in this model. UPEC CFT073 and *E. coli* MG1655 were used as positive and negative controls, respectively. Strains were grown in 50 mL of LB broth containing the appropriate antibiotics at 37°C under constant shaking (250 rpm), until the optical density at 600 nm (OD600) corresponding to 10^9^ CFU/mL was reached. Then, 1 mL of each culture was harvested at 2.500 x g for 10 min and the pellet was washed twice with sterile saline solution (0.9%). Finally, the pellet was resuspended in 1 mL of saline solution.

Female Swiss mice aged between six and eight weeks and weighing between 20-30 g were used. Each of the following strains was inoculated in ten mice: EC071, EC071::pCF2, EC071::pCF2(pCF3) and EC071::pCF2(pCF4); while strain MG1655(pCF3) was inoculated in eight mice and strains UPEC CFT073 and *E. coli* MG1655 in four mice each. Each animal was inoculated subcutaneously with 200 µL (2 x 10^8^ CFU) of the bacterial suspension and observed at each hour during six hours after the inoculum, at the eighteenth hour and daily up to seven days post-infection or until the animal naturally died or reached the endpoint determined by the presentation of clinical signs such as body weight loss, fur aspect, posture and motility alteration and dehydration (65). The animals that reached the endpoint during the experiment course or survived during seven days of observation were euthanized by an anesthetics overdose of xylazine (30 mg/kg) and ketamine (300 mg/kg). Lethality rates were compared between each experimental group using Fisher’s exact test, with a 95% confidence interval.

## Results

### *E. coli* EC071 Is a Sat-Producing ExPEC

**Figure 1:**
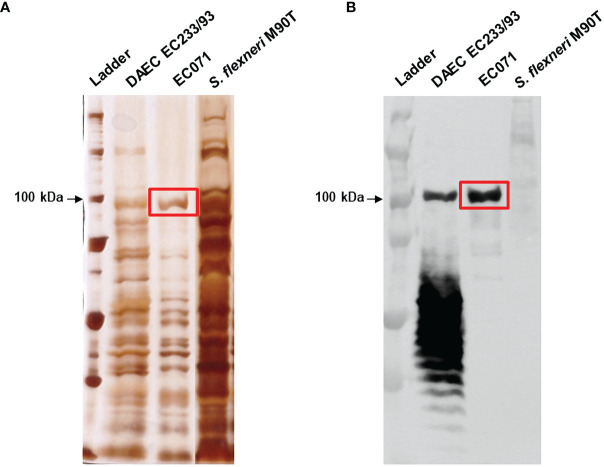
Sat production by EC071. TCA-treated LB culture supernatants of EC071, DAEC EC233/93 (positive control) and *Shigella flexneri* M90T (negative control) were analyzed by 10% SDS-PAGE. **(A)** Silver nitrate-stained gel; **(B)** Immunoblotting with anti-Sat (1:500) and peroxidase-conjugated goat anti-rabbit IgG (1:10,000). Ladder: Precision Plus Protein Kaleidoscope Prestained Protein Standard (BioRad, USA).

To assess Sat production by EC071, precipitated LB broth over-night culture supernatant was submitted to SDS-PAGE analysis followed by immunodetection using a Sat specific anti-serum (44). Silver nitrate gel staining revealed a protein band corresponding to approximately 100 kDa present in EC071 TCA-treated culture supernatant (**Figure 1A**). Immunoblotting detection showed that the corresponding protein band was recognized by anti-Sat demonstrating the production and secretion of Sat by *E. coli* EC071 (**Figure 1B**). Therefore, EC071 was employed in further experiments as the Sat-producing prototype ExPEC strain.

**Figure 2:**
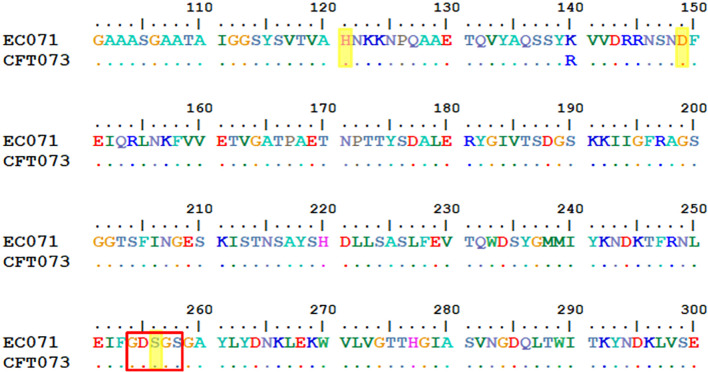
Partial alignment between Sat amino acid predicted sequences of prototype UPEC CFT073 (accession number AAG30168.1) and EC071. The catalytic triad (highlighted in yellow) composed by His, Asp and Ser (residues 121, 149 and 256, respectively) are conserved in both sequences as well as the characteristic serine protease motif GDSGS of the SPATEs family, harboring the catalytic serine (red box).

EC071 WGS was performed to analyze its genetic background concerning the presence of SPATE-encoding genes, intrinsic virulence genes, as well as genes related to resistance to the bactericidal activity of human serum. The alignment between *sat* sequences from EC071 and CFT073 (accession number AF289092.1) genomes showed 99% of identity. Also, 99% of similarity between their respective predicted amino acid sequences was verified. The catalytic triad, composed by His, Asp and Ser, is conserved in both sequences as well as the characteristic serine protease motif GDSGS of the SPATEs family, harboring the catalytic serine residue (**Figure 2**). Still, Sat predicted amino acid sequence from EC071 was aligned to multiple Sat amino acid sequences available in the National Center for Biotechnology Information (NCBI) database (https://www.ncbi.nlm.nih.gov/) and a minimum of 99.85% of identity was verified, indicating that Sat sequence is highly conserved among different *E. coli* strains (**Supplementary File 1**).

The genetic background of EC071 genome is disclosed in **Supplementary Table 3**. No other SPATE-encoding gene was detected in EC071. The presence of *pap*, *afa* and *dra* operons confirmed the classification of EC071 as EXPEC, following the criterion proposed by Johnson et al. (46). The following genes encoding serum resistance-related proteins were detected among the sequences available in the ecoli VF collection database (https://github.com/aleimba/ecoli_VF_collection): *nlpI* (accession number: CU928161.2), *prc* (accession number: NC_000913.3), *ompX* (accession number: U00096.3), *ompTc* (accession number: NC_008563.1) and *ompTp* (accession number: NC_007675.1). Phylogenetic classification, sequence type (ST), serotype and the occurrence of plasmids were evaluated using the ClermontTyping online tool (http://clermontyping.iame-research.center/) and the online tools available at the Center for Genomic Epidemiology webpage (http://www.genomicepidemiology.org/services/). EC071 was assigned to phylogroup F, serotype O1:H7 and ST 59. Plasmid analysis revealed five different origins of replication: Col(MP18), Col 156, Col8282, IncB/O/K/Z and IncX1, suggesting the presence of at least five plasmids in EC071. Plasmid extraction confirmed this result, since at least five plasmids could be clearly visualized after agarose gel electrophoresis analysis (**Supplementary Figure 1**). Still, none of these origins of replication was detected in the same contig containing *sat*.

EC071 genome sequence was deposited in GenBank under the accession numbers NZ_JAFFIA010000001.1 until NZ_JAFFIA010000240.1. Sequencing metrics are shown in **Supplementary Table 4**.

### *E. coli* EC071 Is Able to Resist the Bactericidal Activity of Normal Human Serum (NHS)

**Figure 3:**
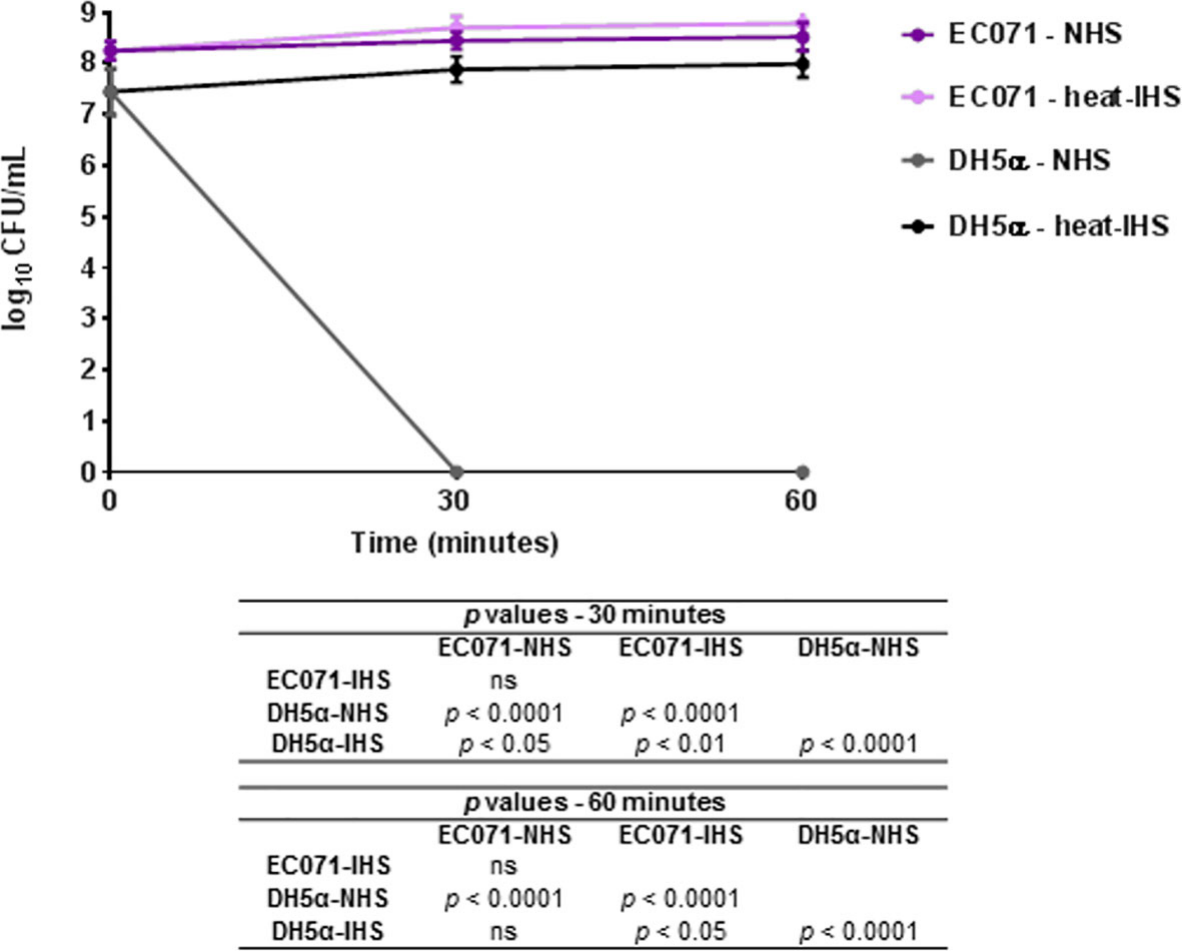
Susceptibility of *E. coli* EC071 to the bactericidal activity of human serum. EC071 and DH5α (serum-sensitive control) were incubated at 37°C for 30 and 60 min with normal human serum (NHS) or heat-inactivated human serum (heat-IHS) in final concentrations of 25%. Data are presented as CFU/mL counts on MacConkey agar plates. Time zero CFU/mL correspond to the initial inocula before contact with NHS or heat-IHS. Results obtained for each tested condition at each time point were compared using ANOVA and Tukey’s multiple comparison tests. ns, not significant.

The capacity of EC071 to resist the bactericidal activity of NHS was evaluated. EC071 and DH5α cultures were incubated in NHS or previously heat-inactivated human serum (heat-IHS), in a final concentration of 25%, and colony-forming units (CFU/mL) were counted. As shown in **Figure 3**, EC071 completely survived in NHS, since no differences in CFU/mL were observed for NHS and heat-IHS. As expected, *E. coli* DH5α was killed within the first 30 min in contact with NHS and fully survived in heat-IHS (**Figure 3**).

### Sat Was Purified as a Native Protein From EC071 Culture Supernatant

**Figure 4:**
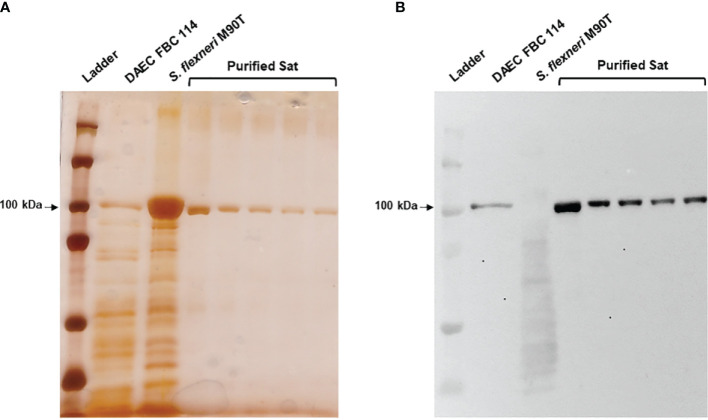
Sat purification from EC071 concentrated LB culture supernatant. Five collected elution fractions of the same batch were analyzed by 10% SDS-PAGE. TCA-treated LB culture supernatants of DAEC FBC 114 (positive control) and *S. flexneri* M90T (negative control) were used as positive and negative controls, respectively. **(A)** Silver nitrate-stained gel; **(B)** Immunoblotting with anti-Sat (1:500) and peroxidase-conjugated goat anti-rabbit IgG (1:10,000). Ladder: Precision Plus Protein Kaleidoscope Prestained Protein Standard (BioRad, USA).

EC071 culture supernatant was concentrated and submitted to an anionic exchange column for Sat purification. Elution fractions collected during this process were analyzed by SDS-PAGE and immunoblotting using anti-Sat serum (44). Silver nitrate staining revealed a single band of approximately 100 kDa in five of these fractions (**Figure 4A**), which were recognized by anti-Sat serum (**Figure 4B**). Mass spectrometry analysis of each fraction confirmed the identity of Sat (**Supplementary File 2**).

### Purified Sat Cleaves Proteins of All Three Pathways of the Complement System

**Figure 5:**
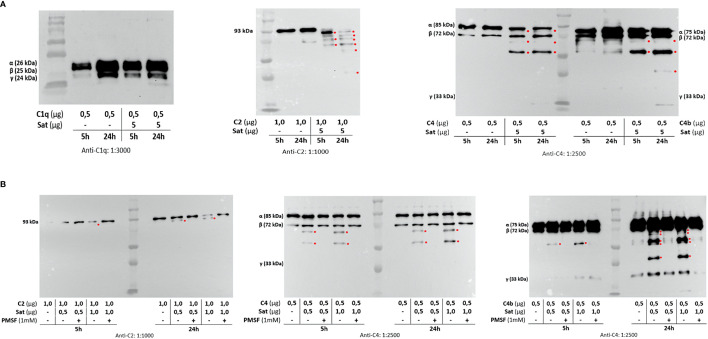
Proteolytic activity of Sat on complement proteins of the classical and the lectin activation pathways. Purified Sat and human complement proteins were incubated at 37°C for 5 and 24 h and the incubation products were analyzed by 10% SDS-PAGE, followed by immunodetection of the target proteins with specific complement antibodies (dilutions are indicated under each image) and peroxidase-conjugated anti-goat IgG (1:10,000). Reactivity was detected with the SuperSignal^®^ West Pico Enhanced Chemiluminescent Substrate kit (ThermoFisher Scientific). **(A)** Screening assays using 5 µg of Sat and 0.5 - 1.0 µg of complement proteins. **(B)** Inhibition assays using 0.5 - 1.0 µg of Sat or PMSF-inhibited Sat and 0.5 - 1.0 µg of target proteins. The molecular masses indicated in the images correspond to the chains of each protein after denaturation. Red asterisks indicate the cleavage products. Ladder: Precision Plus Protein Kaleidoscope Prestained Protein Standard (BioRad, USA).

**Figure 6:**
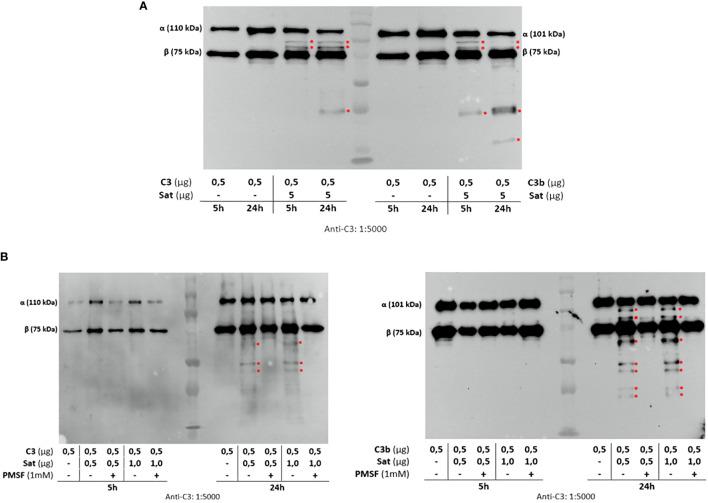
Proteolytic activity of Sat on C3 and C3b complement proteins. Purified Sat and the human complement proteins were incubated at 37°C for 5 and 24 h and the incubation products were analyzed by 10% SDS-PAGE, followed by immunodetection of the target proteins with specific complement antibodies (dilutions are indicated under each image) and peroxidase-conjugated anti-goat IgG (1:10,000). Reactivity was detected with the SuperSignal^®^ West Pico Enhanced Chemiluminescent Substrate kit (ThermoFisher Scientific). **(A)** Screening assays using 5 µg of Sat and 0.5 - 1.0 µg of complement proteins. **(B)** Inhibition assays using 0.5 - 1.0 µg of Sat or PMSF-inhibited Sat and 0.5 - 1.0 µg of target proteins. The molecular masses indicated in the images correspond to the chains of each protein after denaturation. Red asterisks indicate the cleavage products. Ladder: Precision Plus Protein Kaleidoscope Prestained Protein Standard (BioRad, USA).

**Figure 7:**
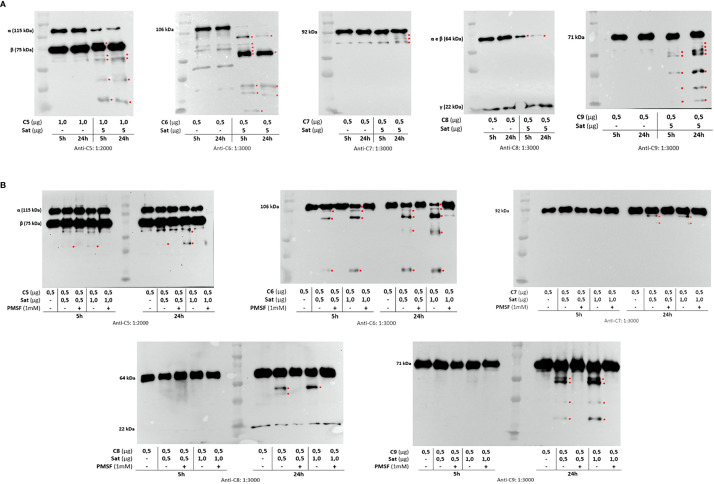
Proteolytic activity of Sat on complement proteins of the terminal pathway. Purified Sat and the human complement proteins were incubated at 37 °C for 5 and 24 h and the incubation products were analyzed by 10% SDS-PAGE, followed by immunodetection of the target proteins with specific complement antibodies (dilutions are indicated under each image) and peroxidase-conjugated anti-goat IgG (1:10,000). Reactivity was detected with the SuperSignal^®^ West Pico Enhanced Chemiluminescent Substrate kit (ThermoFisher Scientific). **(A)** Screening assays using 5 µg of Sat and 0.5 - 1.0 µg of complement proteins. **(B)** Inhibition assays using 0.5 - 1.0 µg of Sat or PMSF-inhibited Sat and 0.5 - 1.0 µg of target proteins. The molecular masses indicated in the images correspond to the chains of each protein after denaturation. Red asterisks indicate the cleavage products. Ladder: Precision Plus Protein Kaleidoscope Prestained Protein Standard (BioRad, USA).

Once the identity of purified Sat was unequivocally confirmed through proteomic analyses (**Supplementary File 2**), proteolytic assays were then performed. We first screened for possible Sat substrates among complement proteins from all three pathways. Sat (5 µg) was incubated with 0.5-1.0 µg of each complement protein for 5 or 24h. Incubation products were analyzed by immunoblotting for cleavage detection, using specific antibodies. As shown in **Figures 5A**, **6A**, **7A**, all proteins but C1q were cleaved by Sat. Cleavages of C2, C5 α-chain, C6 and C8 were apparently more effective, with a clear reduction in the intensity of their remaining chains. In addition, cleavage products of C3b and C4b were also more intense than those observed for C3 and C4, suggesting that the physiological loss of 10 kDa in C3 α-chain and C4 α-chain (corresponding to C3a and C4a, respectively) may expose cleavage sites used by Sat and improve its catalytic action on these proteins.

Dose-dependency experiments using lower amounts of purified Sat and inhibition assays by phenylmethylsulfonyl fluoride (PMSF) further confirmed that cleavages were due to the activity of a serine protease. Sat displayed dose- and time-dependent proteolytic activity since less intense cleavage products were observed when lower concentrations of Sat and shorter incubation periods were used. Complement proteins cleavage was completely abolished when Sat was inhibited by PMSF (**Figures 5B**, **6B**, **7B**).

### *E. coli* DH5α Is Not Killed by Sat-Inactivated NHS

**Figure 8:**
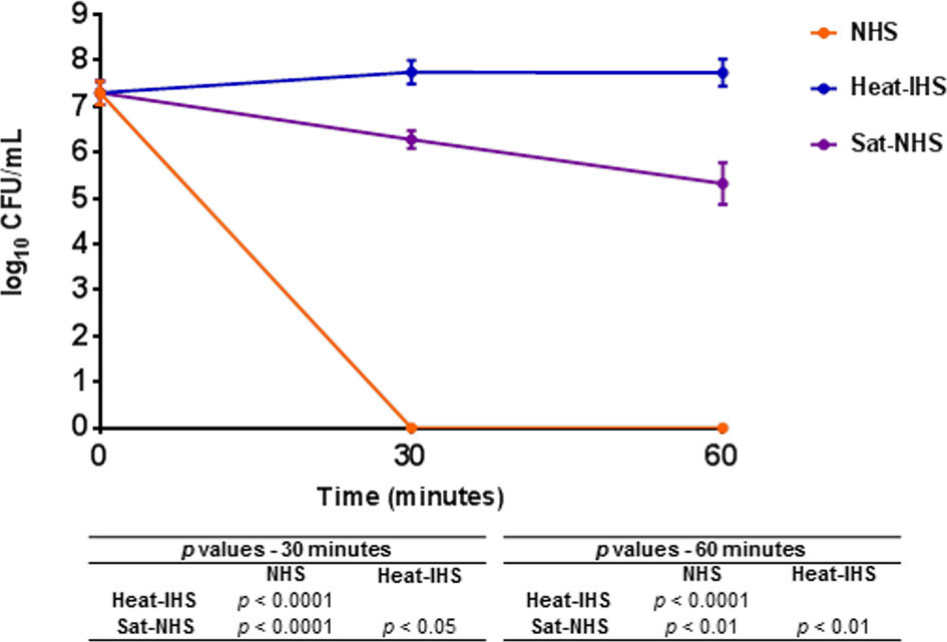
Susceptibility of *E. coli* DH5α in Sat pre-treated normal human serum. DH5α was incubated at 37°C for 30 and 60 min in 25% normal human serum (NHS), 25% heat-inactivated human serum (heat-IHS) or 25% Sat pre-treated normal human serum (Sat-NHS). Data are presented as CFU/mL counts on MacConkey agar plates. Time zero CFU/mL correspond to the initial inocula before contact with NHS, heat-IHS or Sat-NHS. Results obtained for each tested condition at each time point were compared using ANOVA and Tukey’s multiple comparison tests. ns, not significant.

Since Sat was able to cleave complement molecules, we wondered if this activity would protect the non-virulent *E. coli* strain DH5α from complement-mediated killing in the serum. NHS (25%) was preincubated with Sat using the enzyme:substrate ratio tested in the proteolytic assays for cleavage of purified C4. After treatment, DH5α was added to Sat pre-treated NHS (Sat-NHS), NHS or heat-IHS, and incubated at 37°C for 30 and 60 min. As presented in **Figure 8**, *E. coli* DH5α was able to survive in Sat-NHS, and in heat-IHS for 60 min. Survival in Sat-NHS was unequivocally significant, but bacteria were less resistant than those incubated in heat-IHS, possibly due to incomplete inactivation of complement proteins by Sat (**Figure 8**).

### *E. coli* EC071 Derived Genetic Constructions

**Figure 9:**
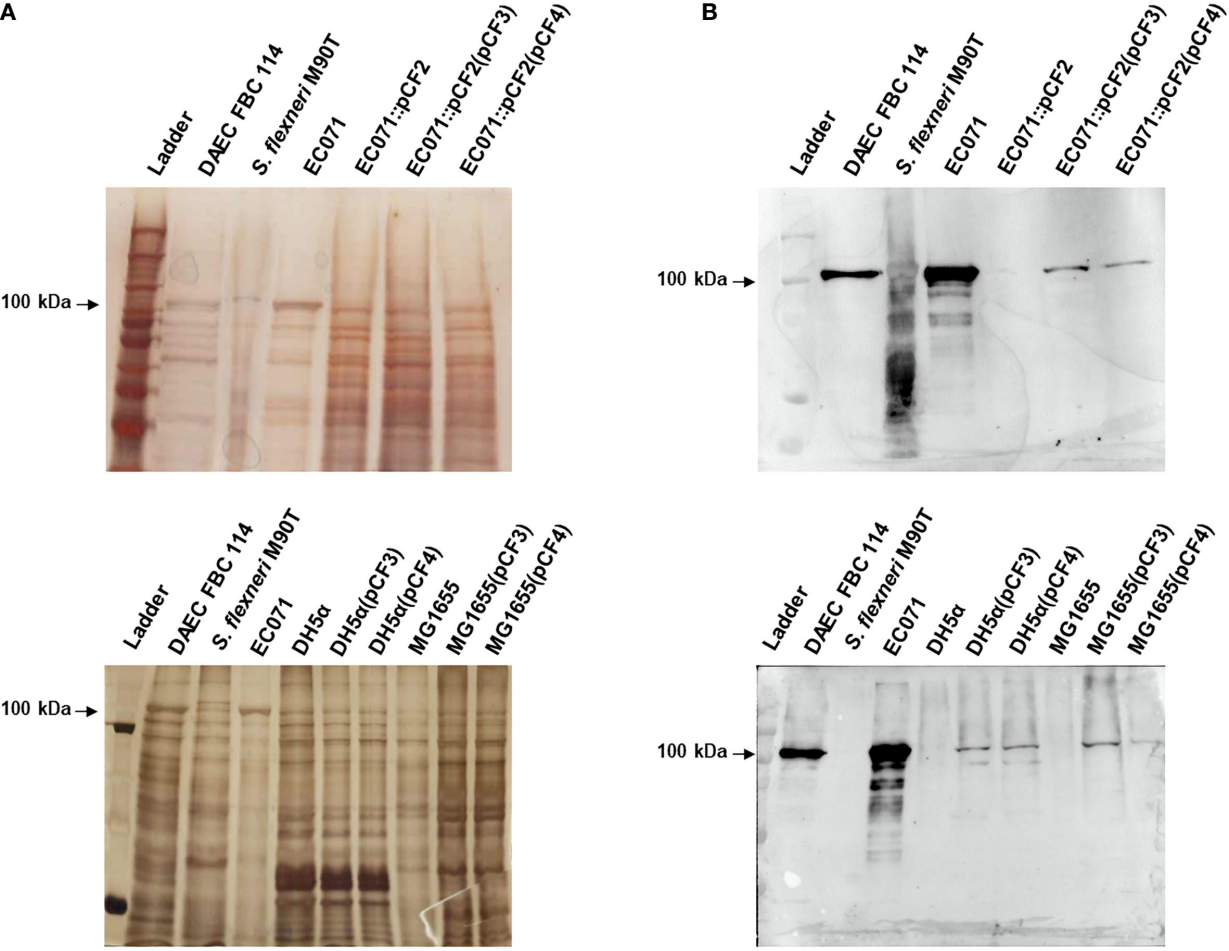
Sat production by EC071, DH5α, MG1655 and derivative strains. TCA-treated LB culture supernatants of all strains, including DAEC FBC114 (positive control) and *S. flexneri* M90T (negative control), were analyzed by 10% SDS-PAGE. **(A)** Silver nitrate-stained gel; **(B)** Immunoblotting with anti-Sat (1:500) and peroxidase-conjugated goat anti-rabbit IgG (1:10,000). Ladder: Precision Plus Protein Kaleidoscope Prestained Protein Standard (BioRad, USA).

To evaluate the role of Sat in a murine sepsis model, genetic constructions and modifications were performed in the wild-type strain EC071. First, *sat* was inactivated by homologous recombination, yielding the *sat* mutant strain EC071::pCF2. In parallel, the *sat* gene was amplified from EC071 genomic DNA and cloned into *pettac*, resulting in the Sat expression clone pCF3. The pCF3 plasmid was used as a template for construction of a site-directed mutant (S256I/S258A), resulting in plasmid CF4, which expresses the inactive serine protease Sat (**Table 1**). Both pCF3 and pCF4 were transformed into *E. coli* DH5α, *E. coli* MG1655 and EC071::pCF2. Expression of Sat was confirmed in all strains by SDS-PAGE and immunoblotting with anti-Sat serum (**Figure 9**). The mutations and corresponding complementation did not affect the growth of the host strains (EC071 and MG1655) (**Supplementary Figure 2**).

**Table 1:** Genetic constructions obtained in this study.

Strain	Description	Sat Production
EC071::pCF2	EC071 *sat* mutant	Absent
EC071::pCF2 (pCF3)	EC071 *sat* mutant complemented with *sat* minimal clone	Active serine protease
EC071::pCF2 (pCF4)	EC071 *sat* mutant complemented with *sat* site-directed mutant	Inactive serine protease
DH5α (pCF3)	DH5α harboring *sat* minimal clone	Active serine protease
DH5α (pCF4)	DH5α harboring *sat* site-directed mutant	Inactive serine protease
MG1655 (pCF3)	MG1655 harboring *sat* minimal clone	Active serine protease
MG1655 (pCF4)	MG1655 harboring *sat* site-directed mutant	Inactive serine protease

### Sat Contributes to the Pathogenesis of Sepsis in Mice

To evaluate the contribution of Sat in the development of BSI and sepsis, EC071, MG1655 and their respective derivative strains were tested in a murine sepsis model, which assesses the lethality of each strain.

Swiss mice were infected subcutaneously with 2 x 10^8^ UFC of each strain. Mice were then observed daily for seven days or until they presented clinical signs that led to a humane endpoint. Survival rates were compared between each experimental group using Fisher’s exact test, with a 95% confidence interval. Initially, the sepsis model was validated by the infection of four animals with the UPEC prototype strain CFT073 (positive control) and four animals with the K-12 *E. coli* strain MG1655 (negative control). While CFT073 caused the death of all the animals up to 36 h post-infection, none of the animals infected with MG1655 died within 7 days post-infection.

**Figure 10:**
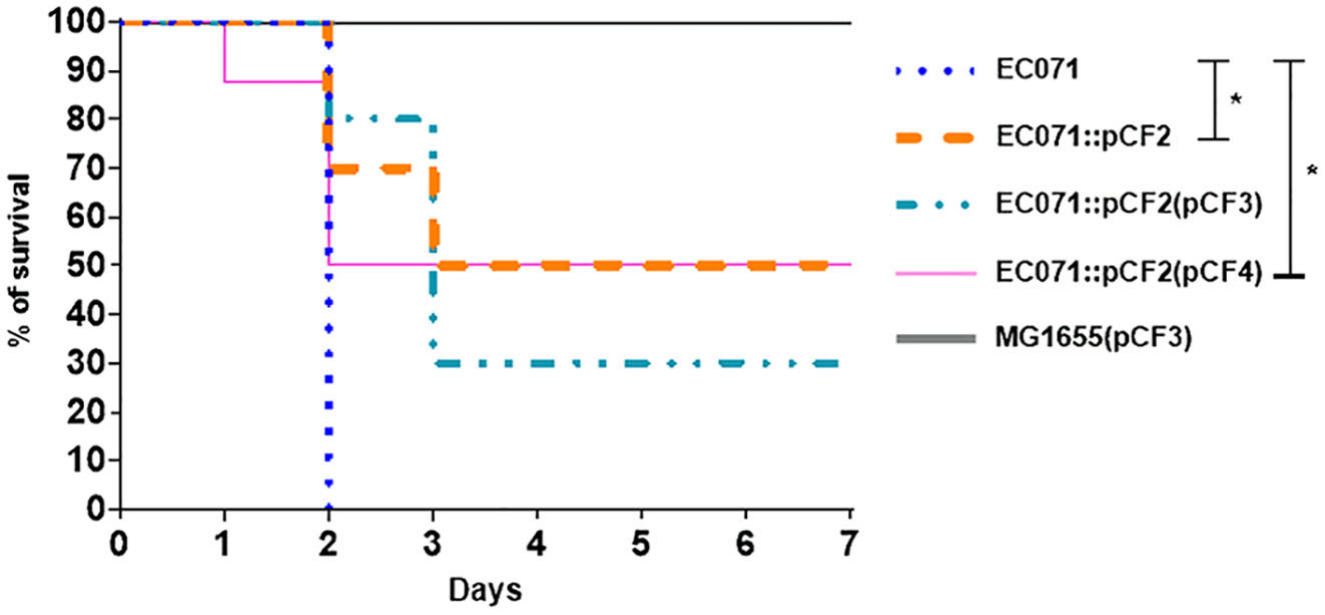
Percentage of survival of the infected animals with EC071 and its derived strains in a murine sepsis model. Female Swiss mice were inoculated subcutaneously with 2 x 10^8^ CFU of EC071 or one of its derivative strains and observed for a maximum of seven days. The animals were observed daily to check for clinical signs resulting from the development of the infection. *p < 0.05

While the wild type strain (EC071) caused the death of all the animals in 48 h, a statistically significant reduction of 50% in lethality was observed in the groups infected with the *sat* mutant strain (EC071::pCF2) and with the non-functional gene complemented strain [EC071::pCF2(pCF4)] (**Figure 10**). The effect was partially restored by the complemented strain [EC071::pCF2(pCF3)], since 70% of the animals within this group died. All the animals infected with MG1655(pCF3) were alive until the seventh day post-infection with no signs of clinical disease. Since death reduction was partially accomplished for the mutant strains and none of the animals infected with MG1655(pCF3) died, our observations suggest that Sat is part of the EC071 virulence arsenal enrolled in sepsis pathogenesis acting in conjunction with other virulence factors.

## Discussion

One common immune system evasion strategy used by *E. coli* causing BSI is the cleavage of complement system proteins by secreted proteases (12). EspP and Pic are secreted proteases of the SPATE family that are capable of cleaving proteins of the complement cascade *in vitro* (12, 13, 15). However, the Sat-encoding gene (*sat*) is among the most frequent SPATE-encoding genes found in ExPEC strains isolated from BSI and is detected in higher frequencies than *espP* and *pic* (22, 23, 25–27, 29–31, 66–68). In addition, Sat causes cytotoxic effects on urinary tract and endothelial cell lines, which may also contribute to the pathogenesis of BSI (38–40, 44).

EC071, the Sat producer prototype strain used in this work, was previously characterized by our group as a phylogroup F strain harboring the genetic markers for intrinsic virulence (ExPEC+) and carrying no other SPATE-encoding gene but *sat* (22). In the present work, Sat production by EC071 was confirmed by immunoblotting using specific anti-Sat serum and this strain was shown to be resistant to the bactericidal activity of NHS. EC071 WGS analysis revealed the presence of other serum-resistance related genes, such as outer membrane proteins-coding genes (*ompX*, *ompTc*, *ompTp* and *nlpI*) and *prc*, which encodes the protease Prc. These virulence traits interact in different manners with complement proteins, interfering with its activation; however, the expression of these factors has not been evaluated in this study and their enrollment in EC071 serum resistance remains unclear. WGS and plasmid profile analysis indicated that *sat* is located in the chromosome, since none of five origins of replication found in the EC071 genome were in the same contig containing the gene *sat*.

The *sat* genetic context in the EC071 genome is in accordance with other authors, since there are no reports concerning the presence of *sat* in plasmids (38, 42, 69).

Purified Sat from EC071 culture supernatant showed a very efficient proteolytic activity on C2, C4, C4b and C6 since degradation products could be observed after 5 h of incubation with a lower concentration of Sat (0.5 µg). The same amount of Sat was sufficient to cleave C3, C3b, C5, C7, C8 and C9, but a longer incubation period was required. These differences may be a consequence of the absence of other putative co-factors absent in MOPS buffer. In a sepsis context, known enzymatic cofactors such as Ca^2+^, glycosaminoglycans, lipids and citrate are available in the plasma (70, 71) and could improve Sat proteolytic activity on complement substrates. Therefore, further kinetic studies are necessary to evaluate Sat activity modulation by such co-factors. Finally, PMSF-inhibited Sat did not cleave any complement protein, confirming that this proteolytic activity relies on its serine protease active site.

**Figure 11:**
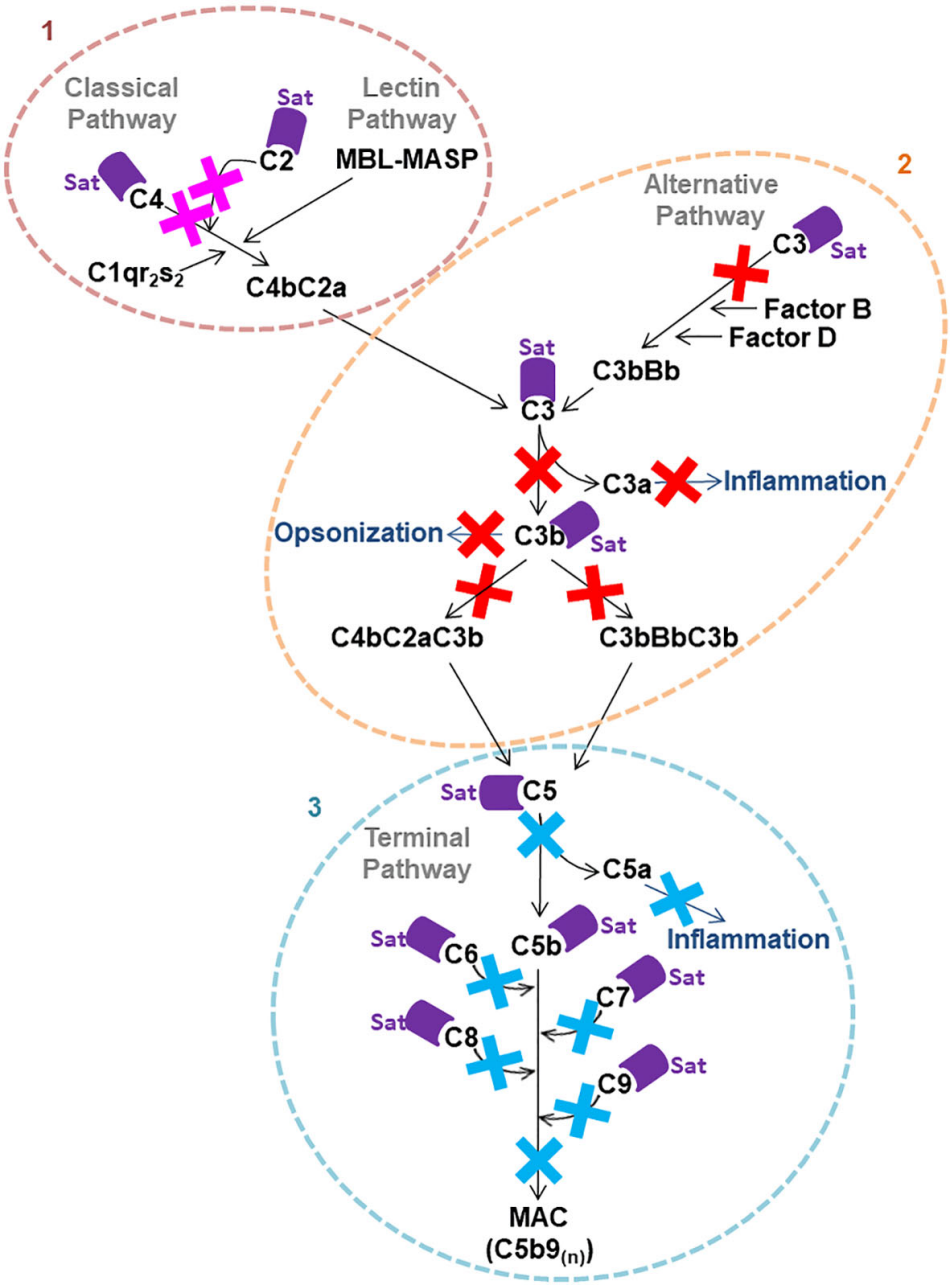
Possible consequences of Sat cleavage of complement system proteins. Proteolysis of complement system proteins by Sat may impact the activation of all three pathways. 1: Direct cleavage of C4 and C2 may inhibit the formation of C4b2a, the C3 convertase of the classical and the lectin pathways. 2: Direct cleavage of C3 may impair the formation of the alternative pathway convertase C3bBb and the C5 convertases C4b2a3b and C3bBb3b. Moreover, cleavage of C3 and C3b may interfere with opsonization. 3: Direct cleavage of C5, C6, C7, C8 and C9, molecules of the common terminal pathway, may restrict MAC assembly on the bacterial surface.

*In vitro* cleavage assays with purified Sat indicated that this serine protease has a broad proteolytic activity on complement proteins and may interfere with the activation of the complement cascade in multiple ways. Direct cleavage of C2, C4 and C4b by Sat may compromise C3 convertase (C4b2a) formation of both the classical and the lectin pathways, preventing the physiological cleavage of C3 into C3a and C3b. Degradation of C3, the central complement factor, and C3b into non-functional fragments may interfere with the activation of all three complement pathways. Besides being involved in opsonization and phagocytosis, C3 and C3b are also enrolled in the formation of the C5 convertases of all pathways (C4b2a3b for the classical and the lectin pathways and C3bBb3b for the alternative pathway). Therefore, cleavage of C3 and C3b by Sat may hinder pathogen elimination by phagocytosis and prevent physiological cleavage of C5 into C5a, an anaphylatoxin involved in the inflammatory response, and C5b, necessary for the initiation of the membrane attack complex (MAC) formation (2, 9, 72). Finally, Sat can suppress complement bactericidal function by direct cleavage of proteins of the common terminal pathway. The direct cleavage of C5 by Sat may interfere with the inflammatory response, and MAC assembly can be compromised by inactivation of C5, C6, C7, C8 and C9 by Sat thereby preventing bacterial elimination by osmotic lysis. **Figure 11** outlines the complement system targets for Sat, and the potential biological consequences resulting from complement inactivation by this protease.

In addition to Sat, the class-2 SPATE Pic was shown to cleave the complement components C2, C3, C3b, C4, C4b and C5 (15, 16). C3, C3b and C5 are also substrates for the class-1 SPATE EspP, produced by EHEC (13). Diverse pathogens of medical importance, including periodontal bacteria, secrete proteolytic enzymes that may impair host defense mechanisms (11). The metalloprotease thermolysin LIC13322 secreted by pathogenic *Leptospira*, NalP produced by *Neisseria meningitidis*, as well as ScpA and SlpB, both produced by *Staphylococcus aureus*, are a few examples of bacterial proteases that target C2, C3, C3b and C5 (73–76).

A smaller number of studies have evaluated the action of bacterial proteases on the common terminal pathway of complement. Thermolysin from pathogenic *Leptospira* (77), the cysteine protease SpeB from *Streptococcus pyogenes* (78) and the serine protease SplB from *Staphylococcus aureus* (76) were shown to degrade C6, C7, C8, and C9, besides C5. Structural similarities shared by the complement proteins acting in the terminal pathway can be an explanation for the fact that all of them are cleaved by Sat and the other two bacterial proteases mentioned above (77, 79, 80).

The fact that Sat cleaved almost every tested complement component raises the hypothesis that such proteolytic activity is not specific. However, C1q was not cleaved in our study, and the study of Dutta et al. (45) showed that other biological substrates, such as pepsin and mucin, are not targeted by Sat as well.

Some structural features of C1q could explain the inability of Sat to cleave this protein. According to Reid (81), C1q is a big molecule (490 kDa) shaped as a “flower bouquet” composed by 18 polypeptide chains (6α, 6β and 6γ). Such a big and complex molecule can turn difficult for bacterial proteases to access possible cleavage sites and for this reason, no degradation can be observed. In fact, the absence of cleavage of C1q by other proteases from different bacteria was observed by other authors (15, 73, 74, 76).

Considering that some complement proteins were more efficiently cleaved by Sat (e.g., C2, C4, C4b and C6) and *E. coli* DH5α survived in Sat-treated human serum in lower rates than in heat-inactivated human serum, it is plausible to presume that the classical, the lectin and the terminal pathways are more efficiently inhibited by Sat than the alternative pathway. However, additional assays are required to assess the specific effects of Sat on each complement activation pathway.

To assess the effects caused by Sat regarding the immune system, EC071 and the derivative strains obtained in this study were assayed in a mouse model of sepsis to assess the lethality of each strain. Considering that a BSI usually results from a primary infection, bacteria were inoculated *via* subcutaneous injection to simulate the occurrence of a resulting BSI from an extraintestinal infection, where bacteria would have to overcome the barriers of this site to access the bloodstream. If another route of inoculation was used, such as intraperitoneal or intravenous, the bloodstream access step would be skipped. Also, the lethality model of infection used in our work has been widely employed since its report by Picard et al. (64) in a way to investigate the role of putative virulence factors of extraintestinal *E. coli* strains. A statistically significant reduction of 50% in lethality was observed for the *sat* mutant strain and its complementation with a site-directed mutated *sat* clone. However, a partial restoration of the lethality with the wild-type strain was observed in animals infected with the *sat* mutant strain complemented with an active *sat* minimal clone, since only 70% of the mice died. The production of Sat by the pCF3 construction (containing the active *sat* minimal clone) was apparently distinct from that observed in the wild-type strain, as observed in the immunoblotting of culture supernatants (**Figure 9**). The lower Sat production could be a possible explanation for the partial restoration of lethality observed for EC071::pCF2(pCF3). Thus, we conclude that Sat is important for EC071 lethality in this mouse model and is involved in sepsis pathogenesis caused by this strain. A similar result was observed by Dutra et al. (82) when the ExPEC strain F5, a Pic-producing ExPEC, and its respective *pic* mutant were tested in a murine sepsis model. While all animals infected with the wild type strain died, all animals infected with the mutant strain survived, suggesting that Pic is also involved in the pathogenesis of sepsis.

In our study, *E. coli* MG1655(pCF3) was also tested in the murine model of sepsis, but all animals infected with this strain survived. From these data, there is evidence that Sat works in conjunction with other virulence factors produced by EC071 for the establishment and dissemination of the infection. Other authors have observed that infection severity is related to the presence of different combinations of virulence genes such as adhesins, protectins and iron uptake systems, and these traits are important for colonization, immune evasion, and nutrient acquisition for bacterial survival (64, 83–88). For that reason, it would be of interest to verify how the presence/absence of *sat* in strains with different genetic backgrounds would impact in the infected animals survival using this murine model of sepsis.

The subcutaneous route of inoculation used in our murine model highlights that the cytotoxic effects of Sat (38–45) are also important for bacteria to access the bloodstream, since Sat causes endothelial damages (44). However, since our murine model of infection evaluates lethality only, further studies are necessary to fully understand the action of Sat in the bloodstream components.

**Figure 12:**
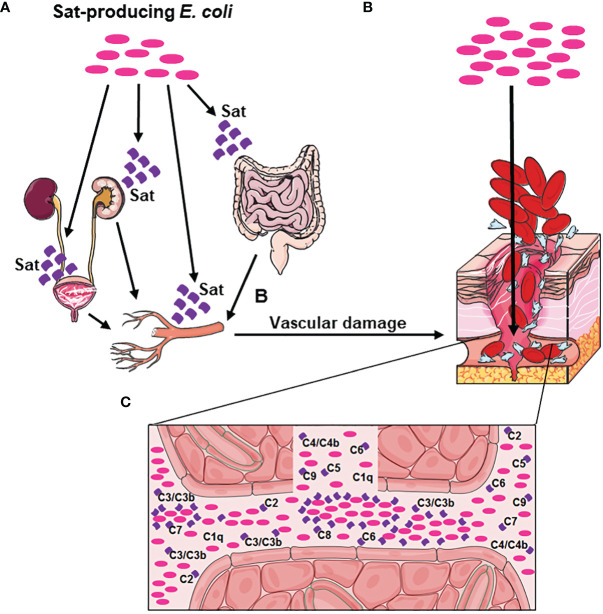
Hypothetical model of Sat enrollment in BSI and sepsis pathogenesis. **(A)** Sat-producing *E. coli* present in the urinary tract or in the intestinal tract secretes Sat, causing cellular damage in the bladder, kidneys, or intestine. This damage allows the bacteria to move forward to the blood vessels, whose endothelial cells are also susceptible to Sat cytotoxic action. **(B)** Vascular damage caused by Sat allows *E. coli* to access the bloodstream. **(C)** Sat secretion in the bloodstream protects *E. coli* from complement killing, by direct cleavage of C2, C3, C3b, C4, C4b, C5, C6, C7, C8 and C9. This evasion mechanism allows Sat-producing *E. coli* to multiply and disseminate in the bloodstream and consequently reach other organs such as the spleen and the liver, where new sites of infection can be established and a magnification of the immune response can occur, facilitating the progression of the disease to sepsis.

Considering the data presented herein and previously published studies about the biological activities of Sat (38–45), a hypothetical model for the role of Sat in *E. coli* sepsis is presented in **Figure 12**. Altogether, these results suggest that Sat may play a dual role in the infection by allowing bacterial accession to the bloodstream after endothelial damage and by locally protecting the pathogen against complement-mediated killing.

Finally, the importance of Sat in the pathogenesis of BSI caused by *E. coli* gives light to the fact that this protease is an important target for the development of vaccines and anti-virulence drugs, either for prevention or treatment of different ExPEC infections, as an alternative to the increasing antibiotic resistance rates.

## Data Availability Statement

The authors confirm that the data supporting the findings of this study are available within the article and its supplementary materials. The raw data are available in the Butantan Institute Repository at https://repositorio.butantan.gov.br/handle/butantan/3947.

## Ethics Statement

The animal study was reviewed and approved by Ethics Committee on Animal Use of the Butantan Institute (CEUAIB protocol number 5743060220).

## Author Contributions

Conceived and designed the experiments: CF, RS, DP, JB, IH, AB, and WE. Performed the experiments: CF, RS, DP, JB, AB, and WE. Analyzed the data: CF, RS, RR, DP, JB, IH, AB, and WE. Contributed reagents/materials/analysis tools: CF, RS, RR, DP, IH, AB, and WE. Wrote the paper: CF, AB, and WE. All authors contributed to the article and approved the submitted version.

## Funding

This study was supported by the São Paulo Research Foundation (FAPESP grant 2017/14821-7) and in part by the Coordenação de Aperfeiçoamento de Pessoal de Nível Superior - Brasil (CAPES) - Finance Code 001. CAF was recipient of scholarships from Conselho Nacional de Desenvolvimento Científico e Tecnológico (142053/2015-5 and 141887/2019-2) and CAPES/PDSE (88881.190211/2018-01). The funders had no role in study design, data collection and analysis, decision to publish, or preparation of the manuscript.

## Conflict of Interest

The authors declare that the research was conducted in the absence of any commercial or financial relationships that could be construed as a potential conflict of interest.

## Publisher’s Note

All claims expressed in this article are solely those of the authors and do not necessarily represent those of their affiliated organizations, or those of the publisher, the editors and the reviewers. Any product that may be evaluated in this article, or claim that may be made by its manufacturer, is not guaranteed or endorsed by the publisher.
