# Supplementary material for: Secreted Autotransporter Toxin (Sat) Mediates Innate Immune System Evasion
Source: Front Immunol. 2022 Feb 17;13:844878. doi: 10.3389/fimmu.2022.844878 (PMC8891578; doi:10.3389/fimmu.2022.844878)
Supplement: Supplementary file 1 [file DataSheet_1.pdf]

COVID-19 Information

Public health information (CDC)

Research information (NIH)

SARS-CoV-2 data (NCBI)

Prevention and treatment information (HHS)

Español

BLAST ® » blastp suite » results for RID-YG87CM3U01R

**Job Title**	Sat EC071 ...
**RID**	YG87CM3U01R Search expires on 01-20 21:32 pm
**Program**	BLASTP
**Database**	nr
**Query ID**	lcl|Query_342596
**Description**	None ...
**Molecule type**	amino acid
**Query Length**	1296

Descriptions

Description	Scientific Name	Max Score	Total Score	Query Cover	E value	Per. Ident	Acc. Len	Accession
serine protease autotransporter toxin Sat [Escherichia coli]	Escherichia coli	2631	2631	99%	0.0	100.00%	1295	WP_001034082.1
TPA: serine protease autotransporter toxin Sat [Escherichia coli]	Escherichia coli	2630	2630	99%	0.0	99.92%	1295	HAI6125783.1
serine protease autotransporter toxin Sat [Escherichia coli]	Escherichia coli	2629	2629	99%	0.0	99.85%	1295	WP_021579527.1
serine protease autotransporter toxin Sat [Escherichia coli]	Escherichia coli	2629	2629	99%	0.0	99.92%	1295	WP_142457960.1
serine protease autotransporter toxin Sat [Escherichia coli]	Escherichia coli	2629	2629	99%	0.0	99.92%	1295	EFK6366304.1
serine protease autotransporter toxin Sat [Escherichia coli]	Escherichia coli	2629	2629	99%	0.0	99.92%	1295	WP_114145544.1
TPA: serine protease autotransporter toxin Sat [Escherichia coli]	Escherichia coli	2629	2629	99%	0.0	99.92%	1295	HBJ6073103.1
serine protease autotransporter toxin Sat [Enterobacteriaceae]	Enterobacteriaceae	2629	2629	99%	0.0	99.85%	1295	WP_126896457.1
TPA: serine protease autotransporter toxin Sat [Escherichia coli]	Escherichia coli	2629	2629	99%	0.0	99.85%	1295	HAJ1961408.1
serine protease autotransporter toxin Sat [Enterobacteriaceae]	Enterobacteriaceae	2629	2629	99%	0.0	99.92%	1295	WP_001034083.1
serine protease autotransporter toxin Sat [Escherichia coli]	Escherichia coli	2628	2628	99%	0.0	99.92%	1295	WP_001034084.1
Aecreted auto transpoter toxin [Escherichia coli str. 'clone D i2']	Escherichia coli str. 'clone D i2'	2628	2628	99%	0.0	99.92%	1299	AER85851.1
serine protease autotransporter toxin Sat [Escherichia coli]	Escherichia coli	2628	2628	99%	0.0	99.85%	1295	WP_033864944.1
serine protease autotransporter toxin Sat [Escherichia coli]	Escherichia coli	2628	2628	99%	0.0	99.85%	1295	EGD9592729.1
TPA: serine protease autotransporter toxin Sat [Escherichia coli]	Escherichia coli	2628	2628	99%	0.0	99.92%	1295	HAL2735930.1
serine protease autotransporter toxin Sat [Escherichia coli]	Escherichia coli	2628	2628	99%	0.0	99.92%	1295	WP_167580541.1
serine protease autotransporter toxin Sat [Escherichia coli]	Escherichia coli	2628	2628	99%	0.0	99.92%	1295	WP_128430364.1
TPA: serine protease autotransporter toxin Sat [Escherichia coli]	Escherichia coli	2628	2628	99%	0.0	99.85%	1295	HAL2502625.1
serine protease autotransporter toxin Sat [Shigella sonnei]	Shigella sonnei	2628	2628	99%	0.0	99.92%	1295	EHI3940979.1
autotransporter outer membrane beta-barrel domain-containing protein [Escherichia coli]	Escherichia coli	2628	2628	99%	0.0	99.85%	1295	WP_219054700.1
serine protease autotransporter toxin Sat [Escherichia coli]	Escherichia coli	2628	2628	99%	0.0	99.85%	1295	MBI9726529.1
TPA: serine protease autotransporter toxin Sat [Escherichia coli]	Escherichia coli	2628	2628	99%	0.0	99.92%	1295	HBB7276045.1
serine protease autotransporter toxin Sat [Escherichia coli]	Escherichia coli	2628	2628	99%	0.0	99.85%	1295	WP_001556799.1
TPA: serine protease autotransporter toxin Sat [Escherichia coli]	Escherichia coli	2628	2628	99%	0.0	99.85%	1295	HBC7568876.1
TPA: serine protease autotransporter toxin Sat [Escherichia coli]	Escherichia coli	2628	2628	99%	0.0	99.85%	1295	HAH4707744.1
serine protease autotransporter toxin Sat [Escherichia coli]	Escherichia coli	2628	2628	99%	0.0	99.85%	1295	WP_021525200.1

Description	Scientific Name	Max Score	Total Score	Query Cover	E value	Per. Ident	Acc. Len	Accession
TPA: serine protease autotransporter toxin Sat [Escherichia coli]	Escherichia coli	2628	2628	99%	0.0	99.85%	1295	HBA1304970.1
serine protease autotransporter toxin Sat [Escherichia coli]	Escherichia coli	2627	2627	99%	0.0	99.85%	1295	WP_021545254.1
serine protease autotransporter toxin Sat [Escherichia coli]	Escherichia coli	2627	2627	99%	0.0	99.85%	1295	WP_113341376.1
TPA: serine protease autotransporter toxin Sat [Escherichia coli]	Escherichia coli	2627	2627	99%	0.0	99.85%	1295	HAX8501378.1
TPA: serine protease autotransporter toxin Sat [Escherichia coli]	Escherichia coli	2627	2627	99%	0.0	99.85%	1295	HBC8781562.1
serine protease autotransporter toxin Sat [Escherichia coli]	Escherichia coli	2627	2627	99%	0.0	99.85%	1295	WP_049074636.1
serine protease autotransporter toxin Sat [Escherichia coli]	Escherichia coli	2627	2627	99%	0.0	99.85%	1295	WP_023150719.1
serine protease sat autotransporter [Escherichia coli 1-110-08_S4_C1]	Escherichia coli 1-110-08_S4_C1	2627	2627	99%	0.0	99.85%	1295	EYE09558.1
serine protease autotransporter toxin Sat [Escherichia coli]	Escherichia coli	2627	2627	99%	0.0	99.85%	1295	WP_021521406.1
serine protease autotransporter toxin Sat [Escherichia coli]	Escherichia coli	2627	2627	99%	0.0	99.85%	1295	MBB0498836.1
TPA: serine protease autotransporter toxin Sat [Escherichia coli]	Escherichia coli	2627	2627	99%	0.0	99.85%	1295	HAP0967861.1
serine protease autotransporter toxin Sat [Escherichia coli]	Escherichia coli	2627	2627	99%	0.0	99.85%	1295	WP_149846178.1
serine protease autotransporter toxin Sat [Escherichia coli]	Escherichia coli	2627	2627	99%	0.0	99.85%	1295	WP_060667427.1
TPA: serine protease autotransporter toxin Sat [Escherichia coli]	Escherichia coli	2627	2627	99%	0.0	99.85%	1295	HBI9439922.1
TPA: serine protease autotransporter toxin Sat [Escherichia coli]	Escherichia coli	2627	2627	99%	0.0	99.85%	1295	HBI7936509.1
autotransporter outer membrane beta-barrel domain-containing protein [Escherichia coli]	Escherichia coli	2627	2627	99%	0.0	99.85%	1295	WP_225394784.1
serine protease autotransporter toxin Sat [Escherichia coli]	Escherichia coli	2627	2627	99%	0.0	99.85%	1295	EHC2729660.1
TPA: serine protease autotransporter toxin Sat [Escherichia coli]	Escherichia coli	2627	2627	99%	0.0	99.85%	1295	HAI5392434.1
TPA: serine protease autotransporter toxin Sat [Escherichia coli]	Escherichia coli	2627	2627	99%	0.0	99.85%	1295	HAL3252735.1
serine protease autotransporter toxin Sat [Escherichia coli]	Escherichia coli	2627	2627	99%	0.0	99.85%	1295	WP_139837327.1
serine protease autotransporter toxin Sat [Escherichia coli]	Escherichia coli	2627	2627	99%	0.0	99.85%	1295	WP_053901632.1
TPA: serine protease autotransporter toxin Sat [Escherichia coli]	Escherichia coli	2627	2627	99%	0.0	99.92%	1295	HAI3750294.1
serine protease autotransporter toxin Sat [Escherichia coli]	Escherichia coli	2627	2627	99%	0.0	99.85%	1295	WP_113956116.1
TPA: serine protease autotransporter toxin Sat [Escherichia coli]	Escherichia coli	2627	2627	99%	0.0	99.85%	1295	HAY5190267.1
serine protease autotransporter toxin Sat [Escherichia coli]	Escherichia coli	2627	2627	99%	0.0	99.85%	1295	MBS8788038.1
serine protease autotransporter toxin Sat [Escherichia coli]	Escherichia coli	2627	2627	99%	0.0	99.92%	1295	WP_064232224.1
serine protease autotransporter toxin Sat [Escherichia coli]	Escherichia coli	2627	2627	99%	0.0	99.85%	1295	WP_118909407.1
serine protease autotransporter toxin Sat [Escherichia coli]	Escherichia coli	2627	2627	99%	0.0	99.85%	1295	WP_115766409.1
serine protease autotransporter toxin Sat [Escherichia coli]	Escherichia coli	2627	2627	99%	0.0	99.85%	1295	WP_001611046.1
TPA: serine protease autotransporter toxin Sat [Escherichia coli]	Escherichia coli	2627	2627	99%	0.0	99.85%	1295	HBC4963495.1
serine protease autotransporter toxin Sat [Escherichia coli]	Escherichia coli	2627	2627	99%	0.0	99.85%	1295	EFH8304360.1
TPA: serine protease autotransporter toxin Sat [Escherichia coli O25b:H4-ST131]	Escherichia coli O25b:H4-ST131	2627	2627	99%	0.0	99.85%	1295	HAN2555901.1
serine protease autotransporter toxin Sat [Escherichia coli]	Escherichia coli	2627	2627	99%	0.0	99.85%	1295	MBI9242867.1
TPA: serine protease autotransporter toxin Sat [Escherichia coli]	Escherichia coli	2627	2627	99%	0.0	99.92%	1295	HAM4370941.1
serine protease autotransporter toxin Sat [Escherichia coli]	Escherichia coli	2627	2627	99%	0.0	99.85%	1295	WP_059319927.1
serine protease autotransporter toxin Sat [Escherichia coli]	Escherichia coli	2627	2627	99%	0.0	99.85%	1295	WP_113495884.1
serine protease autotransporter toxin Sat [Escherichia coli]	Escherichia coli	2627	2627	99%	0.0	99.85%	1295	WP_116991798.1
serine protease autotransporter toxin Sat [Escherichia coli]	Escherichia coli	2627	2627	99%	0.0	99.92%	1294	WP_140402642.1
serine protease autotransporter toxin Sat [Escherichia coli]	Escherichia coli	2627	2627	99%	0.0	99.85%	1295	WP_169783511.1
serine protease autotransporter toxin Sat [Escherichia coli]	Escherichia coli	2627	2627	99%	0.0	99.85%	1295	EEY5188439.1
TPA: serine protease autotransporter toxin Sat [Escherichia coli]	Escherichia coli	2627	2627	99%	0.0	99.85%	1295	HAG7649195.1

Description	Scientific Name	Max Score	Total Score	Query Cover	E value	Per. Ident	Acc. Len	Accession
TPA: serine protease autotransporter toxin Sat [Escherichia coli]	Escherichia coli	2627	2627	99%	0.0	99.85%	1295	HBB1193796.1
serine protease autotransporter toxin Sat [Escherichia coli]	Escherichia coli	2627	2627	99%	0.0	99.85%	1295	WP_181203932.1
TPA: serine protease autotransporter toxin Sat [Escherichia coli]	Escherichia coli	2627	2627	99%	0.0	99.85%	1295	HAH6736077.1
TPA: serine protease autotransporter toxin Sat [Escherichia coli]	Escherichia coli	2627	2627	99%	0.0	99.92%	1294	HAN3395805.1
TPA: serine protease autotransporter toxin Sat [Escherichia coli]	Escherichia coli	2627	2627	99%	0.0	99.85%	1295	HAO7397004.1
TPA: serine protease autotransporter toxin Sat [Escherichia coli]	Escherichia coli	2627	2627	99%	0.0	99.85%	1295	HAI3867277.1
TPA: serine protease autotransporter toxin Sat [Escherichia coli]	Escherichia coli	2627	2627	99%	0.0	99.85%	1295	HBA4320359.1
TPA: serine protease autotransporter toxin Sat [Escherichia coli]	Escherichia coli	2627	2627	99%	0.0	99.85%	1295	HAG9275443.1
TPA: serine protease autotransporter toxin Sat [Escherichia coli MVAST158]	Escherichia coli MVAST158	2627	2627	99%	0.0	99.85%	1295	HAX0142153.1
serine protease autotransporter toxin Sat [Escherichia coli]	Escherichia coli	2627	2627	99%	0.0	99.85%	1295	WP_155951915.1
autotransporter outer membrane beta-barrel domain-containing protein [Escherichia coli]	Escherichia coli	2627	2627	99%	0.0	99.92%	1295	WP_225390149.1
autotransporter outer membrane beta-barrel domain-containing protein [Escherichia coli]	Escherichia coli	2627	2627	99%	0.0	99.85%	1295	WP_217822457.1
serine protease autotransporter toxin Sat [Shigella flexneri]	Shigella flexneri	2627	2627	99%	0.0	99.85%	1295	EFP7778397.1
TPA: serine protease autotransporter toxin Sat [Escherichia coli]	Escherichia coli	2627	2627	99%	0.0	99.85%	1295	HAM6401014.1
serine protease autotransporter toxin Sat [Escherichia coli]	Escherichia coli	2627	2627	99%	0.0	99.85%	1295	WP_094316666.1
serine protease autotransporter toxin Sat [Escherichia coli]	Escherichia coli	2627	2627	99%	0.0	99.85%	1295	MBB0854235.1
serine protease autotransporter toxin Sat [Escherichia coli]	Escherichia coli	2627	2627	99%	0.0	99.85%	1295	NPP26331.1
autotransporter outer membrane beta-barrel domain-containing protein [Escherichia coli]	Escherichia coli	2627	2627	99%	0.0	99.85%	1295	WP_199351549.1
TPA: serine protease autotransporter toxin Sat [Escherichia coli]	Escherichia coli	2627	2627	99%	0.0	99.85%	1295	HBA1072022.1
autotransporter outer membrane beta-barrel domain-containing protein [Escherichia coli]	Escherichia coli	2627	2627	99%	0.0	99.92%	1299	OSP32553.1
TPA: serine protease autotransporter toxin Sat [Escherichia coli]	Escherichia coli	2627	2627	99%	0.0	99.85%	1295	HBD2267896.1
TPA: serine protease autotransporter toxin Sat [Escherichia coli]	Escherichia coli	2627	2627	99%	0.0	99.85%	1295	HAX7888640.1
TPA: serine protease autotransporter toxin Sat [Escherichia coli]	Escherichia coli	2627	2627	99%	0.0	99.85%	1295	HAL3865995.1
TPA: serine protease autotransporter toxin Sat [Escherichia coli]	Escherichia coli	2627	2627	99%	0.0	99.77%	1295	HBA4313454.1
serine protease autotransporter toxin Sat [Escherichia coli]	Escherichia coli	2627	2627	99%	0.0	99.85%	1295	WP_048228344.1
TPA: serine protease autotransporter toxin Sat [Escherichia coli]	Escherichia coli	2627	2627	99%	0.0	99.85%	1295	HBM2060318.1
TPA: serine protease autotransporter toxin Sat [Escherichia coli]	Escherichia coli	2627	2627	99%	0.0	99.85%	1295	HAJ3750798.1
TPA: serine protease autotransporter toxin Sat [Escherichia coli]	Escherichia coli	2627	2627	99%	0.0	99.85%	1295	HBC6041500.1
serine protease autotransporter toxin Sat [Escherichia coli]	Escherichia coli	2627	2627	99%	0.0	99.85%	1295	MSI96964.1
serine protease autotransporter toxin Sat [Escherichia coli]	Escherichia coli	2627	2627	99%	0.0	99.85%	1295	MBI9784956.1
serine protease autotransporter toxin Sat [Escherichia coli]	Escherichia coli	2627	2627	99%	0.0	99.85%	1295	WP_181199831.1
TPA: serine protease autotransporter toxin Sat [Escherichia coli]	Escherichia coli	2627	2627	99%	0.0	99.85%	1295	HBE4050048.1
TPA: serine protease autotransporter toxin Sat [Escherichia coli]	Escherichia coli	2627	2627	99%	0.0	99.85%	1295	HBK1370968.1

Graphic Summary

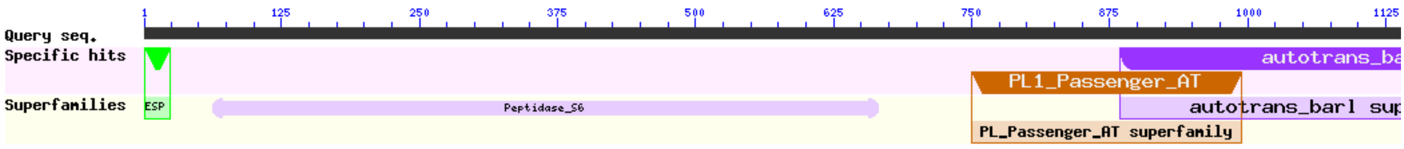

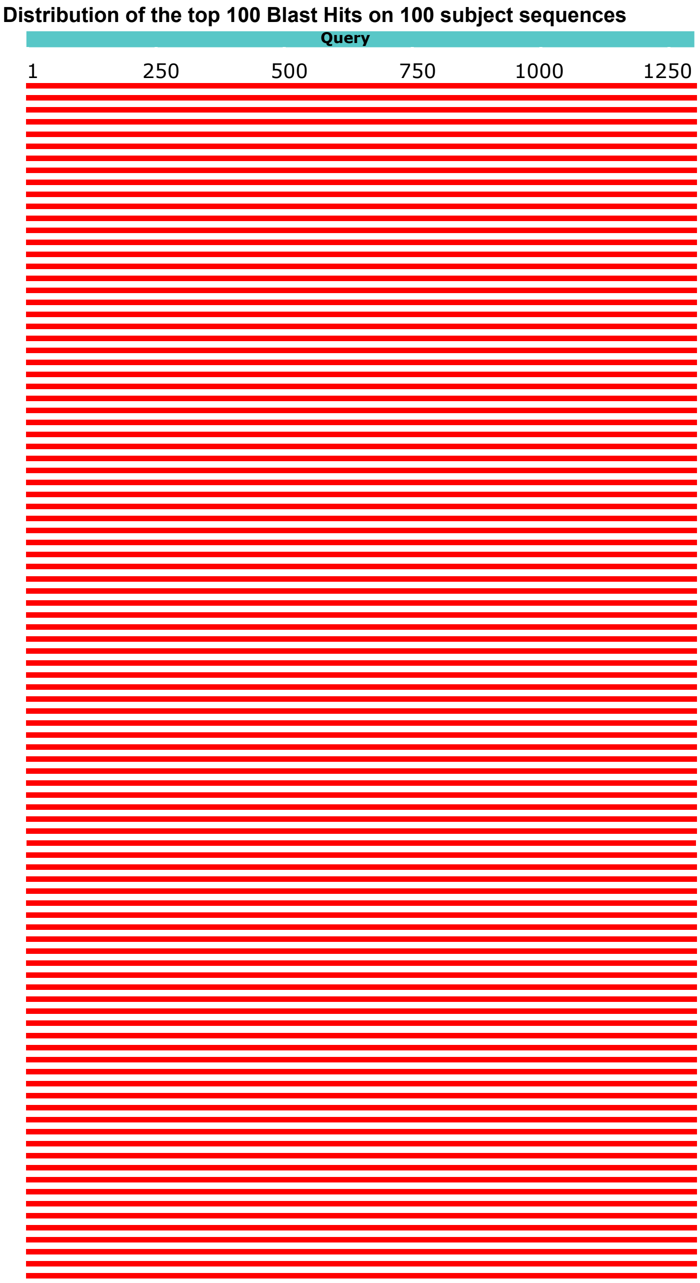

Alignments

Alignment view Query-anchored with dots for identities Line length: 90 Restore defaults

Query range 1: 1 to 90

```
Query           1     MNKIYSLKYSAATGGLIAVSELAKRVSGKTNRKLVATMLSLAVAGTVNAANIDISNVWARDYLDLAQNKGIFQPGATDVTITLKNGDKFS  90
WP_001034082.1  1     ..........................................................................................  90
HAI6125783.1    1     ..........................................................................................  90
WP_021579527.1  1     ..........................................................................................  90
WP_142457960.1  1     ..........................................................................................  90
EFK6366304.1    1     ..........................................................................................  90
WP_114145544.1  1     ..........................................................................................  90
HBJ6073103.1    1     ..........................................................................................  90
WP_126896457.1  1     ..........................................................................................  90
HAJ1961408.1    1     ..........................................................................................  90
WP_001034083.1  1     ..........................................................................................  90
WP_001034084.1  1     ..........................................................................................  90
AER85851.1      5     ..........................................................................................  94
WP_033864944.1  1     ..........................................................................................  90
EGD9592729.1    1     ..........................................................................................  90
HAL2735930.1    1     ..........................................................................................  90
WP_167580541.1  1     ..........................................................................................  90
WP_128430364.1  1     ..........................................................................................  90
HAL2502625.1    1     ..........................................................................................  90
EHI3940979.1    1     ..........................................................................................  90
WP_219054700.1  1     ..........................................................................................  90
MBI9726529.1    1     ..........................................................................................  90
HBB7276045.1    1     ..........................................................................................  90
WP_001556799.1  1     ..........................................................................................  90
HBC7568876.1    1     ..........................................................................................  90
HAH4707744.1    1     ..........................................................................................  90
WP_021525200.1  1     ..........................................................................................  90
HBA1304970.1    1     ..........................................................................................  90
WP_021545254.1  1     ..........................................................................................  90
WP_113341376.1  1     ..........................................................................................  90
HAX8501378.1    1     ..........................................................................................  90
HBC8781562.1    1     ..........................................................................................  90
WP_049074636.1  1     ...........T..............................................................................  90
WP_023150719.1  1     ..........................................................................................  90
EYE09558.1      1     ..........................................................................................  90
WP_021521406.1  1     ..........................................................................................  90
MBB0498836.1    1     ..........................................................................................  90
HAP0967861.1    1     ..........................................................................................  90
WP_149846178.1  1     ..........................................................................................  90
WP_060667427.1  1     ..........................................................................................  90
HBI9439922.1    1     ..........................................................................................  90
HBI7936509.1    1     ..........................................................................................  90
```

```
WP_225394784.1  1     ..........................................................................................  90
EHC2729660.1    1     ..........................................................................................  90
HAI5392434.1    1     ..........................................................................................  90
HAL3252735.1    1     ..................I.......................................................................  90
WP_139837327.1  1     ..........................................................................................  90
WP_053901632.1  1     ..........................................................................................  90
HAI3750294.1    1     ..........................................................................................  90
WP_113956116.1  1     ..........................................................................................  90
HAY5190267.1    1     ..........................................................................................  90
MBS8788038.1    1     ..........................................................................................  90
WP_064232224.1  1     ..........................................................................................  90
WP_118909407.1  1     ..........................................................................................  90
WP_115766409.1  1     ..........................................................................................  90
WP_001611046.1  1     ..........................................................................................  90
HBC4963495.1    1     ..................A.......................................................................  90
EFH8304360.1    1     ......................................................................................N...  90
HAN2555901.1    1     ..........................................................................................  90
MBI9242867.1    1     ..........................................................................................  90
HAM4370941.1    1     ..........................................................................................  90
WP_059319927.1  1     ..........................................................................................  90
WP_113495884.1  1     ..........................................................................................  90
WP_116991798.1  1     ..........................................................................................  90
WP_140402642.1  1     ..........................................................................................  90
WP_169783511.1  1     ..........................................................................................  90
EEY5188439.1    1     ..........................................................................................  90
HAG7649195.1    1     ..........................................................................................  90
HBB1193796.1    1     ..........................................................................................  90
WP_181203932.1  1     ..........................................................................................  90
HAH6736077.1    1     ..........................................................................................  90
HAN3395805.1    1      .........................................................................................  89
HAO7397004.1    1     ..........................................................................................  90
HAI3867277.1    1     ..........................................................................................  90
HBA4320359.1    1     ..........................................................................................  90
HAG9275443.1    1     ..........................................................................................  90
HAX0142153.1    1     ..........................................................................................  90
WP_155951915.1  1     ..........................................................................................  90
WP_225390149.1  1     ............................................D.............................................  90
WP_217822457.1  1     ..........................................................................................  90
EFP7778397.1    1     ..........................................................................................  90
HAM6401014.1    1     ..........................................................................................  90
WP_094316666.1  1     ..........................................................................................  90
MBB0854235.1    1     ..........................................................................................  90
NPP26331.1      1     ..........................................................................................  90
WP_199351549.1  1     ..........................................................................................  90
HBA1072022.1    1     ..........................................................................................  90
OSP32553.1      5     ..........................................................................................  94
HBD2267896.1    1     ..........................................................................................  90
HAX7888640.1    1     ..........................................................................................  90
HAL3865995.1    1     ..........................................................................................  90
HBA4313454.1    1     ..........................................................................................  90
WP_048228344.1  1     ..........................................................................................  90
HBM2060318.1    1     ..........................................................................................  90
HAJ3750798.1    1     ..........................................................................................  90
HBC6041500.1    1     ..........................................................................................  90
MSI96964.1      1     ..........................................................................................  90
MBI9784956.1    1     ..........................................................................................  90
WP_181199831.1  1     ..........................................................................................  90
HBE4050048.1    1     ..........................................................................................  90
HBK1370968.1    1     ..........................................................................................  90
```

Query range 2: 91 to 180

```
Query           91    FHNLSIPDFSGAAASGAATAIGGSYSVTVAHNKKNPQAAETQVYAQSSYKVVDRRNSNDFEIQRLNKFVVETVGATPAETNPTTYSDALE  180
WP_001034082.1  91    ..........................................................................................  180
HAI6125783.1    91    ..........................................................................................  180
WP_021579527.1  91    ..........................................................................................  180
WP_142457960.1  91    ..........................................................................................  180
EFK6366304.1    91    ..........................................................................................  180
WP_114145544.1  91    ..........................................................................................  180
HBJ6073103.1    91    ..........................................................................................  180
WP_126896457.1  91    ..........................................................................................  180
HAJ1961408.1    91    ..........................................................................................  180
WP_001034083.1  91    ..........................................................................................  180
WP_001034084.1  91    ..........................................................................................  180
AER85851.1      95    ..........................................................................................  184
WP_033864944.1  91    ..........................................................................................  180
EGD9592729.1    91    ..........................................................................................  180
HAL2735930.1    91    ..........................................................................................  180
WP_167580541.1  91    ..........................................................................................  180
WP_128430364.1  91    ..........................................................................................  180
HAL2502625.1    91    ..........................................................................................  180
EHI3940979.1    91    ..........................................................................................  180
WP_219054700.1  91    ..........................................................................................  180
MBI9726529.1    91    ............................................S.............................................  180
HBB7276045.1    91    ..........................................................................................  180
WP_001556799.1  91    ..........................................................................................  180
HBC7568876.1    91    ..........................................................................................  180
HAH4707744.1    91    ..........................................................................................  180
WP_021525200.1  91    ..........................................................................................  180
HBA1304970.1    91    ..........................................................................................  180
WP_021545254.1  91    ..........................................................................................  180
WP_113341376.1  91    ..........................................................................................  180
HAX8501378.1    91    ..........................................................................................  180
HBC8781562.1    91    ..........................................................................................  180
WP_049074636.1  91    ..........................................................................................  180
WP_023150719.1  91    ..........................................................................................  180
EYE09558.1      91    ..........................................................................................  180
WP_021521406.1  91    ..........................................................................................  180
MBB0498836.1    91    ..........................................................................................  180
HAP0967861.1    91    ..........................................................................................  180
WP_149846178.1  91    ..........................................................................................  180
WP_060667427.1  91    ..........................................................................................  180
HBI9439922.1    91    ..........................................................................................  180
HBI7936509.1    91    ..........................................................................................  180
WP_225394784.1  91    ..........................................................................................  180
EHC2729660.1    91    ..........................................................................................  180
HAI5392434.1    91    ..........................................................................................  180
HAL3252735.1    91    ..........................................................................................  180
WP_139837327.1  91    ..........................................................................................  180
WP_053901632.1  91    ..........................................................................................  180
HAI3750294.1    91    ..........................................................................................  180
WP_113956116.1  91    ..........................................................................................  180
HAY5190267.1    91    ..........................................................................................  180
MBS8788038.1    91    .............................................................................T............  180
WP_064232224.1  91    ..........................................................................................  180
WP_118909407.1  91    ..........................................................................................  180
WP_115766409.1  91    ..........................................................................................  180
WP_001611046.1  91    ..........................................................................................  180
HBC4963495.1    91    ..........................................................................................  180
EFH8304360.1    91    ..........................................................................................  180
HAN2555901.1    91    ..........................................................................................  180
MBI9242867.1    91    ..........................................................................................  180
HAM4370941.1    91    ..........................................................................................  180
WP_059319927.1  91    ..........................................................................................  180
WP_113495884.1  91    ..........................................................................................  180
WP_116991798.1  91    ..........................................................................................  180
WP_140402642.1  91    ..........................................................................................  180
WP_169783511.1  91    ..........................................................................................  180
```

```
EEY5188439.1    91    ..........................................................................................  180
HAG7649195.1    91    ..........................................................................................  180
HBB1193796.1    91    ..........................................................................................  180
WP_181203932.1  91    ..........................................................................................  180
HAH6736077.1    91    ..........................................................................................  180
HAN3395805.1    90    ..........................................................................................  179
HAO7397004.1    91    ..........................................................................................  180
HAI3867277.1    91    ...I......................................................................................  180
HBA4320359.1    91    ..........................................................................................  180
HAG9275443.1    91    ..........................................................................................  180
HAX0142153.1    91    ..........................................................................................  180
WP_155951915.1  91    ..........................................................................................  180
WP_225390149.1  91    ..........................................................................................  180
WP_217822457.1  91    ..........................................................................................  180
EFP7778397.1    91    ..........................................................................................  180
HAM6401014.1    91    ..........................................................................................  180
WP_094316666.1  91    ..........................................................................................  180
MBB0854235.1    91    ..........................................................................................  180
NPP26331.1      91    ..........................................................................................  180
WP_199351549.1  91    ...........................................................................P..............  180
HBA1072022.1    91    ..........................................................................................  180
OSP32553.1      95    ..........................................................................................  184
HBD2267896.1    91    ..........................................................................................  180
HAX7888640.1    91    ..........................................................................................  180
HAL3865995.1    91    ..........................................................................................  180
HBA4313454.1    91    ..........................................................................................  180
WP_048228344.1  91    ..........................................................................................  180
HBM2060318.1    91    ...........V..............................................................................  180
HAJ3750798.1    91    .............................V............................................................  180
HBC6041500.1    91    ..........................................................................................  180
MSI96964.1      91    ..........................................................................................  180
MBI9784956.1    91    ..........................................................................................  180
WP_181199831.1  91    ..........................................................................................  180
HBE4050048.1    91    ..........................................................................................  180
HBK1370968.1    91    ..........................................................................................  180
```

Query range 3: 181 to 270

```
Query           181   RYGIVTSDGSKKIIGFRAGSGGTSFINGESKISTNSAYSHDLLSASLFEVTQWDSYGMMIYKNDKTFRNLEIFGDSGSGAYLYDNKLEKW  270
WP_001034082.1  181   ..........................................................................................  270
HAI6125783.1    181   ..........................................................................................  270
WP_021579527.1  181   ...................................T......................................................  270
WP_142457960.1  181   ..........................................................................................  270
EFK6366304.1    181   ..........................................................................................  270
WP_114145544.1  181   ..........................................................................................  270
HBJ6073103.1    181   ..........................................................................................  270
WP_126896457.1  181   ..........................................................................................  270
HAJ1961408.1    181   ..........................................................................................  270
WP_001034083.1  181   ..........................................................................................  270
WP_001034084.1  181   ..........................................................................................  270
AER85851.1      185   ..........................................................................................  274
WP_033864944.1  181   ..........................................................................................  270
EGD9592729.1    181   ..........................................................................................  270
HAL2735930.1    181   ..........................................................................................  270
WP_167580541.1  181   ..........................................................................................  270
WP_128430364.1  181   ..........................................................................................  270
HAL2502625.1    181   ..........................................................................................  270
EHI3940979.1    181   ..........................................................................................  270
WP_219054700.1  181   ..........................................................................................  270
MBI9726529.1    181   ..........................................................................................  270
HBB7276045.1    181   ..........................................................................................  270
WP_001556799.1  181   ..........................................................................................  270
HBC7568876.1    181   ..........................................................................................  270
HAH4707744.1    181   ..........................................................................................  270
WP_021525200.1  181   ..........................................................................................  270
HBA1304970.1    181   ..........................................................................................  270
WP_021545254.1  181   ..........................................................................................  270
WP_113341376.1  181   ..........................................................................................  270
HAX8501378.1    181   ..........................................................................................  270
HBC8781562.1    181   ..........................................................................................  270
WP_049074636.1  181   ..........................................................................................  270
WP_023150719.1  181   ..........................................................................................  270
EYE09558.1      181   ..........................................................................................  270
WP_021521406.1  181   ..........................................................................................  270
MBB0498836.1    181   ..........................................................................................  270
HAP0967861.1    181   ..........................................................................................  270
WP_149846178.1  181   ..........................................................................................  270
WP_060667427.1  181   ..........................................................................................  270
HBI9439922.1    181   ..........................................................................................  270
HBI7936509.1    181   ..........................................................................................  270
WP_225394784.1  181   ..........................................................................................  270
EHC2729660.1    181   ..........................................................................................  270
HAI5392434.1    181   ..........................................................................................  270
HAL3252735.1    181   ..........................................................................................  270
WP_139837327.1  181   ..........................................................................................  270
WP_053901632.1  181   ..........................................................................................  270
HAI3750294.1    181   ..........................................................................................  270
WP_113956116.1  181   ..........................................................................................  270
HAY5190267.1    181   ..........................................................................................  270
MBS8788038.1    181   ..........................................................................................  270
WP_064232224.1  181   ..........................................................................................  270
WP_118909407.1  181   .............................................................................P............  270
WP_115766409.1  181   ..........................................................................................  270
WP_001611046.1  181   ..........................................................................................  270
HBC4963495.1    181   ..........................................................................................  270
EFH8304360.1    181   ..........................................................................................  270
HAN2555901.1    181   ..........................................................................................  270
MBI9242867.1    181   ..........................................................................................  270
HAM4370941.1    181   ..........................................................................................  270
WP_059319927.1  181   ..........................................................................................  270
WP_113495884.1  181   ..........................................................................................  270
WP_116991798.1  181   ..........................................................................................  270
WP_140402642.1  181   ..........................................................................................  270
WP_169783511.1  181   ..........................................................................................  270
EEY5188439.1    181   ..........................................................................................  270
HAG7649195.1    181   ..........................................................................................  270
HBB1193796.1    181   ..........................................................................................  270
WP_181203932.1  181   ......................................................................................I...  270
HAH6736077.1    181   ..........................................................................................  270
HAN3395805.1    180   ..........................................................................................  269
HAO7397004.1    181   ..........................................................................................  270
HAI3867277.1    181   ..........................................................................................  270
HBA4320359.1    181   ..........................................................................................  270
HAG9275443.1    181   ..........................................................................................  270
HAX0142153.1    181   ..........................................................................................  270
WP_155951915.1  181   ..........................................................................................  270
WP_225390149.1  181   ..........................................................................................  270
WP_217822457.1  181   ..........................................................................................  270
EFP7778397.1    181   ..........................................................................................  270
HAM6401014.1    181   ..........................................................................................  270
WP_094316666.1  181   ..........................................................................................  270
MBB0854235.1    181   ..........................................................................................  270
NPP26331.1      181   ..........................................................................................  270
WP_199351549.1  181   ..........................................................................................  270
HBA1072022.1    181   ..........................................................................................  270
OSP32553.1      185   ..........................................................................................  274
HBD2267896.1    181   ..........................................................................................  270
HAX7888640.1    181   ..........................................................................................  270
```

```
HAL3865995.1    181  ....................................E.....................................................  270
HBA4313454.1    181  ..........................................................................................  270
WP_048228344.1  181  ..........................................................................................  270
HBM2060318.1    181  ..........................................................................................  270
HAJ3750798.1    181  ..........................................................................................  270
HBC6041500.1    181  ..........................................................................................  270
MSI96964.1      181  ..........................................................................................  270
MBI9784956.1    181  ..........................................................................................  270
WP_181199831.1  181  ..........................................................................................  270
HBE4050048.1    181  ..........................................................................................  270
HBK1370968.1    181  ..........................................................................................  270
```

Query range 4: 271 to 360

```
Query           271  VLVGTTHGIASVNGDQLTWITKYNDKLVSELKDTYSHKINLNGNNVTIKNTDITLHQNNADTTGTQEKITKDKDIVFTNGGNVLFKDNLD  360
WP_001034082.1  271  ..........................................................................................  360
HAI6125783.1    271  ..........................................................................................  360
WP_021579527.1  271  ..........................................................................................  360
WP_142457960.1  271  ..........................................................................................  360
EFK6366304.1    271  ..........................................................................................  360
WP_114145544.1  271  ..........................................................................................  360
HBJ6073103.1    271  ..........................................................................................  360
WP_126896457.1  271  ..........................................................................................  360
HAJ1961408.1    271  ..........................................................................................  360
WP_001034083.1  271  ..........................................................................................  360
WP_001034084.1  271  ..........................................................................................  360
AER85851.1      275  ..........................................................................................  364
WP_033864944.1  271  ..........................................................................................  360
EGD9592729.1    271  ..........................................................................................  360
HAL2735930.1    271  ..........................................................................................  360
WP_167580541.1  271  ..........................................................................................  360
WP_128430364.1  271  ..........................................................................................  360
HAL2502625.1    271  ..........................................................................................  360
EHI3940979.1    271  .................................................K........................................  360
WP_219054700.1  271  ..........................................................................................  360
MBI9726529.1    271  ..........................................................................................  360
HBB7276045.1    271  ..........................................................................................  360
WP_001556799.1  271  ..........................................................................................  360
HBC7568876.1    271  ..........................................................................................  360
HAH4707744.1    271  ..........................................................................................  360
WP_021525200.1  271  ..........................................................................................  360
HBA1304970.1    271  ..........................................................................................  360
WP_021545254.1  271  ..........................................................................................  360
WP_113341376.1  271  I.........................................................................................  360
HAX8501378.1    271  .........T................................................................................  360
HBC8781562.1    271  ........................................................................................F.  360
WP_049074636.1  271  ..........................................................................................  360
WP_023150719.1  271  ..........................................................................................  360
EYE09558.1      271  ..........................................................................................  360
WP_021521406.1  271  ..........................................................................................  360
MBB0498836.1    271  ..........................................................................................  360
HAP0967861.1    271  ..........................................................................................  360
WP_149846178.1  271  ..........................................................................................  360
WP_060667427.1  271  ..........................................................................................  360
HBI9439922.1    271  ..........................................................................................  360
HBI7936509.1    271  ..........................................................................................  360
WP_225394784.1  271  ..........................................................................................  360
EHC2729660.1    271  ..........................................................................................  360
HAI5392434.1    271  ..........................................................................................  360
HAL3252735.1    271  ..........................................................................................  360
WP_139837327.1  271  ..........................................................................................  360
WP_053901632.1  271  ..........................................................................................  360
HAI3750294.1    271  ..........................................................................................  360
WP_113956116.1  271  .............................K............................................................  360
HAY5190267.1    271  ..........................................................................................  360
MBS8788038.1    271  ..........................................................................................  360
WP_064232224.1  271  ..........................................................................................  360
WP_118909407.1  271  ..........................................................................................  360
WP_115766409.1  271  ..........................................................................................  360
WP_001611046.1  271  ..........................................................................................  360
HBC4963495.1    271  ..........................................................................................  360
EFH8304360.1    271  ..........................................................................................  360
HAN2555901.1    271  ..........................................................................................  360
MBI9242867.1    271  ..........................................................................................  360
HAM4370941.1    271  .................................................................L........................  360
WP_059319927.1  271  ..........................................................................................  360
WP_113495884.1  271  ..............E...........................................................................  360
WP_116991798.1  271  ..........................................................................................  360
WP_140402642.1  271  ..........................................................................................  360
WP_169783511.1  271  ..........................................................................................  360
EEY5188439.1    271  ..........................................................................................  360
HAG7649195.1    271  .....P....................................................................................  360
HBB1193796.1    271  ..........................................................................................  360
WP_181203932.1  271  ..........................................................................................  360
HAH6736077.1    271  ..........................................................................................  360
HAN3395805.1    270  ..........................................................................................  359
HAO7397004.1    271  ..........................................................................................  360
HAI3867277.1    271  ..........................................................................................  360
HBA4320359.1    271  ..........................................................................................  360
HAG9275443.1    271  ..........................................................................................  360
HAX0142153.1    271  ..........................................................................................  360
WP_155951915.1  271  .......................................................Y..................................  360
WP_225390149.1  271  ..........................................................................................  360
WP_217822457.1  271  ..........................................................................................  360
EFP7778397.1    271  ..........................................................................................  360
HAM6401014.1    271  ..........................................................................................  360
WP_094316666.1  271  ..........................................................................................  360
MBB0854235.1    271  ..........................................................................................  360
NPP26331.1      271  ...................................................N......................................  360
WP_199351549.1  271  ..........................................................................................  360
HBA1072022.1    271  ........................N.................................................................  360
OSP32553.1      275  ..........................................................................................  364
HBD2267896.1    271  ..........................................................................................  360
HAX7888640.1    271  ..........................................................................................  360
HAL3865995.1    271  ..........................................................................................  360
HBA4313454.1    271  ..........................................................................................  360
WP_048228344.1  271  ..........................................................................................  360
HBM2060318.1    271  ..........................................................................................  360
HAJ3750798.1    271  ..........................................................................................  360
HBC6041500.1    271  ..........................................................................................  360
MSI96964.1      271  ..........................................................................................  360
MBI9784956.1    271  ..........................................................................................  360
WP_181199831.1  271  .........V................................................................................  360
HBE4050048.1    271  ..........................................................................................  360
HBK1370968.1    271  ..........................................................................................  360
```

Query range 5: 361 to 450

```
Query           361  FGSGGIIFDEGHEYNINGQGFTFKGAGIDIGKESIVNWNALYSSDDVLHKIGPGTLNVQKKQGANIKIGEGNVILNEEGTFNNIYLASGN  450
WP_001034082.1  361  ..........................................................................................  450
HAI6125783.1    361  ..........................................................................................  450
WP_021579527.1  361  ..........................................................................................  450
WP_142457960.1  361  ..........................................................................................  450
EFK6366304.1    361  ..........................................................................................  450
WP_114145544.1  361  ..................................................T.......................................  450
HBJ6073103.1    361  ..........................................................................................  450
```

```
WP_126896457.1  361  ..........................................................................................  450
HAJ1961408.1    361  ..........................................................................................  450
WP_001034083.1  361  ..........................................................................................  450
WP_001034084.1  361  ..........................................................................................  450
AER85851.1      365  ..........................................................................................  454
WP_033864944.1  361  ..........................................................................................  450
EGD9592729.1    361  ...........................................R..............................................  450
HAL2735930.1    361  ..........................................................................................  450
WP_167580541.1  361  ..........................................................................................  450
WP_128430364.1  361  ..........................................................................................  450
HAL2502625.1    361  ............................................................R.............................  450
EHI3940979.1    361  ..........................................................................................  450
WP_219054700.1  361  ..........................................................................................  450
MBI9726529.1    361  ..........................................................................................  450
HBB7276045.1    361  ..........................................................................................  450
WP_001556799.1  361  ..........................................................................................  450
HBC7568876.1    361  ..........................................................................................  450
HAH4707744.1    361  ..........................................................................................  450
WP_021525200.1  361  ..........................................................................................  450
HBA1304970.1    361  ..........................................................................................  450
WP_021545254.1  361  ..........................................................................................  450
WP_113341376.1  361  ..........................................................................................  450
HAX8501378.1    361  ..........................................................................................  450
HBC8781562.1    361  ..........................................................................................  450
WP_049074636.1  361  ..........................................................................................  450
WP_023150719.1  361  ..........................................................................................  450
EYE09558.1      361  ..........................................................................................  450
WP_021521406.1  361  ..........................................................................................  450
MBB0498836.1    361  ..........................................................................................  450
HAP0967861.1    361  ..........................................................................................  450
WP_149846178.1  361  ..........................................................................................  450
WP_060667427.1  361  ..........................................................................................  450
HBI9439922.1    361  ..........................................................................................  450
HBI7936509.1    361  ..........................................................................................  450
WP_225394784.1  361  ..........................................................................................  450
EHC2729660.1    361  ..........................................................................................  450
HAI5392434.1    361  ..........................................................................................  450
HAL3252735.1    361  ..........................................................................................  450
WP_139837327.1  361  ..........................................................................................  450
WP_053901632.1  361  ..........................................................................................  450
HAI3750294.1    361  ..............................................................R...........................  450
WP_113956116.1  361  ..........................................................................................  450
HAY5190267.1    361  ..........................................................................................  450
MBS8788038.1    361  ..........................................................................................  450
WP_064232224.1  361  ..........................................................................................  450
WP_118909407.1  361  ..........................................................................................  450
WP_115766409.1  361  ..........................................................................................  450
WP_001611046.1  361  ..........................................................................................  450
HBC4963495.1    361  ..........................................................................................  450
EFH8304360.1    361  ..........................................................................................  450
HAN2555901.1    361  ..........................................................................................  450
MBI9242867.1    361  ..........................................................................................  450
HAM4370941.1    361  ..........................................................................................  450
WP_059319927.1  361  ..........................................................................................  450
WP_113495884.1  361  ..........................................................................................  450
WP_116991798.1  361  ..........................................................................................  450
WP_140402642.1  361  ..........................................................................................  450
WP_169783511.1  361  ..........................................................................................  450
EEY5188439.1    361  ..........................................................................................  450
HAG7649195.1    361  ..........................................................................................  450
HBB1193796.1    361  ..........................................................................................  450
WP_181203932.1  361  ..........................................................................................  450
HAH6736077.1    361  ..........................................................................................  450
HAN3395805.1    360  ..........................................................................................  449
HAO7397004.1    361  ............................................N.............................................  450
HAI3867277.1    361  ..........................................................................................  450
HBA4320359.1    361  ..........................................................................................  450
HAG9275443.1    361  ..........................................................................................  450
HAX0142153.1    361  ..........................................................................................  450
WP_155951915.1  361  ..........................................................................................  450
WP_225390149.1  361  ..........................................................................................  450
WP_217822457.1  361  ..........................................................................................  450
EFP7778397.1    361  ..........................................................................................  450
HAM6401014.1    361  ..........................................................................................  450
WP_094316666.1  361  ..........................................................................................  450
MBB0854235.1    361  ..........................................................................................  450
NPP26331.1      361  ..........................................................................................  450
WP_199351549.1  361  ..........................................................................................  450
HBA1072022.1    361  ..........................................................................................  450
OSP32553.1      365  ..........................................................................................  454
HBD2267896.1    361  ..........................................................................................  450
HAX7888640.1    361  ..........................................................................................  450
HAL3865995.1    361  ..........................................................................................  450
HBA4313454.1    361  ..........................................................................................  450
WP_048228344.1  361  .................S........................................................................  450
HBM2060318.1    361  ..........................................................................................  450
HAJ3750798.1    361  ..........................................................................................  450
HBC6041500.1    361  ..........................................................................................  450
MSI96964.1      361  ..........................................................................................  450
MBI9784956.1    361  ..........................................................................................  450
WP_181199831.1  361  ..........................................................................................  450
HBE4050048.1    361  ..........................................................................................  450
HBK1370968.1    361  ..........................................................................................  450
```

Query range 6: 451 to 540

```
Query           451  GKVILNKDNSLGNDQYAGIFFTKRGGTLDLNGHNQTFTRIAATDDGTTITNSDTTKEAVLAINNEDSYIYHGNINGNIKLTHNINSQDKK  540
WP_001034082.1  451  ..........................................................................................  540
HAI6125783.1    451  ..........................................................................................  540
WP_021579527.1  451  ..........................................................................................  540
WP_142457960.1  451  ..........................................................................................  540
EFK6366304.1    451  ..........................................................................................  540
WP_114145544.1  451  ..........................................................................................  540
HBJ6073103.1    451  .................................................................N........................  540
WP_126896457.1  451  ..........................................................................................  540
HAJ1961408.1    451  ..........................................................................................  540
WP_001034083.1  451  ..........................................................................................  540
WP_001034084.1  451  ..........................................................................................  540
AER85851.1      455  ..........................................................................................  544
WP_033864944.1  451  ..........................................................................................  540
EGD9592729.1    451  ..........................................................................................  540
HAL2735930.1    451  ................................Y.........................................................  540
WP_167580541.1  451  ..........................................................................................  540
WP_128430364.1  451  ..........................................................................................  540
HAL2502625.1    451  ..........................................................................................  540
EHI3940979.1    451  ..........................................................................................  540
WP_219054700.1  451  ..........................................................................................  540
MBI9726529.1    451  ..........................................................................................  540
HBB7276045.1    451  ..........................................................................................  540
WP_001556799.1  451  ..........................................................................................  540
HBC7568876.1    451  ..........................S...............................................................  540
HAH4707744.1    451  ..........................................................................................  540
WP_021525200.1  451  ..........................................................................................  540
HBA1304970.1    451  ..........................................................................................  540
WP_021545254.1  451  ..........................................................................................  540
WP_113341376.1  451  ..........................................................................................  540
HAX8501378.1    451  ..........................................................................................  540
HBC8781562.1    451  ..........................................................................................  540
```

```
WP_049074636.1  451  ..........................................................................................  540
WP_023150719.1  451  ..........................................................................................  540
EYE09558.1      451  ..........................................................................................  540
WP_021521406.1  451  ..........................................................................................  540
MBB0498836.1    451  ..........................................................................................  540
HAP0967861.1    451  ..........................................................................................  540
WP_149846178.1  451  ..........................................................................................  540
WP_060667427.1  451  ..........................................................................................  540
HBI9439922.1    451  ..........................................................................................  540
HBI7936509.1    451  ..........................................................................................  540
WP_225394784.1  451  ..........................................................................................  540
EHC2729660.1    451  ..........................................................................................  540
HAI5392434.1    451  ..........................................................................................  540
HAL3252735.1    451  ..........................................................................................  540
WP_139837327.1  451  ..........................................................................................  540
WP_053901632.1  451  ..........................................................................................  540
HAI3750294.1    451  ..........................................................................................  540
WP_113956116.1  451  ..........................................................................................  540
HAY5190267.1    451  ..........................................................................................  540
MBS8788038.1    451  ..........................................................................................  540
WP_064232224.1  451  ........................C.................................................................  540
WP_118909407.1  451  ..........................................................................................  540
WP_115766409.1  451  ..........................................................................................  540
WP_001611046.1  451  ..........................................................................................  540
HBC4963495.1    451  ..........................................................................................  540
EFH8304360.1    451  ..........................................................................................  540
HAN2555901.1    451  ..........................................................................................  540
MBI9242867.1    451  ..........................................................................................  540
HAM4370941.1    451  ..........................................................................................  540
WP_059319927.1  451  ..........................................................................................  540
WP_113495884.1  451  ..........................................................................................  540
WP_116991798.1  451  ..........................................................................................  540
WP_140402642.1  451  ..........................................................................................  540
WP_169783511.1  451  ..........................................................................................  540
EEY5188439.1    451  ................................Y.........................................................  540
HAG7649195.1    451  ..........................................................................................  540
HBB1193796.1    451  ..........................................................................................  540
WP_181203932.1  451  ..........................................................................................  540
HAH6736077.1    451  ..........................................................................................  540
HAN3395805.1    450  ..........................................................................................  539
HAO7397004.1    451  ..........................................................................................  540
HAI3867277.1    451  ..........................................................................................  540
HBA4320359.1    451  ..........................................................................................  540
HAG9275443.1    451  ..........................................................................................  540
HAX0142153.1    451  ..........................................................................................  540
WP_155951915.1  451  ..........................................................................................  540
WP_225390149.1  451  ..........................................................................................  540
WP_217822457.1  451  ..........................................................................................  540
EFP7778397.1    451  ..........................................................................................  540
HAM6401014.1    451  ..........................................................................................  540
WP_094316666.1  451  ..........................................................................................  540
MBB0854235.1    451  ..............................................................H...........................  540
NPP26331.1      451  ..........................................................................................  540
WP_199351549.1  451  ..........................................................................................  540
HBA1072022.1    451  ..........................................................................................  540
OSP32553.1      455  ..........................................................................................  544
HBD2267896.1    451  ..........................................................................................  540
HAX7888640.1    451  ..........................................................................................  540
HAL3865995.1    451  ..........................................................................................  540
HBA4313454.1    451  ................................Y.........................................................  540
WP_048228344.1  451  ..........................................................................................  540
HBM2060318.1    451  ..........................................................................................  540
HAJ3750798.1    451  ..........................................................................................  540
HBC6041500.1    451  ..........................................................................................  540
MSI96964.1      451  ..........................................................................................  540
MBI9784956.1    451  ..........................................................................................  540
WP_181199831.1  451  ..........................................................................................  540
HBE4050048.1    451  ..........................................................................................  540
HBK1370968.1    451  ..........................................................................................  540
```

Query range 7: 541 to 630

```
Query           541  TNAKLILDGSVNTKNDVEVSNASLTMQGHATEHAIFRSTANHCSLVFLCGTDWVTVLKETESSYNKKFNSDHKSNNQQTSFDQPDWKTGV  630
WP_001034082.1  541  ..........................................................................................  630
HAI6125783.1    541  ..........................................................................................  630
WP_021579527.1  541  ..........................................................................................  630
WP_142457960.1  541  ..........................................................................................  630
EFK6366304.1    541  ..........I...............................................................................  630
WP_114145544.1  541  ..........................................................................................  630
HBJ6073103.1    541  ..........................................................................................  630
WP_126896457.1  541  .............................................................N............................  630
HAJ1961408.1    541  ..........................................................................................  630
WP_001034083.1  541  ..........................................................................................  630
WP_001034084.1  541  ..........................................................................................  630
AER85851.1      545  ..........................................................................................  634
WP_033864944.1  541  ..........................................................................................  630
EGD9592729.1    541  ..........................................................................................  630
HAL2735930.1    541  ..........................................................................................  630
WP_167580541.1  541  ..........................................................................................  630
WP_128430364.1  541  ..........................................................................................  630
HAL2502625.1    541  ..........................................................................................  630
EHI3940979.1    541  ..........................................................................................  630
WP_219054700.1  541  ..........................................................................................  630
MBI9726529.1    541  ..........................................................................................  630
HBB7276045.1    541  ...................................................H......................................  630
WP_001556799.1  541  ..........................................................................................  630
HBC7568876.1    541  ..........................................................................................  630
HAH4707744.1    541  ..........................................................................................  630
WP_021525200.1  541  ..........................................................................................  630
HBA1304970.1    541  ..........................................................................................  630
WP_021545254.1  541  ..........................................................................................  630
WP_113341376.1  541  ..........................................................................................  630
HAX8501378.1    541  ..........................................................................................  630
HBC8781562.1    541  ..........................................................................................  630
WP_049074636.1  541  ..........................................................................................  630
WP_023150719.1  541  ..........................................................................................  630
EYE09558.1      541  ..........................................................................................  630
WP_021521406.1  541  ..........................................................................................  630
MBB0498836.1    541  ..........................................................................................  630
HAP0967861.1    541  ..........................................................................................  630
WP_149846178.1  541  .....................T....................................................................  630
WP_060667427.1  541  ..........................................................................................  630
HBI9439922.1    541  ..........................................................................................  630
HBI7936509.1    541  .....................................................I....................................  630
WP_225394784.1  541  ..........................................................................................  630
EHC2729660.1    541  ..........................................................................................  630
HAI5392434.1    541  ..........................................................................................  630
HAL3252735.1    541  ..........................................................................................  630
WP_139837327.1  541  ..........................................................................................  630
WP_053901632.1  541  ..........I...............................................................................  630
HAI3750294.1    541  ..........................................................................................  630
WP_113956116.1  541  ..........................................................................................  630
HAY5190267.1    541  .................K........................................................................  630
MBS8788038.1    541  ..........................................................................................  630
WP_064232224.1  541  ..........................................................................................  630
WP_118909407.1  541  ..........................................................................................  630
WP_115766409.1  541  ..........................................................................................  630
WP_001611046.1  541  ..........................................................................................  630
```

```
HBC4963495.1    541  ..........................................................................................  630
EFH8304360.1    541  ..........................................................................................  630
HAN2555901.1    541  ..........................................................................................  630
MBI9242867.1    541  ..........................................................................................  630
HAM4370941.1    541  ..........................................................................................  630
WP_059319927.1  541  ..........F...............................................................................  630
WP_113495884.1  541  ..........................................................................................  630
WP_116991798.1  541  .................................................................................E........  630
WP_140402642.1  541  ..........................................................................................  630
WP_169783511.1  541  ..................................................................N.......................  630
EEY5188439.1    541  ..........................................................................................  630
HAG7649195.1    541  ..........................................................................................  630
HBB1193796.1    541  ..........................................................................................  630
WP_181203932.1  541  ..........................................................................................  630
HAH6736077.1    541  ..........................................................................................  630
HAN3395805.1    540  ..........................................................................................  629
HAO7397004.1    541  ..........................................................................................  630
HAI3867277.1    541  ..........................................................................................  630
HBA4320359.1    541  ..........................................................................................  630
HAG9275443.1    541  ..........................................................................................  630
HAX0142153.1    541  ..........................................................................................  630
WP_155951915.1  541  ..........................................................................................  630
WP_225390149.1  541  ..........................................................................................  630
WP_217822457.1  541  ..........................................................................................  630
EFP7778397.1    541  .......................................................................Y..................  630
HAM6401014.1    541  .........................................Y................................................  630
WP_094316666.1  541  ..........................................................................................  630
MBB0854235.1    541  ..........................................................................................  630
NPP26331.1      541  ..........................................................................................  630
WP_199351549.1  541  ..........................................................................................  630
HBA1072022.1    541  ..........................................................................................  630
OSP32553.1      545  ..........................................................................................  634
HBD2267896.1    541  ..........................................................................................  630
HAX7888640.1    541  ..........................................................................................  630
HAL3865995.1    541  ..........................................................................................  630
HBA4313454.1    541  ..........................................................................................  630
WP_048228344.1  541  ..........................................................................................  630
HBM2060318.1    541  ..........................................................................................  630
HAJ3750798.1    541  ..........................................................................................  630
HBC6041500.1    541  ..........................................................................................  630
MSI96964.1      541  ..........................................................................................  630
MBI9784956.1    541  ..........................................................................................  630
WP_181199831.1  541  ..........................................................................................  630
HBE4050048.1    541  ..........................................................................................  630
HBK1370968.1    541  ..........................................................................................  630
```

Query range 8: 631 to 720

```
Query           631  FKFDTLHLNNADFSISRNANVEGNISANKSAITIGDKNAYIDNLAGKNITNNGFDFKQTISTNLSIGETKFTGGITAHNSQIAIGDQAVV  720
WP_001034082.1  631  ..........................................................................................  720
HAI6125783.1    631  ..........................................................................................  720
WP_021579527.1  631  ..........................................................................................  720
WP_142457960.1  631  ..........................................................................................  720
EFK6366304.1    631  ..........................................................................................  720
WP_114145544.1  631  ..........................................................................................  720
HBJ6073103.1    631  ..........................................................................................  720
WP_126896457.1  631  ..........................................................................................  720
HAJ1961408.1    631  ..........................................................................................  720
WP_001034083.1  631  ..........................................................................................  720
WP_001034084.1  631  ..........................................................................................  720
AER85851.1      635  ..........................................................................................  724
WP_033864944.1  631  ..........................................................................................  720
EGD9592729.1    631  ..........................................................................................  720
HAL2735930.1    631  ..........................................................................................  720
WP_167580541.1  631  .......................................................................................V..  720
WP_128430364.1  631  ..........................................................................................  720
HAL2502625.1    631  ..........................................................................................  720
EHI3940979.1    631  ..........................................................................................  720
WP_219054700.1  631  ..........................................................................................  720
MBI9726529.1    631  ..........................................................................................  720
HBB7276045.1    631  ..........................................................................................  720
WP_001556799.1  631  ..........................................................................................  720
HBC7568876.1    631  ..........................................................................................  720
HAH4707744.1    631  ..........................................................................................  720
WP_021525200.1  631  ..........................................................................................  720
HBA1304970.1    631  ..........................................................................................  720
WP_021545254.1  631  ..........................................................................................  720
WP_113341376.1  631  ..........................................................................................  720
HAX8501378.1    631  ..........................................................................................  720
HBC8781562.1    631  ..........................................................................................  720
WP_049074636.1  631  ..........................................................................................  720
WP_023150719.1  631  ..........................................................................................  720
EYE09558.1      631  ..........................................................................................  720
WP_021521406.1  631  ..........................................................................................  720
MBB0498836.1    631  .....................................................................R....................  720
HAP0967861.1    631  ..........................................................................................  720
WP_149846178.1  631  ..........................................................................................  720
WP_060667427.1  631  ..........................................................................................  720
HBI9439922.1    631  ..........................................................................................  720
HBI7936509.1    631  ..........................................................................................  720
WP_225394784.1  631  ....................I.....................................................................  720
EHC2729660.1    631  ..........................................................................................  720
HAI5392434.1    631  ..........................................................................................  720
HAL3252735.1    631  ..........................................................................................  720
WP_139837327.1  631  ..........................................................................................  720
WP_053901632.1  631  ..........................................................................................  720
HAI3750294.1    631  ..........................................................................................  720
WP_113956116.1  631  ..........................................................................................  720
HAY5190267.1    631  ..........................................................................................  720
MBS8788038.1    631  ..........................................................................................  720
WP_064232224.1  631  ..........................................................................................  720
WP_118909407.1  631  ..........................................................................................  720
WP_115766409.1  631  ..........................................................................................  720
WP_001611046.1  631  ..........................................................................................  720
HBC4963495.1    631  ..........................................................................................  720
EFH8304360.1    631  ..........................................................................................  720
HAN2555901.1    631  ....................................................................N.....................  720
MBI9242867.1    631  ..........................................................................................  720
HAM4370941.1    631  ..........................................................................................  720
WP_059319927.1  631  ..........................................................................................  720
WP_113495884.1  631  ..........................................................................................  720
WP_116991798.1  631  ..........................................................................................  720
WP_140402642.1  631  ..........................................................................................  720
WP_169783511.1  631  ..........................................................................................  720
EEY5188439.1    631  ..........................................................................................  720
HAG7649195.1    631  ..........................................................................................  720
HBB1193796.1    631  ..........................................................................T...............  720
WP_181203932.1  631  ..........................................................................................  720
HAH6736077.1    631  ..........................................................................................  720
HAN3395805.1    630  ..........................................................................................  719
HAO7397004.1    631  ..........................................................................................  720
HAI3867277.1    631  ..........................................................................................  720
HBA4320359.1    631  ..........................................................................................  720
HAG9275443.1    631  ..........................................................................................  720
HAX0142153.1    631  ..........................................................................................  720
WP_155951915.1  631  ..........................................................................................  720
WP_225390149.1  631  ..........................................................................................  720
WP_217822457.1  631  ..............................................E...........................................  720
```

```
EFP7778397.1    631  ..........................................................................................  720
HAM6401014.1    631  ..........................................................................................  720
WP_094316666.1  631  ..........................................................................................  720
MBB0854235.1    631  ..........................................................................................  720
NPP26331.1      631  ..........................................................................................  720
WP_199351549.1  631  ..........................................................................................  720
HBA1072022.1    631  ..........................................................................................  720
OSP32553.1      635  ..........................................................................................  724
HBD2267896.1    631  ..........................................................................................  720
HAX7888640.1    631  ..........................................................................................  720
HAL3865995.1    631  ..........................................................................................  720
HBA4313454.1    631  ..........................................................................................  720
WP_048228344.1  631  ..........................................................................................  720
HBM2060318.1    631  ..........................................................................................  720
HAJ3750798.1    631  ..........................................................................................  720
HBC6041500.1    631  ................................................................F.........................  720
MSI96964.1      631  ..........................................................................................  720
MBI9784956.1    631  ..........................................................................................  720
WP_181199831.1  631  ..........................................................................................  720
HBE4050048.1    631  ..........................................................................................  720
HBK1370968.1    631  ......................................V...................................................  720
```

Query range 9: 721 to 810

```
Query           721  TLNGATFLNNTPISIDKGAKVIAQNSMFTTKGIDISGELTMMGIPEQNSKTVTPGLHYAADGFRLSGGNANFIARNMASVTGNIYADDAA  810
WP_001034082.1  721  ..........................................................................................  810
HAI6125783.1    721  .........................................................................................S  810
WP_021579527.1  721  ..........................................................................................  810
WP_142457960.1  721  ..........................................................................................  810
EFK6366304.1    721  ..........................................................................................  810
WP_114145544.1  721  ..........................................................................................  810
HBJ6073103.1    721  ..........................................................................................  810
WP_126896457.1  721  ..........................................................................................  810
HAJ1961408.1    721  ..........................................................................................  810
WP_001034083.1  721  ..........................................................................................  810
WP_001034084.1  721  .........................................T................................................  810
AER85851.1      725  ..........................................................................................  814
WP_033864944.1  721  ..........................................................................................  810
EGD9592729.1    721  ..........................................................................................  810
HAL2735930.1    721  ..........................................................................................  810
WP_167580541.1  721  ..........................................................................................  810
WP_128430364.1  721  ......................V...................................................................  810
HAL2502625.1    721  ..........................................................................................  810
EHI3940979.1    721  ..........................................................................................  810
WP_219054700.1  721  ..........................................................................................  810
MBI9726529.1    721  ..........................................................................................  810
HBB7276045.1    721  ..........................................................................................  810
WP_001556799.1  721  ..........................................................................................  810
HBC7568876.1    721  ..........................................................................................  810
HAH4707744.1    721  ..........................................................................................  810
WP_021525200.1  721  .....................M....................................................................  810
HBA1304970.1    721  ..........................................................................................  810
WP_021545254.1  721  ....................I.....................................................................  810
WP_113341376.1  721  ..........................................................................................  810
HAX8501378.1    721  ..........................................................................................  810
HBC8781562.1    721  ..........................................................................................  810
WP_049074636.1  721  ..........................................................................................  810
WP_023150719.1  721  ..........................................................................................  810
EYE09558.1      721  ..........................................................................................  810
WP_021521406.1  721  ..........................................................................................  810
MBB0498836.1    721  .........................................T................................................  810
HAP0967861.1    721  .......................................................F..................................  810
WP_149846178.1  721  ..........................................................................................  810
WP_060667427.1  721  ..........................................................................................  810
HBI9439922.1    721  ..........................................................................................  810
HBI7936509.1    721  ..........................................................................................  810
WP_225394784.1  721  ..........................................................................................  810
EHC2729660.1    721  ..........................................................................................  810
HAI5392434.1    721  ..........................................................................................  810
HAL3252735.1    721  ..........................................................................................  810
WP_139837327.1  721  ..........................................................................................  810
WP_053901632.1  721  ..........................................................................................  810
HAI3750294.1    721  ..........................................................................................  810
WP_113956116.1  721  ..........................................................................................  810
HAY5190267.1    721  ..........................................................................................  810
MBS8788038.1    721  ..........................................................................................  810
WP_064232224.1  721  ..........................................................................................  810
WP_118909407.1  721  ..........................................................................................  810
WP_115766409.1  721  ..........................................................................................  810
WP_001611046.1  721  ..........................................................................................  810
HBC4963495.1    721  ..........................................................................................  810
EFH8304360.1    721  ..........................................................................................  810
HAN2555901.1    721  ..........................................................................................  810
MBI9242867.1    721  ..........................................................................................  810
HAM4370941.1    721  ..........................................................................................  810
WP_059319927.1  721  ..........................................................................................  810
WP_113495884.1  721  ..........................................................................................  810
WP_116991798.1  721  ..........................................................................................  810
WP_140402642.1  721  ..........................................................................................  810
WP_169783511.1  721  ..........................................................................................  810
EEY5188439.1    721  ..........................................................................................  810
HAG7649195.1    721  ..........................................................................................  810
HBB1193796.1    721  ..........................................................................................  810
WP_181203932.1  721  ..........................................................................................  810
HAH6736077.1    721  ..........................................................................................  810
HAN3395805.1    720  ..........................................................................................  809
HAO7397004.1    721  ..........................................................................................  810
HAI3867277.1    721  ..........................................................................................  810
HBA4320359.1    721  ....................L....................T................................................  810
HAG9275443.1    721  ..........................................................................................  810
HAX0142153.1    721  ..........................................................................................  810
WP_155951915.1  721  ..........................................................................................  810
WP_225390149.1  721  ..........................................................................................  810
WP_217822457.1  721  ..........................................................................................  810
EFP7778397.1    721  ..........................................................................................  810
HAM6401014.1    721  ..........................................................................................  810
WP_094316666.1  721  ..........................................................................................  810
MBB0854235.1    721  ..........................................................................................  810
NPP26331.1      721  ..........................................................................................  810
WP_199351549.1  721  ..........................................................................................  810
HBA1072022.1    721  ..........................................................................................  810
OSP32553.1      725  .........................................T................................................  814
HBD2267896.1    721  ..........................................................................................  810
HAX7888640.1    721  ..........................................................................................  810
HAL3865995.1    721  ..........................................................................................  810
HBA4313454.1    721  ..........................................................................................  810
WP_048228344.1  721  ..........................................................................................  810
HBM2060318.1    721  ..........................................................................................  810
HAJ3750798.1    721  ..........................................................................................  810
HBC6041500.1    721  ..........................................................................................  810
MSI96964.1      721  ..........................................................................................  810
MBI9784956.1    721  ..........................................................................................  810
WP_181199831.1  721  ..........................................................................................  810
HBE4050048.1    721  ..........................................................................................  810
HBK1370968.1    721  ..........................................................................................  810
```

Query range 10: 811 to 900

```
Query           811   TITLGQPETETPTISSAYQAWAETLLYGFDTAYRGAITAPKATVSMNNAIWHLNSQSSINRLETKDSMVRFTGDNGKFTTLTVDNLTIDD  900
WP_001034082.1  811   ..........................................................................................  900
HAI6125783.1    811   ..........................................................................................  900
WP_021579527.1  811   ..........................................................................................  900
WP_142457960.1  811   ..........................................................................................  900
EFK6366304.1    811   ..........................................................................................  900
WP_114145544.1  811   ..........................................................................................  900
HBJ6073103.1    811   ..........................................................................................  900
WP_126896457.1  811   ..........................................................................................  900
HAJ1961408.1    811   ......................................................N...................................  900
WP_001034083.1  811   ..........................................................................................  900
WP_001034084.1  811   ..........................................................................................  900
AER85851.1      815   ..........................................................................................  904
WP_033864944.1  811   ..........................................................................................  900
EGD9592729.1    811   ..........................................................................................  900
HAL2735930.1    811   ..........................................................................................  900
WP_167580541.1  811   ..........................................................................................  900
WP_128430364.1  811   ..........................................................................................  900
HAL2502625.1    811   ..........................................................................................  900
EHI3940979.1    811   ..........................................................................................  900
WP_219054700.1  811   ..........................................................................................  900
MBI9726529.1    811   ..........................................................................................  900
HBB7276045.1    811   ..........................................................................................  900
WP_001556799.1  811   ..........................................................................................  900
HBC7568876.1    811   ..........................................................................................  900
HAH4707744.1    811   ..........................................................................................  900
WP_021525200.1  811   ..........................................................................................  900
HBA1304970.1    811   ..........................................................................................  900
WP_021545254.1  811   ..........................................................................................  900
WP_113341376.1  811   ..........................................................................................  900
HAX8501378.1    811   ..........................................................................................  900
HBC8781562.1    811   ..........................................................................................  900
WP_049074636.1  811   ..........................................................................................  900
WP_023150719.1  811   ..........................................................................................  900
EYE09558.1      811   ..........................................................................................  900
WP_021521406.1  811   ..........................................................................................  900
MBB0498836.1    811   ..........................................................................................  900
HAP0967861.1    811   ..........................................................................................  900
WP_149846178.1  811   ..........................................................................................  900
WP_060667427.1  811   ..........................................................................................  900
HBI9439922.1    811   ..........................................................................................  900
HBI7936509.1    811   ..........................................................................................  900
WP_225394784.1  811   ..........................................................................................  900
EHC2729660.1    811   ..........................................................................................  900
HAI5392434.1    811   ..........................................................................................  900
HAL3252735.1    811   ..........................................................................................  900
WP_139837327.1  811   ..........................................................................................  900
WP_053901632.1  811   ..........................................................................................  900
HAI3750294.1    811   ..........................................................................................  900
WP_113956116.1  811   ..........................................................................................  900
HAY5190267.1    811   ..........................................................................................  900
MBS8788038.1    811   ..........................................................................................  900
WP_064232224.1  811   ..........................................................................................  900
WP_118909407.1  811   ..........................................................................................  900
WP_115766409.1  811   ..........................................................................................  900
WP_001611046.1  811   ..........................................................................................  900
HBC4963495.1    811   ..........................................................................................  900
EFH8304360.1    811   ..........................................................................................  900
HAN2555901.1    811   ..........................................................................................  900
MBI9242867.1    811   .............T............................................................................  900
HAM4370941.1    811   ..........................................................................................  900
WP_059319927.1  811   ..........................................................................................  900
WP_113495884.1  811   ..........................................................................................  900
WP_116991798.1  811   ..........................................................................................  900
WP_140402642.1  811   ..........................................................................................  900
WP_169783511.1  811   ..........................................................................................  900
EEY5188439.1    811   ..........................................................................................  900
HAG7649195.1    811   ..........................................................................................  900
HBB1193796.1    811   ..........................................................................................  900
WP_181203932.1  811   ..........................................................................................  900
HAH6736077.1    811   ..........................................................................................  900
HAN3395805.1    810   ..........................................................................................  899
HAO7397004.1    811   ..........................................................................................  900
HAI3867277.1    811   ..........................................................................................  900
HBA4320359.1    811   ..........................................................................................  900
HAG9275443.1    811   ..............L...........................................................................  900
HAX0142153.1    811   ..........................................................................................  900
WP_155951915.1  811   ..........................................................................................  900
WP_225390149.1  811   ..........................................................................................  900
WP_217822457.1  811   ..........................................................................................  900
EFP7778397.1    811   ..........................................................................................  900
HAM6401014.1    811   ..........................................................................................  900
WP_094316666.1  811   ..........................................................................................  900
MBB0854235.1    811   ..........................................................................................  900
NPP26331.1      811   ..........................................................................................  900
WP_199351549.1  811   ..........................................................................................  900
HBA1072022.1    811   ..........................................................................................  900
OSP32553.1      815   ..........................................................................................  904
HBD2267896.1    811   ..........................................................................................  900
HAX7888640.1    811   ..........................................................................................  900
HAL3865995.1    811   ..........................................................................................  900
HBA4313454.1    811   ..........................................................................................  900
WP_048228344.1  811   ..........................................................................................  900
HBM2060318.1    811   ..........................................................................................  900
HAJ3750798.1    811   ..........................................................................................  900
HBC6041500.1    811   ..........................................................................................  900
MSI96964.1      811   ..........................................................................................  900
MBI9784956.1    811   ..........................................................................................  900
WP_181199831.1  811   ..........................................................................................  900
HBE4050048.1    811   ..........................................................................................  900
HBK1370968.1    811   ..........................................................................................  900
```

Query range 11: 901 to 990

```
Query           901   SAFVLRANLAQADQLVVNKSLSGKNNLLLVDFIEKNGNSNGLNIDLVSAPKGTAVDVFKATTRSIGFSDVTPVIEQKNDTDKATWTLIGY  990
WP_001034082.1  901   ..........................................................................................  990
HAI6125783.1    901   ..........................................................................................  990
WP_021579527.1  901   ..........................................................................................  990
WP_142457960.1  901   .........T................................................................................  990
EFK6366304.1    901   ..........................................................................................  990
WP_114145544.1  901   ..........................................................................................  990
HBJ6073103.1    901   ..........................................................................................  990
WP_126896457.1  901   ..........................................................................................  990
HAJ1961408.1    901   ..........................................................................................  990
WP_001034083.1  901   ..........................................................................................  990
WP_001034084.1  901   ..........................................................................................  990
AER85851.1      905   ..........................................................................................  994
WP_033864944.1  901   ...............................................R..........................................  990
EGD9592729.1    901   ..........................................................................................  990
HAL2735930.1    901   ..........................................................................................  990
WP_167580541.1  901   ..........................................................................................  990
WP_128430364.1  901   ..........................................................................................  990
HAL2502625.1    901   ..........................................................................................  990
EHI3940979.1    901   ..........................................................................................  990
WP_219054700.1  901   ..........................................................................................  990
MBI9726529.1    901   ..........................................................................................  990
```

```
HBB7276045.1    901   ..........................................................................................  990
WP_001556799.1  901   ..........................................................................................  990
HBC7568876.1    901   ..........................................................................................  990
HAH4707744.1    901   ..........................................................................................  990
WP_021525200.1  901   ..........................................................................................  990
HBA1304970.1    901   C.........................................................................................  990
WP_021545254.1  901   ..........................................................................................  990
WP_113341376.1  901   ..........................................................................................  990
HAX8501378.1    901   ..........................................................................................  990
HBC8781562.1    901   ..........................................................................................  990
WP_049074636.1  901   ..........................................................................................  990
WP_023150719.1  901   ..........................................................................................  990
EYE09558.1      901   ..........................................................................................  990
WP_021521406.1  901   ..................................................................................T.......  990
MBB0498836.1    901   ..........................................................................................  990
HAP0967861.1    901   ..........................................................................................  990
WP_149846178.1  901   ..........................................................................................  990
WP_060667427.1  901   ..........................................................................................  990
HBI9439922.1    901   ..........................................................................................  990
HBI7936509.1    901   ..........................................................................................  990
WP_225394784.1  901   ..........................................................................................  990
EHC2729660.1    901   ..........................................................................................  990
HAI5392434.1    901   ..........................................................................................  990
HAL3252735.1    901   ..........................................................................................  990
WP_139837327.1  901   ..........................................................................................  990
WP_053901632.1  901   ..........................................................................................  990
HAI3750294.1    901   ..........................................................................................  990
WP_113956116.1  901   ..........................................................................................  990
HAY5190267.1    901   ..........................................................................................  990
MBS8788038.1    901   ..........................................................................................  990
WP_064232224.1  901   ..........................................................................................  990
WP_118909407.1  901   ..........................................................................................  990
WP_115766409.1  901   ...........................................................................K..............  990
WP_001611046.1  901   ..........................................................................................  990
HBC4963495.1    901   ..........................................................................................  990
EFH8304360.1    901   ..........................................................................................  990
HAN2555901.1    901   ..........................................................................................  990
MBI9242867.1    901   ..........................................................................................  990
HAM4370941.1    901   ..........................................................................................  990
WP_059319927.1  901   ..........................................................................................  990
WP_113495884.1  901   ..........................................................................................  990
WP_116991798.1  901   ..........................................................................................  990
WP_140402642.1  901   ..........................................................................................  990
WP_169783511.1  901   ..........................................................................................  990
EEY5188439.1    901   ..........................................................................................  990
HAG7649195.1    901   ..........................................................................................  990
HBB1193796.1    901   ..........................................................................................  990
WP_181203932.1  901   ..........................................................................................  990
HAH6736077.1    901   ..........................................................................................  990
HAN3395805.1    900   ..........................................................................................  989
HAO7397004.1    901   ..........................................................................................  990
HAI3867277.1    901   ..........................................................................................  990
HBA4320359.1    901   ..........................................................................................  990
HAG9275443.1    901   ..........................................................................................  990
HAX0142153.1    901   ............................................E.............................................  990
WP_155951915.1  901   ..........................................................................................  990
WP_225390149.1  901   ..........................................................................................  990
WP_217822457.1  901   ..........................................................................................  990
EFP7778397.1    901   ..........................................................................................  990
HAM6401014.1    901   ..........................................................................................  990
WP_094316666.1  901   ..........................................................................................  990
MBB0854235.1    901   ..........................................................................................  990
NPP26331.1      901   ..........................................................................................  990
WP_199351549.1  901   ..........................................................................................  990
HBA1072022.1    901   ..........................................................................................  990
OSP32553.1      905   ..........................................................................................  994
HBD2267896.1    901   ..........................................................................................  990
HAX7888640.1    901   ..........................................................................................  990
HAL3865995.1    901   ..........................................................................................  990
HBA4313454.1    901   N.........................................................................................  990
WP_048228344.1  901   ..........................................................................................  990
HBM2060318.1    901   ..........................................................................................  990
HAJ3750798.1    901   ..........................................................................................  990
HBC6041500.1    901   ..........................................................................................  990
MSI96964.1      901   ..........................................................................................  990
MBI9784956.1    901   ..........................................................................................  990
WP_181199831.1  901   ..........................................................................................  990
HBE4050048.1    901   ..........................................................................................  990
HBK1370968.1    901   ..........................................................................................  990
```

Query range 12: 991 to 1080

```
Query           991   KSVANADAAKKATLLMSGGYKAFLAEVNNLNKRMGDLRDINGESGAWARIMSGTGSAGGGFSDNYTHVQVGADNKHELDGLDLFTGVTMT  1080
WP_001034082.1  991   ..........................................................................................  1080
HAI6125783.1    991   ..........................................................................................  1080
WP_021579527.1  991   ..........................................................................................  1080
WP_142457960.1  991   ..........................................................................................  1080
EFK6366304.1    991   ..........................................................................................  1080
WP_114145544.1  991   ..........................................................................................  1080
HBJ6073103.1    991   ..........................................................................................  1080
WP_126896457.1  991   ..........................................................................................  1080
HAJ1961408.1    991   ..........................................................................................  1080
WP_001034083.1  991   ..........................................................................................  1080
WP_001034084.1  991   ..........................................................................................  1080
AER85851.1      995   ..........................................................................................  1084
WP_033864944.1  991   ..........................................................................................  1080
EGD9592729.1    991   ..........................................................................................  1080
HAL2735930.1    991   ..........................................................................................  1080
WP_167580541.1  991   ..........................................................................................  1080
WP_128430364.1  991   ..........................................................................................  1080
HAL2502625.1    991   ..........................................................................................  1080
EHI3940979.1    991   ..........................................................................................  1080
WP_219054700.1  991   ..........................................................................................  1080
MBI9726529.1    991   ..........................................................................................  1080
HBB7276045.1    991   ..........................................................................................  1080
WP_001556799.1  991   ..........................................................................................  1080
HBC7568876.1    991   ..........................................................................................  1080
HAH4707744.1    991   .....................................................S....................................  1080
WP_021525200.1  991   ..........................................................................................  1080
HBA1304970.1    991   ..........................................................................................  1080
WP_021545254.1  991   ..........................................................................................  1080
WP_113341376.1  991   ..........................................................................................  1080
HAX8501378.1    991   ..........................................................................................  1080
HBC8781562.1    991   ..........................................................................................  1080
WP_049074636.1  991   ..........................................................................................  1080
WP_023150719.1  991   ..........................................................................................  1080
EYE09558.1      991   ...T......................................................................................  1080
WP_021521406.1  991   ..........................................................................................  1080
MBB0498836.1    991   ..........................................................................................  1080
HAP0967861.1    991   ..........................................................................................  1080
WP_149846178.1  991   ..........................................................................................  1080
WP_060667427.1  991   ..........................................................................................  1080
HBI9439922.1    991   ..........................................................................................  1080
HBI7936509.1    991   ..........................................................................................  1080
WP_225394784.1  991   ..........................................................................................  1080
EHC2729660.1    991   ..........................................................................................  1080
HAI5392434.1    991   ..........................................................................................  1080
HAL3252735.1    991   ..........................................................................................  1080
```

```
WP_139837327.1  991   ..........................................................................................  1080
WP_053901632.1  991   ..........................................................................................  1080
HAI3750294.1    991   ..........................................................................................  1080
WP_113956116.1  991   ..........................................................................................  1080
HAY5190267.1    991   ..........................................................................................  1080
MBS8788038.1    991   ..........................................................................................  1080
WP_064232224.1  991   ..........................................................................................  1080
WP_118909407.1  991   ..........................................................................................  1080
WP_115766409.1  991   ..........................................................................................  1080
WP_001611046.1  991   ..........................................................................................  1080
HBC4963495.1    991   ..........................................................................................  1080
EFH8304360.1    991   ..........................................................................................  1080
HAN2555901.1    991   ..........................................................................................  1080
MBI9242867.1    991   ..........................................................................................  1080
HAM4370941.1    991   ..........................................................................................  1080
WP_059319927.1  991   ..........................................................................................  1080
WP_113495884.1  991   ..........................................................................................  1080
WP_116991798.1  991   ..........................................................................................  1080
WP_140402642.1  991   ..........................................................................................  1080
WP_169783511.1  991   ..........................................................................................  1080
EEY5188439.1    991   ..........................................................................................  1080
HAG7649195.1    991   ..........................................................................................  1080
HBB1193796.1    991   ..........................................................................................  1080
WP_181203932.1  991   ..........................................................................................  1080
HAH6736077.1    991   ..........................................................................................  1080
HAN3395805.1    990   ..........................................................................................  1079
HAO7397004.1    991   ..........................................................................................  1080
HAI3867277.1    991   ..........................................................................................  1080
HBA4320359.1    991   ..........................................................................................  1080
HAG9275443.1    991   ..........................................................................................  1080
HAX0142153.1    991   ..........................................................................................  1080
WP_155951915.1  991   ..........................................................................................  1080
WP_225390149.1  991   ..........................................................................................  1080
WP_217822457.1  991   ..........................................................................................  1080
EFP7778397.1    991   ..........................................................................................  1080
HAM6401014.1    991   ..........................................................................................  1080
WP_094316666.1  991   ..........................................................................................  1080
MBB0854235.1    991   ..........................................................................................  1080
NPP26331.1      991   ..........................................................................................  1080
WP_199351549.1  991   ..........................................................................................  1080
HBA1072022.1    991   ..........................................................................................  1080
OSP32553.1      995   ..........................................................................................  1084
HBD2267896.1    991   ..........................................................................................  1080
HAX7888640.1    991   ..........................................................................................  1080
HAL3865995.1    991   ..........................................................................................  1080
HBA4313454.1    991   ..........................................................................................  1080
WP_048228344.1  991   ..........................................................................................  1080
HBM2060318.1    991   ..........................................................................................  1080
HAJ3750798.1    991   ..........................................................................................  1080
HBC6041500.1    991   ..........................................................................................  1080
MSI96964.1      991   ..........................................................................................  1080
MBI9784956.1    991   ............................T.............................................................  1080
WP_181199831.1  991   ..........................................................................................  1080
HBE4050048.1    991   ........................V.................................................................  1080
HBK1370968.1    991   ..........................................................................................  1080
```

Query range 13: 1081 to 1170

```
Query           1081  YTDSHAGSDAFSGETKSVGAGLYASAMFESGAYIDLIGKFVHHDNEYTATFAGLGTRDYSSHSWYAGAEVGYRYHVTDSAWIEPQAELVY  1170
WP_001034082.1  1081  ..........................................................................................  1170
HAI6125783.1    1081  ..........................................................................................  1170
WP_021579527.1  1081  .......................................Y..................................................  1170
WP_142457960.1  1081  ..........................................................................................  1170
EFK6366304.1    1081  ..........................................................................................  1170
WP_114145544.1  1081  ..........................................................................................  1170
HBJ6073103.1    1081  ..........................................................................................  1170
WP_126896457.1  1081  .......................................Y..................................................  1170
HAJ1961408.1    1081  .......................................Y..................................................  1170
WP_001034083.1  1081  .......................................Y..................................................  1170
WP_001034084.1  1081  ..........................................................................................  1170
AER85851.1      1085  .......................................Y..................................................  1174
WP_033864944.1  1081  .......................................Y..................................................  1170
EGD9592729.1    1081  .......................................Y..................................................  1170
HAL2735930.1    1081  ..........................................................................................  1170
WP_167580541.1  1081  ..........................................................................................  1170
WP_128430364.1  1081  ..........................................................................................  1170
HAL2502625.1    1081  .......................................Y..................................................  1170
EHI3940979.1    1081  ..........................................................................................  1170
WP_219054700.1  1081  .......................................Y................................................L.  1170
MBI9726529.1    1081  .......................................Y..................................................  1170
HBB7276045.1    1081  ..........................................................................................  1170
WP_001556799.1  1081  .......................................Y..................................................  1170
HBC7568876.1    1081  .......................................Y..................................................  1170
HAH4707744.1    1081  .......................................Y..................................................  1170
WP_021525200.1  1081  .......................................Y..................................................  1170
HBA1304970.1    1081  .......................................Y..................................................  1170
WP_021545254.1  1081  .......................................Y..................................................  1170
WP_113341376.1  1081  .......................................Y..................................................  1170
HAX8501378.1    1081  .......................................Y..................................................  1170
HBC8781562.1    1081  .......................................Y..................................................  1170
WP_049074636.1  1081  .......................................Y..................................................  1170
WP_023150719.1  1081  .......................................Y........................H.........................  1170
EYE09558.1      1081  .......................................Y..................................................  1170
WP_021521406.1  1081  .......................................Y..................................................  1170
MBB0498836.1    1081  ..........................................................................................  1170
HAP0967861.1    1081  .......................................Y..................................................  1170
WP_149846178.1  1081  .......................................Y..................................................  1170
WP_060667427.1  1081  .......................T...............Y..................................................  1170
HBI9439922.1    1081  ...................T...................Y..................................................  1170
HBI7936509.1    1081  .......................................Y..................................................  1170
WP_225394784.1  1081  .......................................Y..................................................  1170
EHC2729660.1    1081  .....................F.................Y..................................................  1170
HAI5392434.1    1081  .......................................Y..................................................  1170
HAL3252735.1    1081  .......................................Y..................................................  1170
WP_139837327.1  1081  .......................................Y..................................................  1170
WP_053901632.1  1081  .......................................Y..................................................  1170
HAI3750294.1    1081  ..........................................................................................  1170
WP_113956116.1  1081  .......................................Y..................................................  1170
HAY5190267.1    1081  .......................................Y..................................................  1170
MBS8788038.1    1081  .......................................Y..................................................  1170
WP_064232224.1  1081  ..........................................................................................  1170
WP_118909407.1  1081  .......................................Y..................................................  1170
WP_115766409.1  1081  .......................................Y..................................................  1170
WP_001611046.1  1081  .......................................Y........T.........................................  1170
HBC4963495.1    1081  .......................................Y..................................................  1170
EFH8304360.1    1081  .......................................Y..................................................  1170
HAN2555901.1    1081  .......................................Y..................................................  1170
MBI9242867.1    1081  .......................................Y..................................................  1170
HAM4370941.1    1081  ..........................................................................................  1170
WP_059319927.1  1081  .......................................Y..................................................  1170
WP_113495884.1  1081  .......................................Y..................................................  1170
WP_116991798.1  1081  .......................................Y..................................................  1170
WP_140402642.1  1081  .......................................Y..................................................  1170
WP_169783511.1  1081  .......................................Y..................................................  1170
EEY5188439.1    1081  .......................................Y..................................................  1170
HAG7649195.1    1081  .......................................Y..................................................  1170
HBB1193796.1    1081  .......................................Y..................................................  1170
WP_181203932.1  1081  .......................................Y..................................................  1170
```

```
HAH6736077.1    1081  .......................................Y...............N..................................  1170
HAN3395805.1    1080  .......................................Y..................................................  1169
HAO7397004.1    1081  .......................................Y..................................................  1170
HAI3867277.1    1081  .......................................Y..................................................  1170
HBA4320359.1    1081  ..........................................................................................  1170
HAG9275443.1    1081  .......................................Y..................................................  1170
HAX0142153.1    1081  .......................................Y..................................................  1170
WP_155951915.1  1081  .......................................Y..................................................  1170
WP_225390149.1  1081  ..........................................................................................  1170
WP_217822457.1  1081  .......................................Y..................................................  1170
EFP7778397.1    1081  .......................................Y..................................................  1170
HAM6401014.1    1081  .......................................Y..................................................  1170
WP_094316666.1  1081  .......................................Y..................................................  1170
MBB0854235.1    1081  .......................................Y..................................................  1170
NPP26331.1      1081  .......................................Y..................................................  1170
WP_199351549.1  1081  .......................................Y..................................................  1170
HBA1072022.1    1081  .......................................Y..................................................  1170
OSP32553.1      1085  ..........................................................................................  1174
HBD2267896.1    1081  .......................................Y..................................................  1170
HAX7888640.1    1081  .......................................Y..................................................  1170
HAL3865995.1    1081  .......................................Y..................................................  1170
HBA4313454.1    1081  .......................................Y..................................................  1170
WP_048228344.1  1081  .......................................Y..................................................  1170
HBM2060318.1    1081  .......................................Y..................................................  1170
HAJ3750798.1    1081  .......................................Y..................................................  1170
HBC6041500.1    1081  .......................................Y..................................................  1170
MSI96964.1      1081  ...............T.......................Y..................................................  1170
MBI9784956.1    1081  .......................................Y..................................................  1170
WP_181199831.1  1081  .......................................Y..................................................  1170
HBE4050048.1    1081  .......................................Y..................................................  1170
HBK1370968.1    1081  .......................................Y..................................................  1170
```

Query range 14: 1171 to 1260

```
Query           1171  GAVSGKQFSWKDQGMNLTMKDKDFNPLIGRTGVDVGKSFSGKDWKVTARAGLGYQFDLFANGETVLRDASGEKRIKGEKDGRMLMNVGLN  1260
WP_001034082.1  1171  ..........................................................................................  1260
HAI6125783.1    1171  ..........................................................................................  1260
WP_021579527.1  1171  ..........................................................................................  1260
WP_142457960.1  1171  ..........................................................................................  1260
EFK6366304.1    1171  ..........................................................................................  1260
WP_114145544.1  1171  ..........................................................................................  1260
HBJ6073103.1    1171  ..........................................................................................  1260
WP_126896457.1  1171  ..........................................................................................  1260
HAJ1961408.1    1171  ..........................................................................................  1260
WP_001034083.1  1171  ..........................................................................................  1260
WP_001034084.1  1171  ..........................................................................................  1260
AER85851.1      1175  ..........................................................................................  1264
WP_033864944.1  1171  ..........................................................................................  1260
EGD9592729.1    1171  ..........................................................................................  1260
HAL2735930.1    1171  ..........................................................................................  1260
WP_167580541.1  1171  ..........................................................................................  1260
WP_128430364.1  1171  ..........................................................................................  1260
HAL2502625.1    1171  ..........................................................................................  1260
EHI3940979.1    1171  ..........................................................................................  1260
WP_219054700.1  1171  ..........................................................................................  1260
MBI9726529.1    1171  ..........................................................................................  1260
HBB7276045.1    1171  ..........................................................................................  1260
WP_001556799.1  1171  ..............................S...........................................................  1260
HBC7568876.1    1171  ..........................................................................................  1260
HAH4707744.1    1171  ..........................................................................................  1260
WP_021525200.1  1171  ..........................................................................................  1260
HBA1304970.1    1171  ..........................................................................................  1260
WP_021545254.1  1171  ..........................................................................................  1260
WP_113341376.1  1171  ..........................................................................................  1260
HAX8501378.1    1171  ..........................................................................................  1260
HBC8781562.1    1171  ..........................................................................................  1260
WP_049074636.1  1171  ..........................................................................................  1260
WP_023150719.1  1171  ..........................................................................................  1260
EYE09558.1      1171  ..........................................................................................  1260
WP_021521406.1  1171  ..........................................................................................  1260
MBB0498836.1    1171  ..........................................................................................  1260
HAP0967861.1    1171  ..........................................................................................  1260
WP_149846178.1  1171  ..........................................................................................  1260
WP_060667427.1  1171  ..........................................................................................  1260
HBI9439922.1    1171  ..........................................................................................  1260
HBI7936509.1    1171  ..........................................................................................  1260
WP_225394784.1  1171  ..........................................................................................  1260
EHC2729660.1    1171  ..........................................................................................  1260
HAI5392434.1    1171  ...........................................................T..............................  1260
HAL3252735.1    1171  ..........................................................................................  1260
WP_139837327.1  1171  ........................................................................................F.  1260
WP_053901632.1  1171  ..........................................................................................  1260
HAI3750294.1    1171  ..........................................................................................  1260
WP_113956116.1  1171  ..........................................................................................  1260
HAY5190267.1    1171  ..........................................................................................  1260
MBS8788038.1    1171  ..........................................................................................  1260
WP_064232224.1  1171  ..........................................................................................  1260
WP_118909407.1  1171  ..........................................................................................  1260
WP_115766409.1  1171  ..........................................................................................  1260
WP_001611046.1  1171  ..........................................................................................  1260
HBC4963495.1    1171  ..........................................................................................  1260
EFH8304360.1    1171  ..........................................................................................  1260
HAN2555901.1    1171  ..........................................................................................  1260
MBI9242867.1    1171  ..........................................................................................  1260
HAM4370941.1    1171  ..........................................................................................  1260
WP_059319927.1  1171  ..........................................................................................  1260
WP_113495884.1  1171  ..........................................................................................  1260
WP_116991798.1  1171  ..........................................................................................  1260
WP_140402642.1  1171  ..........................................................................................  1260
WP_169783511.1  1171  ..........................................................................................  1260
EEY5188439.1    1171  ..........................................................................................  1260
HAG7649195.1    1171  ..........................................................................................  1260
HBB1193796.1    1171  ..........................................................................................  1260
WP_181203932.1  1171  ..........................................................................................  1260
HAH6736077.1    1171  ..........................................................................................  1260
HAN3395805.1    1170  ..........................................................................................  1259
HAO7397004.1    1171  ..........................................................................................  1260
HAI3867277.1    1171  ..........................................................................................  1260
HBA4320359.1    1171  ..........................................................................................  1260
HAG9275443.1    1171  ..........................................................................................  1260
HAX0142153.1    1171  ..........................................................................................  1260
WP_155951915.1  1171  ..........................................................................................  1260
WP_225390149.1  1171  ..........................................................................................  1260
WP_217822457.1  1171  ..........................................................................................  1260
EFP7778397.1    1171  ..........................................................................................  1260
HAM6401014.1    1171  ..........................................................................................  1260
WP_094316666.1  1171  A.........................................................................................  1260
MBB0854235.1    1171  ..........................................................................................  1260
NPP26331.1      1171  ..........................................................................................  1260
WP_199351549.1  1171  ..........................................................................................  1260
HBA1072022.1    1171  ..........................................................................................  1260
OSP32553.1      1175  ..........................................................................................  1264
HBD2267896.1    1171  ..........................................................................T...............  1260
HAX7888640.1    1171  ..................................................S.......................................  1260
HAL3865995.1    1171  ..........................................................................................  1260
HBA4313454.1    1171  ..........................................................................................  1260
WP_048228344.1  1171  ..........................................................................................  1260
HBM2060318.1    1171  ..........................................................................................  1260
```

```
HAJ3750798.1    1171  ..........................................................................................  1260
HBC6041500.1    1171  ..........................................................................................  1260
MSI96964.1      1171  ..........................................................................................  1260
MBI9784956.1    1171  ..........................................................................................  1260
WP_181199831.1  1171  ..........................................................................................  1260
HBE4050048.1    1171  ..........................................................................................  1260
HBK1370968.1    1171  ..........................................................................................  1260
```

Query range 15: 1261 to 1295

```
Query           1261  AEIRDNLRFGLEFEKSAFGKYNVDNAINANFRYSF  1295
WP_001034082.1  1261  ...................................  1295
HAI6125783.1    1261  ...................................  1295
WP_021579527.1  1261  ...................................  1295
WP_142457960.1  1261  ...................................  1295
EFK6366304.1    1261  ...................................  1295
WP_114145544.1  1261  ...................................  1295
HBJ6073103.1    1261  ...................................  1295
WP_126896457.1  1261  ...................................  1295
HAJ1961408.1    1261  ...................................  1295
WP_001034083.1  1261  ...................................  1295
WP_001034084.1  1261  ...................................  1295
AER85851.1      1265  ...................................  1299
WP_033864944.1  1261  ...................................  1295
EGD9592729.1    1261  ...................................  1295
HAL2735930.1    1261  ...................................  1295
WP_167580541.1  1261  ...................................  1295
WP_128430364.1  1261  ...................................  1295
HAL2502625.1    1261  ...................................  1295
EHI3940979.1    1261  ...................................  1295
WP_219054700.1  1261  ...................................  1295
MBI9726529.1    1261  ...................................  1295
HBB7276045.1    1261  ...................................  1295
WP_001556799.1  1261  ...................................  1295
HBC7568876.1    1261  ...................................  1295
HAH4707744.1    1261  ...................................  1295
WP_021525200.1  1261  ...................................  1295
HBA1304970.1    1261  ...................................  1295
WP_021545254.1  1261  ...................................  1295
WP_113341376.1  1261  ...................................  1295
HAX8501378.1    1261  ...................................  1295
HBC8781562.1    1261  ...................................  1295
WP_049074636.1  1261  ...................................  1295
WP_023150719.1  1261  ...................................  1295
EYE09558.1      1261  ...................................  1295
WP_021521406.1  1261  ...................................  1295
MBB0498836.1    1261  ...................................  1295
HAP0967861.1    1261  ...................................  1295
WP_149846178.1  1261  ...................................  1295
WP_060667427.1  1261  ...................................  1295
HBI9439922.1    1261  ...................................  1295
HBI7936509.1    1261  ...................................  1295
WP_225394784.1  1261  ...................................  1295
EHC2729660.1    1261  ...................................  1295
HAI5392434.1    1261  ...................................  1295
HAL3252735.1    1261  ...................................  1295
WP_139837327.1  1261  ...................................  1295
WP_053901632.1  1261  ...................................  1295
HAI3750294.1    1261  ...................................  1295
WP_113956116.1  1261  ...................................  1295
HAY5190267.1    1261  ...................................  1295
MBS8788038.1    1261  ...................................  1295
WP_064232224.1  1261  ...................................  1295
WP_118909407.1  1261  ...................................  1295
WP_115766409.1  1261  ...................................  1295
WP_001611046.1  1261  ...................................  1295
HBC4963495.1    1261  ...................................  1295
EFH8304360.1    1261  ...................................  1295
HAN2555901.1    1261  ...................................  1295
MBI9242867.1    1261  ...................................  1295
HAM4370941.1    1261  ...................................  1295
WP_059319927.1  1261  ...................................  1295
WP_113495884.1  1261  ...................................  1295
WP_116991798.1  1261  ...................................  1295
WP_140402642.1  1261  ..................................   1294
WP_169783511.1  1261  ...................................  1295
EEY5188439.1    1261  ...................................  1295
HAG7649195.1    1261  ...................................  1295
HBB1193796.1    1261  ...................................  1295
WP_181203932.1  1261  ...................................  1295
HAH6736077.1    1261  ...................................  1295
HAN3395805.1    1260  ...................................  1294
HAO7397004.1    1261  ...................................  1295
HAI3867277.1    1261  ...................................  1295
HBA4320359.1    1261  ...................................  1295
HAG9275443.1    1261  ...................................  1295
HAX0142153.1    1261  ...................................  1295
WP_155951915.1  1261  ...................................  1295
WP_225390149.1  1261  ...................................  1295
WP_217822457.1  1261  ...................................  1295
EFP7778397.1    1261  ...................................  1295
HAM6401014.1    1261  ...................................  1295
WP_094316666.1  1261  ...................................  1295
MBB0854235.1    1261  ...................................  1295
NPP26331.1      1261  ...................................  1295
WP_199351549.1  1261  ...................................  1295
HBA1072022.1    1261  ...................................  1295
OSP32553.1      1265  ...................................  1299
HBD2267896.1    1261  ...................................  1295
HAX7888640.1    1261  ...................................  1295
HAL3865995.1    1261  ...................................  1295
HBA4313454.1    1261  ...................................  1295
WP_048228344.1  1261  ...................................  1295
HBM2060318.1    1261  ...................................  1295
HAJ3750798.1    1261  ...................................  1295
HBC6041500.1    1261  ...................................  1295
MSI96964.1      1261  ...................................  1295
MBI9784956.1    1261  ...................................  1295
WP_181199831.1  1261  ...................................  1295
HBE4050048.1    1261  ...................................  1295
HBK1370968.1    1261  ...................................  1295
```

Taxonomy

Reports

- **Lineage**

Organism	Blast Name	Score	Number of Hits	Description
Enterobacterales	enterobacteria		292	
.Enterobacteriaceae	enterobacteria		2	

..Escherichia	enterobacteria		277	
...Escherichia coli	enterobacteria	2631	197	Escherichia coli hits
...Escherichia coli O1	enterobacteria	2631	1	Escherichia coli O1 hits
...Escherichia coli UMEA 3899-1	enterobacteria	2629	1	Escherichia coli UMEA 3899-1 hits
...Escherichia coli O39:H4	enterobacteria	2629	1	Escherichia coli O39:H4 hits
...Escherichia coli O157:H7	enterobacteria	2629	1	Escherichia coli O157:H7 hits
...Escherichia coli O25	enterobacteria	2629	1	Escherichia coli O25 hits
...Escherichia coli IS35	enterobacteria	2629	1	Escherichia coli IS35 hits
...Escherichia coli UMEA 3252-1	enterobacteria	2629	1	Escherichia coli UMEA 3252-1 hits
...Escherichia coli HVH 93 (4-5851025)	enterobacteria	2629	1	Escherichia coli HVH 93 (4-5851025) hits
...Escherichia coli KN1604	enterobacteria	2629	1	Escherichia coli KN1604 hits
...Escherichia coli MH5800	enterobacteria	2629	1	Escherichia coli MH5800 hits
...Escherichia coli JJ2657	enterobacteria	2629	1	Escherichia coli JJ2657 hits
...Escherichia coli JJ2608	enterobacteria	2629	1	Escherichia coli JJ2608 hits
...Escherichia coli JJ2555	enterobacteria	2629	1	Escherichia coli JJ2555 hits
...Escherichia coli JJ2550	enterobacteria	2629	1	Escherichia coli JJ2550 hits
...Escherichia coli JJ2578	enterobacteria	2629	1	Escherichia coli JJ2578 hits
...Escherichia coli JJ2528	enterobacteria	2629	1	Escherichia coli JJ2528 hits
...Escherichia coli JJ2508	enterobacteria	2629	1	Escherichia coli JJ2508 hits
...Escherichia coli JJ2444	enterobacteria	2629	1	Escherichia coli JJ2444 hits
...Escherichia coli ZH063	enterobacteria	2629	1	Escherichia coli ZH063 hits
...Escherichia coli ZH071	enterobacteria	2629	1	Escherichia coli ZH071 hits
...Escherichia coli CD340	enterobacteria	2629	1	Escherichia coli CD340 hits
...Escherichia coli ZH164	enterobacteria	2629	1	Escherichia coli ZH164 hits
...Escherichia coli U024	enterobacteria	2629	1	Escherichia coli U024 hits
...Escherichia coli U004	enterobacteria	2629	1	Escherichia coli U004 hits
...Escherichia coli SaT158	enterobacteria	2629	1	Escherichia coli SaT158 hits
...Escherichia coli SaT040	enterobacteria	2629	1	Escherichia coli SaT040 hits
...Escherichia coli SaT049	enterobacteria	2629	1	Escherichia coli SaT049 hits
...Escherichia coli MVAST179	enterobacteria	2629	1	Escherichia coli MVAST179 hits
...Escherichia coli MVAST131	enterobacteria	2629	1	Escherichia coli MVAST131 hits
...Escherichia coli JJ2038	enterobacteria	2629	1	Escherichia coli JJ2038 hits
...Escherichia coli JJ2183	enterobacteria	2629	1	Escherichia coli JJ2183 hits
...Escherichia coli MVAST084	enterobacteria	2629	1	Escherichia coli MVAST084 hits
...Escherichia coli MVAST077	enterobacteria	2629	1	Escherichia coli MVAST077 hits
...Escherichia coli JJ1908	enterobacteria	2629	1	Escherichia coli JJ1908 hits
...Escherichia coli JJ2050	enterobacteria	2629	1	Escherichia coli JJ2050 hits
...Escherichia coli JJ2193	enterobacteria	2629	1	Escherichia coli JJ2193 hits
...Escherichia coli JJ2643	enterobacteria	2629	1	Escherichia coli JJ2643 hits
...Escherichia coli JJ2016	enterobacteria	2629	1	Escherichia coli JJ2016 hits
...Escherichia coli MVAST038	enterobacteria	2629	1	Escherichia coli MVAST038 hits
...Escherichia coli MVAST046	enterobacteria	2629	1	Escherichia coli MVAST046 hits
...Escherichia coli H061	enterobacteria	2629	1	Escherichia coli H061 hits
...Escherichia coli MVAST014	enterobacteria	2629	1	Escherichia coli MVAST014 hits
...Escherichia coli MVAST0036	enterobacteria	2629	1	Escherichia coli MVAST0036 hits
...Escherichia coli JJ1897	enterobacteria	2629	1	Escherichia coli JJ1897 hits
...Escherichia coli G213	enterobacteria	2629	1	Escherichia coli G213 hits
...Escherichia coli G132	enterobacteria	2629	1	Escherichia coli G132 hits
...Escherichia coli JJ1901	enterobacteria	2629	1	Escherichia coli JJ1901 hits
...Escherichia coli H016	enterobacteria	2629	1	Escherichia coli H016 hits
...Escherichia coli G150	enterobacteria	2629	1	Escherichia coli G150 hits
...Escherichia coli CU758	enterobacteria	2629	1	Escherichia coli CU758 hits
...Escherichia coli H003	enterobacteria	2629	1	Escherichia coli H003 hits
...Escherichia coli H006	enterobacteria	2629	1	Escherichia coli H006 hits
...Escherichia coli CD400	enterobacteria	2629	1	Escherichia coli CD400 hits
...Escherichia coli C001	enterobacteria	2629	1	Escherichia coli C001 hits
...Escherichia coli JJ2118	enterobacteria	2629	1	Escherichia coli JJ2118 hits
...Escherichia coli JJ2134	enterobacteria	2629	1	Escherichia coli JJ2134 hits
...Escherichia coli JJ2547	enterobacteria	2629	1	Escherichia coli JJ2547 hits
...Escherichia coli O157:H7 str. EC10	enterobacteria	2628	1	Escherichia coli O157:H7 str. EC10 hits
...Escherichia coli VR50	enterobacteria	2628	1	Escherichia coli VR50 hits
...Escherichia coli str. 'clone D i2'	enterobacteria	2628	1	Escherichia coli str. 'clone D i2' hits
...Escherichia coli str. 'clone D i14'	enterobacteria	2628	1	Escherichia coli str. 'clone D i14' hits
...Escherichia coli O25b:H4	enterobacteria	2628	1	Escherichia coli O25b:H4 hits
...Escherichia coli LAU-EC7	enterobacteria	2628	1	Escherichia coli LAU-EC7 hits

...Escherichia coli 1-250-04_S4_C2	enterobacteria	2628	1	Escherichia coli 1-250-04_S4_C2 hits
...Escherichia coli 1-250-04_S4_C1	enterobacteria	2628	1	Escherichia coli 1-250-04_S4_C1 hits
...Escherichia coli KTE57	enterobacteria	2628	1	Escherichia coli KTE57 hits
...Escherichia coli HVH 77 (4-2605759)	enterobacteria	2628	1	Escherichia coli HVH 77 (4-2605759) hits
...Escherichia coli HVH 216 (4-3042952)	enterobacteria	2627	1	Escherichia coli HVH 216 (4-3042952) hits
...Escherichia coli 910096-2	enterobacteria	2627	1	Escherichia coli 910096-2 hits
...Escherichia coli 1-110-08_S4_C1	enterobacteria	2627	1	Escherichia coli 1-110-08_S4_C1 hits
...Escherichia coli HVH 51 (4-2172526)	enterobacteria	2627	1	Escherichia coli HVH 51 (4-2172526) hits
...Escherichia coli O21	enterobacteria	2627	1	Escherichia coli O21 hits
...Escherichia coli G199	enterobacteria	2627	1	Escherichia coli G199 hits
...Escherichia coli O25b:H4-ST131	enterobacteria	2627	6	Escherichia coli O25b:H4-ST131 hits
...Escherichia coli MVAST158	enterobacteria	2627	1	Escherichia coli MVAST158 hits
..Shigella flexneri	enterobacteria	2631	7	Shigella flexneri hits
..Shigella boydii	enterobacteria	2631	2	Shigella boydii hits
..Shigella sp. FC2045	enterobacteria	2631	1	Shigella sp. FC2045 hits
..Shigella sp. FC2928	enterobacteria	2631	1	Shigella sp. FC2928 hits
..Shigella sonnei	enterobacteria	2629	2	Shigella sonnei hits
.Enterobacteriaceae	enterobacteria	2629	2	Enterobacteriaceae hits

- **Organism**

Description	Score	E value	Accession
Escherichia coli [enterobacteria]			
serine protease autotransporter toxin Sat [Escherichia coli]	2631	0.0	WP_001034082
serine protease autotransporter toxin Sat [Escherichia coli]	2630	0.0	HAI6125783
serine protease autotransporter toxin Sat [Escherichia coli]	2629	0.0	WP_021579527
serine protease autotransporter toxin Sat [Escherichia coli]	2629	0.0	WP_142457960
serine protease autotransporter toxin Sat [Escherichia coli]	2629	0.0	HAH7070639
serine protease autotransporter toxin Sat [Escherichia coli]	2629	0.0	HAI5503062
serine protease autotransporter toxin Sat [Escherichia coli]	2629	0.0	HBC8693275
serine protease autotransporter toxin Sat [Escherichia coli]	2629	0.0	EFK6366304
serine protease autotransporter toxin Sat [Escherichia coli]	2629	0.0	WP_114145544
autotransporter outer membrane beta-barrel domain-containing protein [Escherichia coli]	2629	0.0	OOI47530
autotransporter outer membrane beta-barrel domain-containing protein [Escherichia coli]	2629	0.0	OOJ42315
serine protease autotransporter toxin Sat [Escherichia coli]	2629	0.0	HBJ6073103
serine protease autotransporter toxin Sat [Escherichia coli]	2629	0.0	EGO3808398
serine protease autotransporter toxin Sat [Escherichia coli]	2629	0.0	MBI9649871
serine protease autotransporter toxin Sat [Escherichia coli]	2629	0.0	MBW9499880
serine protease autotransporter toxin Sat [Escherichia coli]	2629	0.0	MBW9539912
serine protease autotransporter toxin Sat [Escherichia coli]	2629	0.0	MBW9598623
serine protease autotransporter toxin Sat [Escherichia coli]	2629	0.0	HAJ1961408
serine protease autotransporter toxin Sat [Escherichia coli]	2628	0.0	WP_001034084
serine protease autotransporter toxin Sat [Escherichia coli]	2628	0.0	EAB6864467
serine protease autotransporter toxin Sat [Escherichia coli]	2628	0.0	EEU2827586
serine protease autotransporter toxin Sat [Escherichia coli]	2628	0.0	EFA4390733
autotransporter outer membrane beta-barrel domain-containing protein [Escherichia coli]	2628	0.0	AQV56180
serine protease autotransporter toxin Sat [Escherichia coli]	2628	0.0	WP_033864944
serine protease autotransporter toxin Sat [Escherichia coli]	2628	0.0	EGD9592729
serine protease autotransporter toxin Sat [Escherichia coli]	2628	0.0	HAL2735930
serine protease autotransporter toxin Sat [Escherichia coli]	2628	0.0	WP_167580541
serine protease autotransporter toxin Sat [Escherichia coli]	2628	0.0	NJB54887
serine protease autotransporter toxin Sat [Escherichia coli]	2628	0.0	WP_128430364
serine protease autotransporter toxin Sat [Escherichia coli]	2628	0.0	EFA4555807
serine protease autotransporter toxin Sat [Escherichia coli]	2628	0.0	EFB2292560
serine protease autotransporter toxin Sat [Escherichia coli]	2628	0.0	EFK2831551
serine protease autotransporter toxin Sat [Escherichia coli]	2628	0.0	EGD8224732
serine protease autotransporter toxin Sat [Escherichia coli]	2628	0.0	EGE0307816
serine protease autotransporter toxin Sat [Escherichia coli]	2628	0.0	HAL2502625
autotransporter outer membrane beta-barrel domain-containing protein [Escherichia coli]	2628	0.0	WP_219054700
serine protease autotransporter toxin Sat [Escherichia coli]	2628	0.0	MBW2806267
serine protease autotransporter toxin Sat [Escherichia coli]	2628	0.0	MBI9726529
serine protease autotransporter toxin Sat [Escherichia coli]	2628	0.0	HAI9203572
serine protease autotransporter toxin Sat [Escherichia coli]	2628	0.0	HBB7276045
serine protease autotransporter toxin Sat [Escherichia coli]	2628	0.0	WP_001556799

Description	Score	E value	Accession
serine protease autotransporter toxin Sat [Escherichia coli]	2628	0.0	HAI3051513
serine protease autotransporter toxin Sat [Escherichia coli]	2628	0.0	HAI4879870
serine protease autotransporter toxin Sat [Escherichia coli]	2628	0.0	HAY4619476
serine protease autotransporter toxin Sat [Escherichia coli]	2628	0.0	HAY5587971
serine protease autotransporter toxin Sat [Escherichia coli]	2628	0.0	HBC7568876
serine protease autotransporter toxin Sat [Escherichia coli]	2628	0.0	HAH4707744
serine protease autotransporter toxin Sat [Escherichia coli]	2628	0.0	WP_021525200
serine protease autotransporter toxin Sat [Escherichia coli]	2628	0.0	HBA1304970
serine protease autotransporter toxin Sat [Escherichia coli]	2627	0.0	WP_021545254
serine protease autotransporter toxin Sat [Escherichia coli]	2627	0.0	WP_113341376
serine protease autotransporter toxin Sat [Escherichia coli]	2627	0.0	EFN2062961
serine protease autotransporter toxin Sat [Escherichia coli]	2627	0.0	MQR32067
serine protease autotransporter toxin Sat [Escherichia coli]	2627	0.0	RRN06535
serine protease autotransporter toxin Sat [Escherichia coli]	2627	0.0	HAH0224241
serine protease autotransporter toxin Sat [Escherichia coli]	2627	0.0	HAH4727320
serine protease autotransporter toxin Sat [Escherichia coli]	2627	0.0	HAX8501378
serine protease autotransporter toxin Sat [Escherichia coli]	2627	0.0	HBC8781562
serine protease autotransporter toxin Sat [Escherichia coli]	2627	0.0	WP_049074636
serine protease autotransporter toxin Sat [Escherichia coli]	2627	0.0	WP_023150719
serine protease autotransporter toxin Sat [Escherichia coli]	2627	0.0	WP_021521406
serine protease autotransporter toxin Sat [Escherichia coli]	2627	0.0	KAA2076204
serine protease autotransporter toxin Sat [Escherichia coli]	2627	0.0	KAA2105643
serine protease autotransporter toxin Sat [Escherichia coli]	2627	0.0	KAA2126002
serine protease autotransporter toxin Sat [Escherichia coli]	2627	0.0	MBB0983140
serine protease autotransporter toxin Sat [Escherichia coli]	2627	0.0	MBB0498836
serine protease autotransporter toxin Sat [Escherichia coli]	2627	0.0	HAP0967861
serine protease autotransporter toxin Sat [Escherichia coli]	2627	0.0	HAX6362869
serine protease autotransporter toxin Sat [Escherichia coli]	2627	0.0	HAX6372935
serine protease autotransporter toxin Sat [Escherichia coli]	2627	0.0	HAX6427584
serine protease autotransporter toxin Sat [Escherichia coli]	2627	0.0	WP_149846178
serine protease autotransporter toxin Sat [Escherichia coli]	2627	0.0	EHR8537703
serine protease autotransporter toxin Sat [Escherichia coli]	2627	0.0	KAA2152962
serine protease autotransporter toxin Sat [Escherichia coli]	2627	0.0	HAK9473061
serine protease autotransporter toxin Sat [Escherichia coli]	2627	0.0	WP_060667427
autotransporter outer membrane beta-barrel domain-containing protein [Escherichia coli]	2627	0.0	ALT50789
serine protease autotransporter toxin Sat [Escherichia coli]	2627	0.0	HBI9439922
serine protease autotransporter toxin Sat [Escherichia coli]	2627	0.0	HBI7936509
autotransporter outer membrane beta-barrel domain-containing protein [Escherichia coli]	2627	0.0	WP_225394784
serine protease autotransporter toxin Sat [Escherichia coli]	2627	0.0	EFT4571186
serine protease autotransporter toxin Sat [Escherichia coli]	2627	0.0	EHC2729660
serine protease autotransporter toxin Sat [Escherichia coli]	2627	0.0	HAZ4666781
serine protease autotransporter toxin Sat [Escherichia coli]	2627	0.0	HBA1319342
serine protease autotransporter toxin Sat [Escherichia coli]	2627	0.0	HBC7154477
serine protease autotransporter toxin Sat [Escherichia coli]	2627	0.0	HBD2195500
serine protease autotransporter toxin Sat [Escherichia coli]	2627	0.0	HAI5392434
serine protease autotransporter toxin Sat [Escherichia coli]	2627	0.0	HAL3252735
serine protease autotransporter toxin Sat [Escherichia coli]	2627	0.0	HAX5825434
serine protease autotransporter toxin Sat [Escherichia coli]	2627	0.0	HAY0853007
serine protease autotransporter toxin Sat [Escherichia coli]	2627	0.0	HAY2236716
serine protease autotransporter toxin Sat [Escherichia coli]	2627	0.0	HAY3259201
serine protease autotransporter toxin Sat [Escherichia coli]	2627	0.0	WP_139837327
autotransporter outer membrane beta-barrel domain-containing protein [Escherichia coli]	2627	0.0	OSY85009
serine protease autotransporter toxin Sat [Escherichia coli]	2627	0.0	HAL1710508
serine protease autotransporter toxin Sat [Escherichia coli]	2627	0.0	WP_053901632
serine protease autotransporter toxin Sat [Escherichia coli]	2627	0.0	EEV5815857
serine protease autotransporter toxin Sat [Escherichia coli]	2627	0.0	EGG1101312
serine protease autotransporter toxin Sat [Escherichia coli]	2627	0.0	NJY07520
serine protease autotransporter toxin Sat [Escherichia coli]	2627	0.0	TXQ35066
serine protease autotransporter toxin Sat [Escherichia coli]	2627	0.0	HAI3750294
serine protease autotransporter toxin Sat [Escherichia coli]	2627	0.0	WP_113956116
serine protease autotransporter toxin Sat [Escherichia coli]	2627	0.0	HAY5190267
serine protease autotransporter toxin Sat [Escherichia coli]	2627	0.0	MBS8788038
serine protease autotransporter toxin Sat [Escherichia coli]	2627	0.0	WP_064232224

Description	Score	E value	Accession
autotransporter [Escherichia coli]	2627	0.0	OAO63702
serine protease autotransporter toxin Sat [Escherichia coli]	2627	0.0	WP_118909407
serine protease autotransporter toxin Sat [Escherichia coli]	2627	0.0	EGO3591555
serine protease autotransporter toxin Sat [Escherichia coli]	2627	0.0	NBE40572
serine protease autotransporter toxin Sat [Escherichia coli]	2627	0.0	HAJ5426127
serine protease autotransporter toxin Sat [Escherichia coli]	2627	0.0	HBB6117255
serine protease autotransporter toxin Sat [Escherichia coli]	2627	0.0	WP_115766409
serine protease autotransporter toxin Sat [Escherichia coli]	2627	0.0	WP_001611046
serine protease autotransporter toxin Sat [Escherichia coli]	2627	0.0	EFD0420853
serine protease autotransporter toxin Sat [Escherichia coli]	2627	0.0	EFE3323826
serine protease autotransporter toxin Sat [Escherichia coli]	2627	0.0	EFE3353236
serine protease autotransporter toxin Sat [Escherichia coli]	2627	0.0	EFH2673541
serine protease autotransporter toxin Sat [Escherichia coli]	2627	0.0	EFH5306495
serine protease autotransporter toxin Sat [Escherichia coli]	2627	0.0	HBC4963495
serine protease autotransporter toxin Sat [Escherichia coli]	2627	0.0	EFH8304360
serine protease autotransporter toxin Sat [Escherichia coli]	2627	0.0	MBI9242867
serine protease autotransporter toxin Sat [Escherichia coli]	2627	0.0	HAP1192552
serine protease autotransporter toxin Sat [Escherichia coli]	2627	0.0	HAM4370941
serine protease autotransporter toxin Sat [Escherichia coli]	2627	0.0	HAO0429778
serine protease autotransporter toxin Sat [Escherichia coli]	2627	0.0	WP_059319927
autotransporter outer membrane beta-barrel domain-containing protein [Escherichia coli]	2627	0.0	KUX86724
serine protease autotransporter toxin Sat [Escherichia coli]	2627	0.0	WP_113495884
serine protease EspP [Escherichia coli]	2627	0.0	GCN69540
serine protease EspP [Escherichia coli]	2627	0.0	GCT45587
serine protease EspP [Escherichia coli]	2627	0.0	GCY95461
serine protease EspP [Escherichia coli]	2627	0.0	GDB27613
serine protease EspP [Escherichia coli]	2627	0.0	GDC05953
serine protease autotransporter toxin Sat [Escherichia coli]	2627	0.0	WP_116991798
serine protease autotransporter toxin Sat [Escherichia coli]	2627	0.0	MBB0806913
serine protease autotransporter toxin Sat [Escherichia coli]	2627	0.0	MBW2773060
serine protease autotransporter toxin Sat [Escherichia coli]	2627	0.0	MRH27236
serine protease autotransporter toxin Sat [Escherichia coli]	2627	0.0	NPJ79193
serine protease autotransporter toxin Sat, partial [Escherichia coli]	2627	0.0	WP_140402642
serine protease autotransporter toxin Sat [Escherichia coli]	2627	0.0	WP_169783511
serine protease autotransporter toxin Sat [Escherichia coli]	2627	0.0	EEY5188439
serine protease autotransporter toxin Sat [Escherichia coli]	2627	0.0	EFC2077216
serine protease autotransporter toxin Sat [Escherichia coli]	2627	0.0	HAG7649195
serine protease autotransporter toxin Sat [Escherichia coli]	2627	0.0	HBB1193796
serine protease autotransporter toxin Sat [Escherichia coli]	2627	0.0	WP_181203932
serine protease autotransporter toxin Sat [Escherichia coli]	2627	0.0	EIQ6130730
serine protease autotransporter toxin Sat [Escherichia coli]	2627	0.0	EIQ6311371
serine protease autotransporter toxin Sat [Escherichia coli]	2627	0.0	EIQ6352661
serine protease autotransporter toxin Sat [Escherichia coli]	2627	0.0	EIQ6386402
serine protease autotransporter toxin Sat [Escherichia coli]	2627	0.0	EIQ6395996
serine protease autotransporter toxin Sat [Escherichia coli]	2627	0.0	HAH6736077
serine protease autotransporter toxin Sat [Escherichia coli]	2627	0.0	HAN3395805
serine protease autotransporter toxin Sat [Escherichia coli]	2627	0.0	HAO7397004
serine protease autotransporter toxin Sat [Escherichia coli]	2627	0.0	HAI3867277
serine protease autotransporter toxin Sat [Escherichia coli]	2627	0.0	HBA4320359
serine protease autotransporter toxin Sat [Escherichia coli]	2627	0.0	HAG9275443
serine protease autotransporter toxin Sat [Escherichia coli]	2627	0.0	WP_155951915
serine protease autotransporter toxin Sat [Escherichia coli]	2627	0.0	HAZ5813250
autotransporter outer membrane beta-barrel domain-containing protein [Escherichia coli]	2627	0.0	WP_225390149
autotransporter outer membrane beta-barrel domain-containing protein [Escherichia coli]	2627	0.0	WP_217822457
serine protease autotransporter toxin Sat [Escherichia coli]	2627	0.0	MBV5228177
serine protease autotransporter toxin Sat [Escherichia coli]	2627	0.0	MBV5233005
serine protease autotransporter toxin Sat [Escherichia coli]	2627	0.0	HAM6401014
serine protease autotransporter toxin Sat [Escherichia coli]	2627	0.0	WP_094316666
serine protease autotransporter toxin Sat [Escherichia coli]	2627	0.0	EFK3996975
serine protease autotransporter toxin Sat [Escherichia coli]	2627	0.0	EII2859423
serine protease autotransporter toxin Sat [Escherichia coli]	2627	0.0	MBJ7038041
serine protease autotransporter toxin Sat [Escherichia coli]	2627	0.0	MBJ7117004
autotransporter outer membrane beta-barrel domain-containing protein [Escherichia coli]	2627	0.0	OOI11196

Description	Score	E value	Accession
serine protease autotransporter toxin Sat [Escherichia coli]	2627	0.0	MBB0854235
serine protease autotransporter toxin Sat [Escherichia coli]	2627	0.0	NPP26331
autotransporter outer membrane beta-barrel domain-containing protein [Escherichia coli]	2627	0.0	WP_199351549
serine protease autotransporter toxin Sat [Escherichia coli]	2627	0.0	MBJ6819672
serine protease autotransporter toxin Sat [Escherichia coli]	2627	0.0	HBA1072022
autotransporter outer membrane beta-barrel domain-containing protein [Escherichia coli]	2627	0.0	OSP32553
autotransporter outer membrane beta-barrel domain-containing protein [Escherichia coli]	2627	0.0	TFA22384
serine protease autotransporter toxin Sat [Escherichia coli]	2627	0.0	HBD2267896
serine protease autotransporter toxin Sat [Escherichia coli]	2627	0.0	HAX7888640
serine protease autotransporter toxin Sat [Escherichia coli]	2627	0.0	HAL3865995
serine protease autotransporter toxin Sat [Escherichia coli]	2627	0.0	HBA4313454
serine protease autotransporter toxin Sat [Escherichia coli]	2627	0.0	WP_048228344
serine protease autotransporter toxin Sat [Escherichia coli]	2627	0.0	EFC8865612
serine protease autotransporter toxin Sat [Escherichia coli]	2627	0.0	EFK8052655
serine protease autotransporter toxin Sat [Escherichia coli]	2627	0.0	EFK8062124
serine protease sat autotransporter [Escherichia coli]	2627	0.0	KLX21102
serine protease sat autotransporter [Escherichia coli]	2627	0.0	KLX31217
serine protease autotransporter toxin Sat [Escherichia coli]	2627	0.0	HBM2060318
serine protease autotransporter toxin Sat [Escherichia coli]	2627	0.0	HBM2138067
serine protease autotransporter toxin Sat [Escherichia coli]	2627	0.0	HBM2195022
serine protease autotransporter toxin Sat [Escherichia coli]	2627	0.0	HBM2204591
serine protease autotransporter toxin Sat [Escherichia coli]	2627	0.0	HBM2228563
serine protease autotransporter toxin Sat [Escherichia coli]	2627	0.0	HAJ3750798
serine protease autotransporter toxin Sat [Escherichia coli]	2627	0.0	HBC6041500
serine protease autotransporter toxin Sat [Escherichia coli]	2627	0.0	MSI96964
serine protease autotransporter toxin Sat [Escherichia coli]	2627	0.0	MBI9784956
serine protease autotransporter toxin Sat [Escherichia coli]	2627	0.0	WP_181199831
serine protease autotransporter toxin Sat [Escherichia coli]	2627	0.0	MBA1707390
serine protease autotransporter toxin Sat [Escherichia coli]	2627	0.0	HBE4050048
serine protease autotransporter toxin Sat [Escherichia coli]	2627	0.0	HBK1370968
Escherichia coli O1 [enterobacteria]			
serine protease autotransporter toxin Sat [Escherichia coli O1]	2631	0.0	EEZ6060212
Shigella flexneri [enterobacteria]			
serine protease autotransporter toxin Sat [Shigella flexneri]	2631	0.0	EFQ0017957
serine protease autotransporter toxin Sat [Shigella flexneri]	2629	0.0	EFW2042547
serine protease autotransporter toxin Sat [Shigella flexneri]	2627	0.0	EFP7778397
serine protease autotransporter toxin Sat [Shigella flexneri]	2627	0.0	EFX2165787
serine protease autotransporter toxin Sat [Shigella flexneri]	2627	0.0	HAY5193548
serine protease autotransporter toxin Sat [Shigella flexneri]	2627	0.0	HAY5252721
serine protease autotransporter toxin Sat [Shigella flexneri]	2627	0.0	HAY7194005
Shigella boydii [enterobacteria]			
serine protease autotransporter toxin Sat [Shigella boydii]	2631	0.0	EIH0344184
serine protease autotransporter toxin Sat [Shigella boydii]	2627	0.0	EFX6129646
Shigella sp. FC2045 [enterobacteria]			
autotransporter outer membrane beta-barrel domain-containing protein [Shigella sp. FC2045]	2631	0.0	ODG72079
Shigella sp. FC2928 [enterobacteria]			
autotransporter outer membrane beta-barrel domain-containing protein [Shigella sp. FC2928]	2631	0.0	ODG78167
Escherichia coli UMEA 3899-1 [enterobacteria]			
serine protease sat autotransporter [Escherichia coli UMEA 3899-1]	2629	0.0	ERA44834
Enterobacteriaceae [enterobacteria]			
MULTISPECIES: serine protease autotransporter toxin Sat [Enterobacteriaceae]	2629	0.0	WP_126896457
MULTISPECIES: serine protease autotransporter toxin Sat [Enterobacteriaceae]	2629	0.0	WP_001034083
Escherichia coli O39:H4 [enterobacteria]			
serine protease autotransporter toxin Sat [Escherichia coli O39:H4]	2629	0.0	EFA8806175
Escherichia coli O157:H7 [enterobacteria]			
serine protease autotransporter toxin Sat [Escherichia coli O157:H7]	2629	0.0	EFD1460826
Escherichia coli O25 [enterobacteria]			
serine protease autotransporter toxin Sat [Escherichia coli O25]	2629	0.0	EFN8566480
Shigella sonnei [enterobacteria]			
serine protease autotransporter toxin Sat [Shigella sonnei]	2629	0.0	EFZ4857089
serine protease autotransporter toxin Sat [Shigella sonnei]	2628	0.0	EHI3940979
Escherichia coli IS35 [enterobacteria]			
Per-activated serine protease autotransporter enterotoxin EspC [Escherichia coli IS35]	2629	0.0	CDL05232

Description	Score	E value	Accession
Escherichia coli UMEA 3252-1 [enterobacteria]			
serine protease autotransporter toxin Sat [Escherichia coli UMEA 3252-1]	2629	0.0	HAJ6400147
Escherichia coli HVH 93 (4-5851025) [enterobacteria]			
serine protease autotransporter toxin Sat [Escherichia coli HVH 93 (4-5851025)]	2629	0.0	HAJ6409752
Escherichia coli KN1604 [enterobacteria]			
serine protease autotransporter toxin Sat [Escherichia coli KN1604]	2629	0.0	HAX0006506
Escherichia coli MH5800 [enterobacteria]			
serine protease autotransporter toxin Sat [Escherichia coli MH5800]	2629	0.0	HAX0016325
Escherichia coli JJ2657 [enterobacteria]			
serine protease autotransporter toxin Sat [Escherichia coli JJ2657]	2629	0.0	HAX0021232
Escherichia coli JJ2608 [enterobacteria]			
serine protease autotransporter toxin Sat [Escherichia coli JJ2608]	2629	0.0	HAX0025986
Escherichia coli JJ2555 [enterobacteria]			
serine protease autotransporter toxin Sat [Escherichia coli JJ2555]	2629	0.0	HAX0030327
Escherichia coli JJ2550 [enterobacteria]			
serine protease autotransporter toxin Sat [Escherichia coli JJ2550]	2629	0.0	HAX0035654
Escherichia coli JJ2578 [enterobacteria]			
serine protease autotransporter toxin Sat [Escherichia coli JJ2578]	2629	0.0	HAX0040618
Escherichia coli JJ2528 [enterobacteria]			
serine protease autotransporter toxin Sat [Escherichia coli JJ2528]	2629	0.0	HAX0045758
Escherichia coli JJ2508 [enterobacteria]			
serine protease autotransporter toxin Sat [Escherichia coli JJ2508]	2629	0.0	HAX0055499
Escherichia coli JJ2444 [enterobacteria]			
serine protease autotransporter toxin Sat [Escherichia coli JJ2444]	2629	0.0	HAX0060482
Escherichia coli ZH063 [enterobacteria]			
serine protease autotransporter toxin Sat [Escherichia coli ZH063]	2629	0.0	HAX0075156
Escherichia coli ZH071 [enterobacteria]			
serine protease autotransporter toxin Sat [Escherichia coli ZH071]	2629	0.0	HAX0079826
Escherichia coli CD340 [enterobacteria]			
serine protease autotransporter toxin Sat [Escherichia coli CD340]	2629	0.0	HAX0084719
Escherichia coli ZH164 [enterobacteria]			
serine protease autotransporter toxin Sat [Escherichia coli ZH164]	2629	0.0	HAX0089459
Escherichia coli U024 [enterobacteria]			
serine protease autotransporter toxin Sat [Escherichia coli U024]	2629	0.0	HAX0099239
Escherichia coli U004 [enterobacteria]			
serine protease autotransporter toxin Sat [Escherichia coli U004]	2629	0.0	HAX0104929
Escherichia coli SaT158 [enterobacteria]			
serine protease autotransporter toxin Sat [Escherichia coli SaT158]	2629	0.0	HAX0113730
Escherichia coli SaT040 [enterobacteria]			
serine protease autotransporter toxin Sat [Escherichia coli SaT040]	2629	0.0	HAX0127979
Escherichia coli SaT049 [enterobacteria]			
serine protease autotransporter toxin Sat [Escherichia coli SaT049]	2629	0.0	HAX0132629
Escherichia coli MVAST179 [enterobacteria]			
serine protease autotransporter toxin Sat [Escherichia coli MVAST179]	2629	0.0	HAX0137439
Escherichia coli MVAST131 [enterobacteria]			
serine protease autotransporter toxin Sat [Escherichia coli MVAST131]	2629	0.0	HAX0151890
Escherichia coli JJ2038 [enterobacteria]			
serine protease autotransporter toxin Sat [Escherichia coli JJ2038]	2629	0.0	HAX0156531
Escherichia coli JJ2183 [enterobacteria]			
serine protease autotransporter toxin Sat [Escherichia coli JJ2183]	2629	0.0	HAX0165804
Escherichia coli MVAST084 [enterobacteria]			
serine protease autotransporter toxin Sat [Escherichia coli MVAST084]	2629	0.0	HAX0170908
Escherichia coli MVAST077 [enterobacteria]			
serine protease autotransporter toxin Sat [Escherichia coli MVAST077]	2629	0.0	HAX0175195
Escherichia coli JJ1908 [enterobacteria]			
serine protease autotransporter toxin Sat [Escherichia coli JJ1908]	2629	0.0	HAX0180480
Escherichia coli JJ2050 [enterobacteria]			
serine protease autotransporter toxin Sat [Escherichia coli JJ2050]	2629	0.0	HAX0185188
Escherichia coli JJ2193 [enterobacteria]			
serine protease autotransporter toxin Sat [Escherichia coli JJ2193]	2629	0.0	HAX0190116
Escherichia coli JJ2643 [enterobacteria]			
serine protease autotransporter toxin Sat [Escherichia coli JJ2643]	2629	0.0	HAX0195145
Escherichia coli JJ2016 [enterobacteria]			

Description	Score	E value	Accession
serine protease autotransporter toxin Sat [Escherichia coli JJ2016]	2629	0.0	HAX0204896
Escherichia coli MVAST038 [enterobacteria]			
serine protease autotransporter toxin Sat [Escherichia coli MVAST038]	2629	0.0	HAX0209492
Escherichia coli MVAST046 [enterobacteria]			
serine protease autotransporter toxin Sat [Escherichia coli MVAST046]	2629	0.0	HAX0214131
Escherichia coli H061 [enterobacteria]			
serine protease autotransporter toxin Sat [Escherichia coli H061]	2629	0.0	HAX0219297
Escherichia coli MVAST014 [enterobacteria]			
serine protease autotransporter toxin Sat [Escherichia coli MVAST014]	2629	0.0	HAX0224008
Escherichia coli MVAST0036 [enterobacteria]			
serine protease autotransporter toxin Sat [Escherichia coli MVAST0036]	2629	0.0	HAX0238067
Escherichia coli JJ1897 [enterobacteria]			
serine protease autotransporter toxin Sat [Escherichia coli JJ1897]	2629	0.0	HAX0243615
Escherichia coli G213 [enterobacteria]			
serine protease autotransporter toxin Sat [Escherichia coli G213]	2629	0.0	HAX0253346
Escherichia coli G132 [enterobacteria]			
serine protease autotransporter toxin Sat [Escherichia coli G132]	2629	0.0	HAX0258172
Escherichia coli JJ1901 [enterobacteria]			
serine protease autotransporter toxin Sat [Escherichia coli JJ1901]	2629	0.0	HAX0267967
Escherichia coli H016 [enterobacteria]			
serine protease autotransporter toxin Sat [Escherichia coli H016]	2629	0.0	HAX0277630
Escherichia coli G150 [enterobacteria]			
serine protease autotransporter toxin Sat [Escherichia coli G150]	2629	0.0	HAX0282361
Escherichia coli CU758 [enterobacteria]			
serine protease autotransporter toxin Sat [Escherichia coli CU758]	2629	0.0	HAX0287392
Escherichia coli H003 [enterobacteria]			
serine protease autotransporter toxin Sat [Escherichia coli H003]	2629	0.0	HAX0292018
Escherichia coli H006 [enterobacteria]			
serine protease autotransporter toxin Sat [Escherichia coli H006]	2629	0.0	HAX0306524
Escherichia coli CD400 [enterobacteria]			
serine protease autotransporter toxin Sat [Escherichia coli CD400]	2629	0.0	HAX0316414
Escherichia coli C001 [enterobacteria]			
serine protease autotransporter toxin Sat [Escherichia coli C001]	2629	0.0	HAX0325983
Escherichia coli JJ2118 [enterobacteria]			
serine protease autotransporter toxin Sat [Escherichia coli JJ2118]	2629	0.0	HAX0345117
Escherichia coli JJ2134 [enterobacteria]			
serine protease autotransporter toxin Sat [Escherichia coli JJ2134]	2629	0.0	HAX0349609
Escherichia coli JJ2547 [enterobacteria]			
serine protease autotransporter toxin Sat [Escherichia coli JJ2547]	2629	0.0	HAX5573027
Escherichia coli O157:H7 str. EC10 [enterobacteria]			
serine protease autotransporter toxin Sat [Escherichia coli O157:H7 str. EC10]	2628	0.0	HBA3504226
Escherichia coli VR50 [enterobacteria]			
secreted autotranspoter toxin [Escherichia coli VR50]	2628	0.0	AKA92116
Escherichia coli str. 'clone D i2' [enterobacteria]			
Aecreted auto transpoter toxin [Escherichia coli str. 'clone D i2']	2628	0.0	AER85851
Escherichia coli str. 'clone D i14' [enterobacteria]			
Aecreted auto transpoter toxin [Escherichia coli str. 'clone D i14']	2628	0.0	AER90770
Escherichia coli O25b:H4 [enterobacteria]			
eaaA [Escherichia coli O25b:H4]	2628	0.0	ANK03324
Escherichia coli LAU-EC7 [enterobacteria]			
autotransporter [Escherichia coli LAU-EC7]	2628	0.0	ETE30666
Escherichia coli 1-250-04_S4_C2 [enterobacteria]			
serine protease sat autotransporter [Escherichia coli 1-250-04_S4_C2]	2628	0.0	EZJ51354
Escherichia coli 1-250-04_S4_C1 [enterobacteria]			
serine protease sat autotransporter [Escherichia coli 1-250-04_S4_C1]	2628	0.0	EZJ63316
Escherichia coli KTE57 [enterobacteria]			
serine protease sat autotransporter [Escherichia coli KTE57]	2628	0.0	ELE25072
Escherichia coli HVH 77 (4-2605759) [enterobacteria]			
serine protease sat autotransporter [Escherichia coli HVH 77 (4-2605759)]	2628	0.0	EQP64354
Escherichia coli HVH 216 (4-3042952) [enterobacteria]			
serine protease sat autotransporter [Escherichia coli HVH 216 (4-3042952)]	2627	0.0	EQU92710
Escherichia coli 910096-2 [enterobacteria]			
serine protease sat autotransporter [Escherichia coli 910096-2]	2627	0.0	ESE27622

Description	Score	E value	Accession
Escherichia coli 1-110-08_S4_C1 [enterobacteria]			
serine protease sat autotransporter [Escherichia coli 1-110-08_S4_C1]	2627	0.0	EYE09558
Escherichia coli HVH 51 (4-2172526) [enterobacteria]			
serine protease sat autotransporter [Escherichia coli HVH 51 (4-2172526)]	2627	0.0	EQO90481
Escherichia coli O21 [enterobacteria]			
serine protease autotransporter toxin Sat [Escherichia coli O21]	2627	0.0	EEZ6103770
Escherichia coli G199 [enterobacteria]			
serine protease autotransporter toxin Sat [Escherichia coli G199]	2627	0.0	HAX0263157
Escherichia coli O25b:H4-ST131 [enterobacteria]			
serine protease autotransporter toxin Sat [Escherichia coli O25b:H4-ST131]	2627	0.0	MBK3321699
serine protease autotransporter toxin Sat [Escherichia coli O25b:H4-ST131]	2627	0.0	MBK3326635
serine protease autotransporter toxin Sat [Escherichia coli O25b:H4-ST131]	2627	0.0	HAN3152878
serine protease autotransporter toxin Sat [Escherichia coli O25b:H4-ST131]	2627	0.0	HAN2555901
serine protease autotransporter toxin Sat [Escherichia coli O25b:H4-ST131]	2627	0.0	HAN2831583
serine protease autotransporter toxin Sat [Escherichia coli O25b:H4-ST131]	2627	0.0	HAO9828936
Escherichia coli MVAST158 [enterobacteria]			
serine protease autotransporter toxin Sat [Escherichia coli MVAST158]	2627	0.0	HAX0142153

- ### Taxonomy

Taxonomy	Number of hits	Number of Organisms	Description
Enterobacterales	292	82	
. Enterobacteriaceae	2	82	Enterobacteriaceae hits
.. Escherichia	277	76	
... Escherichia coli	197	76	Escherichia coli hits
.... Escherichia coli O1	1	1	Escherichia coli O1 hits
.... Escherichia coli UMEA 3899-1	1	1	Escherichia coli UMEA 3899-1 hits
.... Escherichia coli O39:H4	1	1	Escherichia coli O39:H4 hits
.... Escherichia coli O157:H7	1	2	Escherichia coli O157:H7 hits
..... Escherichia coli O157:H7 str. EC10	1	1	Escherichia coli O157:H7 str. EC10 hits
.... Escherichia coli O25	1	1	Escherichia coli O25 hits
.... Escherichia coli IS35	1	1	Escherichia coli IS35 hits
.... Escherichia coli UMEA 3252-1	1	1	Escherichia coli UMEA 3252-1 hits
.... Escherichia coli HVH 93 (4-5851025)	1	1	Escherichia coli HVH 93 (4-5851025) hits
.... Escherichia coli KN1604	1	1	Escherichia coli KN1604 hits
.... Escherichia coli MH5800	1	1	Escherichia coli MH5800 hits
.... Escherichia coli JJ2657	1	1	Escherichia coli JJ2657 hits
.... Escherichia coli JJ2608	1	1	Escherichia coli JJ2608 hits
.... Escherichia coli JJ2555	1	1	Escherichia coli JJ2555 hits
.... Escherichia coli JJ2550	1	1	Escherichia coli JJ2550 hits
.... Escherichia coli JJ2578	1	1	Escherichia coli JJ2578 hits
.... Escherichia coli JJ2528	1	1	Escherichia coli JJ2528 hits
.... Escherichia coli JJ2508	1	1	Escherichia coli JJ2508 hits
.... Escherichia coli JJ2444	1	1	Escherichia coli JJ2444 hits
.... Escherichia coli ZH063	1	1	Escherichia coli ZH063 hits
.... Escherichia coli ZH071	1	1	Escherichia coli ZH071 hits
.... Escherichia coli CD340	1	1	Escherichia coli CD340 hits
.... Escherichia coli ZH164	1	1	Escherichia coli ZH164 hits
.... Escherichia coli U024	1	1	Escherichia coli U024 hits
.... Escherichia coli U004	1	1	Escherichia coli U004 hits
.... Escherichia coli SaT158	1	1	Escherichia coli SaT158 hits
.... Escherichia coli SaT040	1	1	Escherichia coli SaT040 hits
.... Escherichia coli SaT049	1	1	Escherichia coli SaT049 hits
.... Escherichia coli MVAST179	1	1	Escherichia coli MVAST179 hits
.... Escherichia coli MVAST131	1	1	Escherichia coli MVAST131 hits
.... Escherichia coli JJ2038	1	1	Escherichia coli JJ2038 hits
.... Escherichia coli JJ2183	1	1	Escherichia coli JJ2183 hits
.... Escherichia coli MVAST084	1	1	Escherichia coli MVAST084 hits
.... Escherichia coli MVAST077	1	1	Escherichia coli MVAST077 hits
.... Escherichia coli JJ1908	1	1	Escherichia coli JJ1908 hits
.... Escherichia coli JJ2050	1	1	Escherichia coli JJ2050 hits
.... Escherichia coli JJ2193	1	1	Escherichia coli JJ2193 hits
.... Escherichia coli JJ2643	1	1	Escherichia coli JJ2643 hits

.... Escherichia coli JJ2016	1	1	Escherichia coli JJ2016 hits
.... Escherichia coli MVAST038	1	1	Escherichia coli MVAST038 hits
.... Escherichia coli MVAST046	1	1	Escherichia coli MVAST046 hits
.... Escherichia coli H061	1	1	Escherichia coli H061 hits
.... Escherichia coli MVAST014	1	1	Escherichia coli MVAST014 hits
.... Escherichia coli MVAST0036	1	1	Escherichia coli MVAST0036 hits
.... Escherichia coli JJ1897	1	1	Escherichia coli JJ1897 hits
.... Escherichia coli G213	1	1	Escherichia coli G213 hits
.... Escherichia coli G132	1	1	Escherichia coli G132 hits
.... Escherichia coli JJ1901	1	1	Escherichia coli JJ1901 hits
.... Escherichia coli H016	1	1	Escherichia coli H016 hits
.... Escherichia coli G150	1	1	Escherichia coli G150 hits
.... Escherichia coli CU758	1	1	Escherichia coli CU758 hits
.... Escherichia coli H003	1	1	Escherichia coli H003 hits
.... Escherichia coli H006	1	1	Escherichia coli H006 hits
.... Escherichia coli CD400	1	1	Escherichia coli CD400 hits
.... Escherichia coli C001	1	1	Escherichia coli C001 hits
.... Escherichia coli JJ2118	1	1	Escherichia coli JJ2118 hits
.... Escherichia coli JJ2134	1	1	Escherichia coli JJ2134 hits
.... Escherichia coli JJ2547	1	1	Escherichia coli JJ2547 hits
.... Escherichia coli VR50	1	1	Escherichia coli VR50 hits
.... Escherichia coli str. 'clone D i2'	1	1	Escherichia coli str. 'clone D i2' hits
.... Escherichia coli str. 'clone D i14'	1	1	Escherichia coli str. 'clone D i14' hits
.... Escherichia coli O25b:H4	1	1	Escherichia coli O25b:H4 hits
.... Escherichia coli LAU-EC7	1	1	Escherichia coli LAU-EC7 hits
.... Escherichia coli 1-250-04_S4_C2	1	1	Escherichia coli 1-250-04_S4_C2 hits
.... Escherichia coli 1-250-04_S4_C1	1	1	Escherichia coli 1-250-04_S4_C1 hits
.... Escherichia coli KTE57	1	1	Escherichia coli KTE57 hits
.... Escherichia coli HVH 77 (4-2605759)	1	1	Escherichia coli HVH 77 (4-2605759) hits
.... Escherichia coli HVH 216 (4-3042952)	1	1	Escherichia coli HVH 216 (4-3042952) hits
.... Escherichia coli 910096-2	1	1	Escherichia coli 910096-2 hits
.... Escherichia coli 1-110-08_S4_C1	1	1	Escherichia coli 1-110-08_S4_C1 hits
.... Escherichia coli HVH 51 (4-2172526)	1	1	Escherichia coli HVH 51 (4-2172526) hits
.... Escherichia coli O21	1	1	Escherichia coli O21 hits
.... Escherichia coli G199	1	1	Escherichia coli G199 hits
.... Escherichia coli O25b:H4-ST131	6	1	Escherichia coli O25b:H4-ST131 hits
.... Escherichia coli MVAST158	1	1	Escherichia coli MVAST158 hits
.. Shigella	13	5	
... Shigella flexneri	7	1	Shigella flexneri hits
... Shigella boydii	2	1	Shigella boydii hits
... unclassified Shigella	2	2	
.... Shigella sp. FC2045	1	1	Shigella sp. FC2045 hits
.... Shigella sp. FC2928	1	1	Shigella sp. FC2928 hits
... Shigella sonnei	2	1	Shigella sonnei hits
