# Supplementary material for: Secreted Autotransporter Toxin (Sat) Mediates Innate Immune System Evasion
Source: Front Immunol. 2022 Feb 17;13:844878. doi: 10.3389/fimmu.2022.844878 (PMC8891578; doi:10.3389/fimmu.2022.844878)
Supplement: Supplementary file 2 [file DataSheet_2.pdf]

Summary

1. Notes

2. Result Statistics

Figure 1. False discovery rate (FDR) curve. X axis is the number of peptide-spectrum matches (PSM) being kept. Y axis is the corresponding FDR.

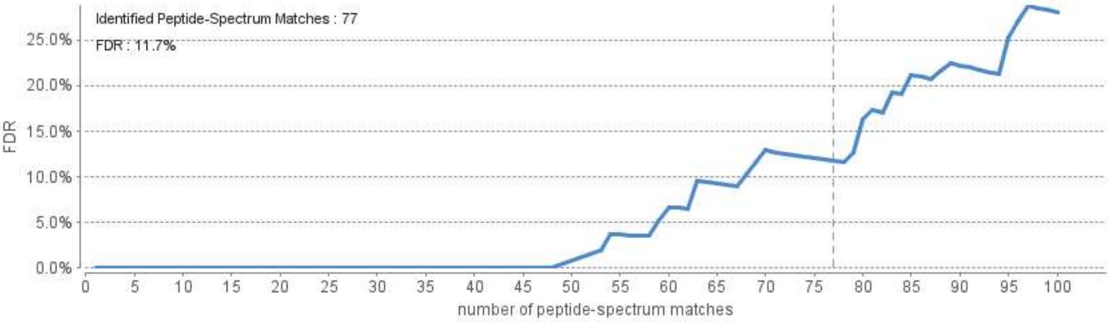

Figure 2. PSM score distribution. **(a)** Distribution of PEAKS peptide score; **(b)** Scatterplot of PEAKS peptide score versus precursor mass error.

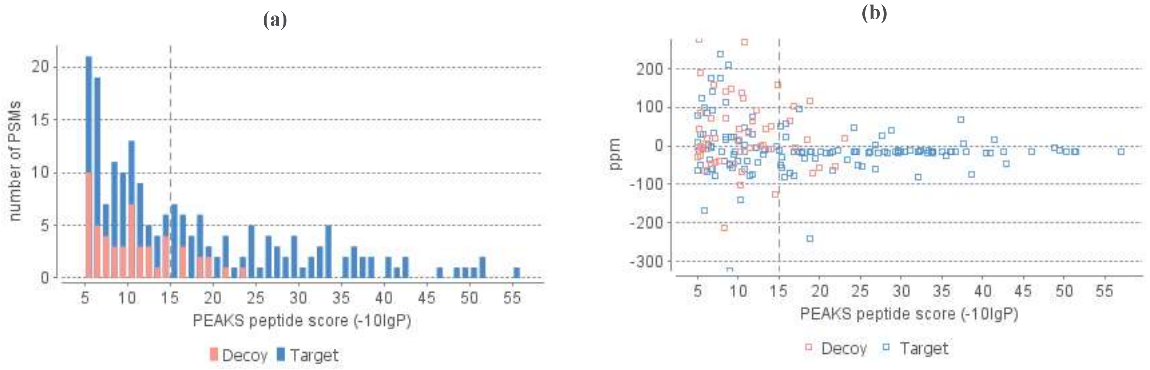

Figure 3. De novo result validation. Distribution of residue local confidence: **(a)** Residues in de novo sequences validated by confident database peptide assignment; **(b)** Residues in "de novo only" sequences.

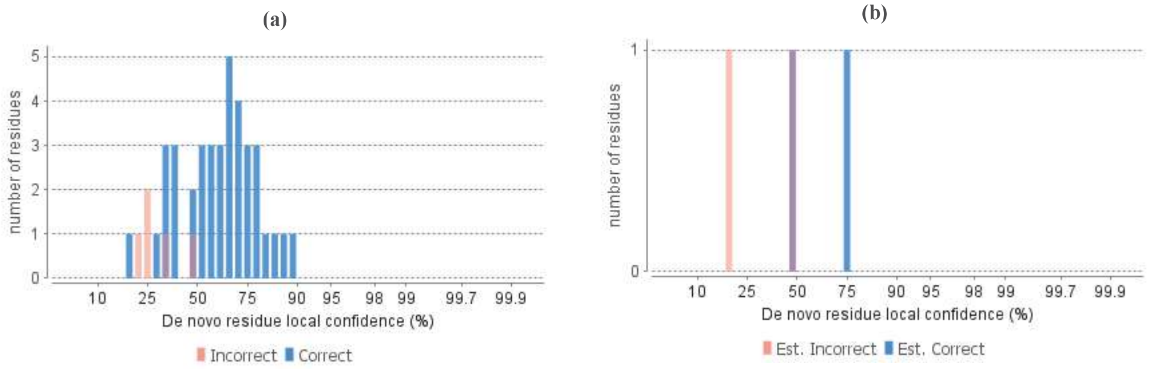

Table 1. Statistics of data.

# of MS Scans	0
# of MS/MS Scans	344

Table 2. Result filtration parameters.

Peptide -10lgP	≥15
Protein -10lgP	≥20
Proteins unique peptides	≥0
De novo ALC Score	≥50%

Table 3. Statistics of filtered result.

Peptide-Spectrum Matches	77
Peptide Sequences	74

Table 4. PTM profile.

Name	ΔMass	#PSM	Position
Carbamidomethyl	57.02	6	N-term
Deamidation	.98	2	NQ
Acetylation	42.01	1	N-term
Methyl ester	14.02	1	E
Carbamidomethyl	57.02	1	C
Pyro-glu from Q	-17.03	1	N-term

Protein Groups	5
Proteins	7
Proteins (#Unique Peptides)	1 (>2); 0 (=2); 6 (=1);
FDR (Peptide-Spectrum Matches)	11.7%
FDR (Peptide Sequences)	12.2%
FDR (Protein)	42.9%
De Novo Only Spectra	1

3. Experiment Control

Figure 4. Precursor mass error of peptide-spectrum matches (PSM) in filtered result. **(a)** Distribution of precursor mass error in ppm; **(b)** Scatterplot of precursor m/z versus precursor mass error in ppm.

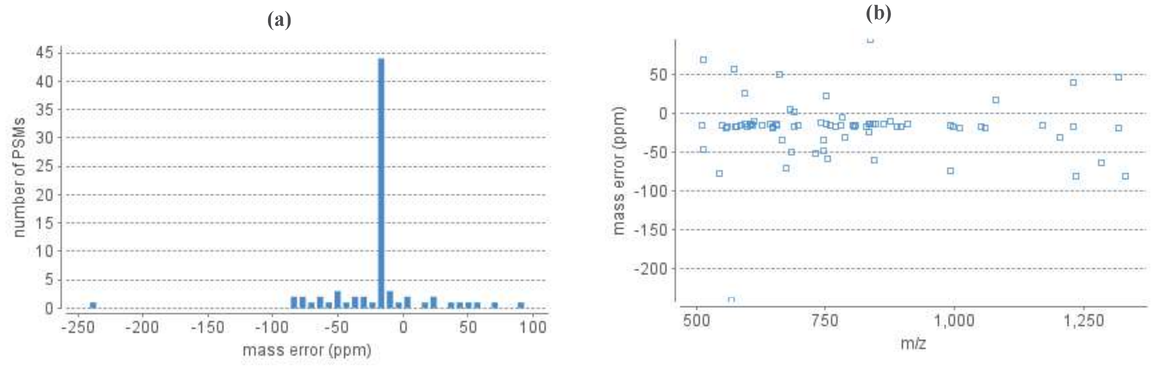

Table 5. Number of identified peptides in each sample by the number of missed cleavages

Missed Cleavages	0	1	2	3	4+
Sample 1	67	7	0	0	0

4. Other Information

Table 6. Search parameters.

Query Type: Homology Match
Fixed Modifications:
Carbamidomethylation: 57.02
Variable Modifications:
Oxidation (M): 15.99
Fragment ion tolerance: 0.1
L equals I: true
Q equals K: true
Report number: 1
Maximum # of PTMs: 3
De novo score (ALC%) threshold: 15
Peptide hit threshold (-10logP): 30.0
Peaks run ID: 12
Merge Options: 0.1 min. 0.1 Da
Precursor Options: corrected
Charge Options: no correction
Filter Options: no filter
Process: true

Table 7. Instrument parameters.

Fractions: CLAUDIA_33.mgf
Ion Source: ESI(nano-spray)
Fragmentation Mode: CID, CAD(y and b ions)
MS Scan Mode: 3D Ion Trap
MS/MS Scan Mode: Time of Flight (TOF)

Protein List

Protein Group	Protein ID	Accession	-10lgP	Coverage (%)	#Peptides	#Unique	PTM	Avg. Mass	Description
**1**	195	Q8FDW4|SAT_ECOL6	202.60	42	52	51	Y	140042	Serine protease sat autotransporter OS=Escherichia coli O6:H1 (strain CFT073 / ATCC 700928 / UPEC) OX=199310 GN=sat PE=1 SV=2
**3**	378	A7FCR5|LIGB_YERP3	27.63	1	1	1	N	63624	DNA ligase B OS=Yersinia pseudotuberculosis serotype O:1b (strain IP 31758) OX=349747 GN=ligB PE=3 SV=1
**4**	463	Q76N89|HECW1_HUMAN	26.79	0	1	1	Y	179553	E3 ubiquitin-protein ligase HECW1 OS=Homo sapiens OX=9606 GN=HECW1 PE=1 SV=3
**2**	373	B5Z6D1|CH60_HELPG	23.45	1	1	1	Y	58228	60 kDa chaperonin OS=Helicobacter pylori (strain G27) OX=563041 GN=groL PE=3 SV=1
**2**	374	B6JPA7|CH60_HELP2	23.45	1	1	1	Y	58300	60 kDa chaperonin OS=Helicobacter pylori (strain P12) OX=570508 GN=groL PE=3 SV=1
**2**	372	Q7U317|CH60_HELHP	23.45	1	1	1	Y	57651	60 kDa chaperonin OS=Helicobacter hepaticus (strain ATCC 51449 / 3B1) OX=235279 GN=groL PE=3 SV=1
**6**	207	Q82WT5|CYSA_NITEU	21.49	2	1	1	N	40893	Sulfate/thiosulfate import ATP-binding protein CysA OS=Nitrosomonas europaea (strain ATCC 19718 / CIP 103999 / KCTC 2705 / NBRC 14298) OX=228410 GN=cysA PE=3 SV=1
**total 7 proteins**									

Q8FDW4|SAT_ECOL6

back to list

| Protein Coverage | Supporting Peptides |

Protein Coverage:

```
   1 MNKIYSLKYS AATGGLIAVS ELAKRVSGKT NRKLVATMLS LAVAGTVNAA NIDISNVWAR DYLDLAQNKG IFQPGATDVT
  81 ITLKNGDKFS FHNLSIPDFS GAAASGAATA IGGSYSVTVA HNKKNPQAAE TQVYAQSSYR VVDRRNSNDF EIQRLNKFVV
 161 ETVGATPAET NPTTYSDALE RYGIVTSDGS KKIIGFRAGS GGTSFINGES KISTNSAYSH DLLSASLFEV TQWDSYGMMI
 241 YKNDKTFRNL EIFGDSGSGA YLYDNKLEKW VLVGTTHGIA SVNGDQLTWI TKYNDKLVSE LKDTYSHKIN LNGNNVTIKN
 321 TDITLHQNNA DTTGTQEKIT KDKDIVFTNG GDVLFKDNLD FGSGGIIFDE GHEYNINGQG FTFKGAGIDI GKESIVNWNA
 401 LYSSDDVLHK IGPGTLNVQK KQGANIKIGE GNVILNEEGT FNNIYLASGN GKVILNKDNS LGNDQYAGIF FTKRGGTLDL
 481 NGHNQTFTRI AATDDGTTIT NSDTTKEAVL AINNEDSYIY HGNINGNIKL THNINSQDKK TNAKLILDGS VNTKNDVEVS
 561 NASLTMQGHA TEHAIFRSSA NHCSLVFLCG TDWVTVLKET ESSYNKKFNS DYKSNNQQTS FDQPDWKTGV FKFDTLHLNN
 641 ADFSISRNAN VEGNISANKS AITIGDKNVY IDNLAGKNIT NNGFDFKQTI STNLSIGETK FTGGITAHNS QIAIGDQAVV
 721 TLNGATFLDN TPISIDKGAK VIAQNSMFTT KGIDISGELT MMGIPEQNSK TVTPGLHYAA DGFRLSGGNA NFIARNMASV
 801 TGNIYADDAA TITLGQPETE TPTISSAYQA WAETLLYGFD TAYRGAITAP KATVSMNNAI WHLNSQSSIN RLETKDSMVR
 881 FTGDNGKFTT LTVNNLTIDD SAFVLRANLA QADQLVVNKS LSGKNNLLLV DFIEKNGNSN GLNIDLVSAP KGTAVDVFKA
 961 TTRSIGFSDV TPVIEQKNDT DKATWTLIGY KSVANADAAK KATLLMSGGY KAFLAEVNNL NKRMGDLRDI NGESGAWARI
1041 ISGTGSAGGG FSDNYTHVQV GADNKHELDG LDLFTGVTMT YTDSHAGSDA FSGETKSVGA GLYASAMFES GAYIDLIGKY
1121 VHHDNEYTAT FAGLGTRDYS SHSWYAGAEV GYRYHVTDSA WIEPQAELVY GAVSGKQFSW KDQGMNLTMK DKDFNPLIGR
1201 TGVDVGKSFS GKDWKVTARA GLGYQFDLFA NGETVLRDAS GEKRIKGEKD GRMLMNVGLN AEIRDNLRFG LEFEKSAFGK
1281 YNVDNAINAN FRYSF
```

c Carba
c Carba
d Deam
P Pyro-
* Carba
X Mutat

Supporting Peptides:

Peptide	Uniq	-10lgP	Mass	ppm	m/z	z	RT	Scan	#Spec	Start	End	PTM
K.NGNSN(+.98)GLNIDLVSAPK.G	Y	56.93	1612.8057	-15.5	807.3976	2	0.41	850	1	936	951	Deamidation (NQ)
K.QTISTNLSIGETK.F	Y	51.40	1390.7303	-15.6	696.3616	2	0.34	802	1	688	700	
R.SIGFSDVTPVIEQK.N	Y	51.27	1518.7930	-15.8	760.3918	2	0.42	828	1	964	977	
K.TVTPGLHYAADGFR.L	Y	50.18	1503.7469	-14.7	752.8697	2	0.35	824	1	771	784	
K.FDTLHLNNADFSISR.N	Y	49.46	1748.8481	-11.3	875.4215	2	0.43	887	1	633	647	
K.GIFQ(+.98)PGATDVTITLK.N	Y	48.73	1560.8398	-5.7	781.4227	2	0.45	840	1	70	84	Deamidation (NQ)
K.DNSLGNDQYAGIFFTK.R	Y	46.00	1788.8318	-16.9	895.4080	2	0.46	896	1	458	473	
K.IGPGTLNVQK.K	Y	42.90	1025.5869	-46.1	513.7771	2	0.27	698	1	411	420	
K.NDVEVSNASLTMQGHATEHAIFR.S	Y	42.55	2526.1921	-14.8	843.0589	3	0.50	865	1	555	577	
R.DYLDLAQNK.G	Y	41.37	1078.5294	16.3	1079.5543	1	0.35	962	1	61	69	
L.VFLC(+57.02)GTDWVTVLK.E	Y	40.92	1536.8010	-17.6	769.3943	2	0.51	835	1	586	598	Carbamidomethylation
R.LSGGNANFIAR.N	Y	40.35	1118.5833	-17.4	560.2892	2	0.30	718	1	785	795	
K.DNLDFGSGGIIFDEGHEY.N	Y	38.69	1983.8486	-75.1	992.8571	2	0.50	923	2	357	374	
R.IAATDDGTTITNSDTTK.E	Y	38.02	1723.8112	-14.2	862.9006	2	0.27	883	1	490	506	
W.NALYSSDDVLHK.I	Y	37.66	1360.6622	5.4	681.3420	2	0.43	787	1	399	410	
R.YGN(sub I)VTSDGSK.K	Y	37.36	1026.4618	69.4	514.2738	2	0.27	700	1	182	191	Mutation
G.F(+57.02)DTAYR.G	Y	36.70	828.3766	-17.0	829.3698	1	0.26	856	1	839	844	Carbamidomethylation (DHKE, X@N-term)
R.AGSGGTSFINGESK.I	Y	36.03	1310.6102	-13.9	656.3033	2	0.26	768	1	198	211	
K.DKDIVFTL(sub N)GGDVLFK.D	Y	35.54	1665.8977	-25.0	833.9353	2	0.43	858	1	342	356	Mutation
K.INLNGNNVTIK.N	Y	35.08	1198.6670	-16.8	600.3307	2	0.33	736	1	309	319	
K.GIDISGELTMMGIPEQNSK.T	Y	33.82	2018.9652	-18.3	1010.4714	2	0.51	926	1	752	770	
K.DKDIVFTNGGDVLFK.D	Y	33.78	1666.8566	-14.8	834.4232	2	0.44	859	1	342	356	
K.NGNSNGLNIDLVSAPK.G	Y	33.69	1611.8217	-17.1	806.9043	2	0.39	849	1	936	951	
K.V(+57.02)ILNK.D	Y	33.35	642.4064	-13.7	643.4049	1	0.23	758	1	453	457	Carbamidomethylation (DHKE, X@N-term)
K.LILDGSVNTK.N	Y	33.18	1058.5972	-18.8	1059.5846	1	0.33	948	1	545	554	
G.M(+57.02)MIYK.N	Y	32.32	741.3553	-11.6	742.3540	1	0.29	812	1	238	242	Carbamidomethylation (DHKE, X@N-term)
K.T(+57.02)GVFK.F	Y	32.20	607.3329	-16.5	608.3302	1	0.22	744	1	628	632	Carbamidomethylation (DHKE, X@N-term)
R.NLEIFGDSGSGAY.L	Y	32.05	1328.5884	-81.6	1329.4873	1	0.45	1016	1	249	261	
K.SAITIGDK.N	Y	31.99	803.4388	-15.2	804.4339	1	0.25	847	1	660	667	
K.Q(-17.03)GANIK.I	Y	31.08	612.3231	-11.4	613.3234	1	0.15	747	1	422	427	Pyro-glu from Q
R.GAITAPK.A	Y	30.38	656.3857	-15.6	657.3827	1	0.20	772	1	845	851	

total 57 peptides

Peptide	Uniq	-10lgP	Mass	ppm	m/z	z	RT	Scan	#Spec	Start	End	PTM
K.GIFQPGATDVTITLK.N	Y	29.93	1559.8558	-16.0	780.9227	2	0.46	838	1	70	84	
K.GTAVDVFK.A	Y	29.67	835.4440	-13.7	836.4398	1	0.32	861	1	952	959	
T.VGATPAETNPTTYSDALER.Y	Y	29.62	1991.9436	-17.7	996.9614	2	0.35	925	1	163	181	
K.TGVFK.F	Y	29.25	550.3115	-15.6	551.3102	1	0.20	714	1	628	632	
R.NANVEGNISANK.S	Y	28.73	1229.6000	40.1	1230.6566	1	0.22	989	2	648	659	
K.NTDITLHQNNADTTGTQEK.I	Y	28.22	2099.9719	-17.5	1050.9749	2	0.26	946	1	320	338	
K.LVSELK.D	Y	27.89	687.4167	-17.0	688.4123	1	0.24	794	1	297	302	
K.TVTPGLHY.A	Y	27.08	886.4548	-17.4	887.4467	1	0.30	890	1	771	778	
T.E(+57.02)(+14.02)HAIFR.S	Y	26.77	842.4399	-61.3	843.3955	1	0.46	869	1	572	577	Carbamidomethylation (DHKE, X@N-term); Methyl ester
K.SNNQQTSFDQPDWK.T	Y	26.14	1693.7332	-14.0	847.8620	2	0.32	872	1	614	627	
F.LN(sub D)NTPISIDK.G	Y	26.07	1113.6030	-18.4	557.7985	2	0.30	716	1	728	737	Mutation
K.GAGIDIGK.E	Y	25.11	729.4021	-52.0	730.3715	1	0.31	811	1	385	392	
K.L(+57.02)VSELK.D	Y	24.72	744.4381	-49.0	745.4089	1	0.33	815	1	297	302	Carbamidomethylation (DHKE, X@N-term)
Y.LYDNKLEK.W	Y	24.51	1021.5444	-16.0	511.7713	2	0.23	695	1	262	269	
W.HLNSQSSINR.L	Y	24.37	1154.5792	-16.8	578.2872	2	0.19	728	1	862	871	
K.NNLLLVDFIEK.N	Y	24.19	1316.7340	45.6	1317.8013	1	0.52	1012	2	925	935	
K.VILNK.D	Y	24.11	585.3850	-15.7	586.3831	1	0.20	731	1	453	457	
K.VIAQNSMF.T	Y	22.16	908.4426	-13.4	909.4377	1	0.36	899	1	741	748	
K.FVVETVGATPAETNPTTYSDALER.Y	Y	21.62	2567.2390	-63.7	1284.5450	2	0.43	999	1	158	181	
K.LTHNINSQDKK.T	Y	20.69	1296.6786	-18.9	649.3343	2	0.20	761	1	530	540	
K.NITNNGFDFK.Q	Y	20.12	1168.5513	-16.3	1169.5396	1	0.35	982	1	678	687	
K.DSMVR.F	Y	18.97	606.2795	-14.4	607.2781	1	0.08	743	1	876	880	
K.FVVET.V	Y	17.84	593.3060	-14.5	594.3047	1	0.31	734	1	158	162	
K.AFLAEV.N	Y	17.32	648.3483	-16.7	649.3447	1	0.39	762	1	1012	1017	
K.IIGFR.A	N	16.73	604.3696	-15.8	605.3674	1	0.31	741	1	193	197	
K.ISTNSAYSHDLLSASLFEVTQW.D	Y	15.60	2468.1860	-81.3	1235.0000	2	0.59	991	1	212	233	
**total 57 peptides**												

A7FCR5|LIGB_YERP3

back to list

| Protein Coverage | Supporting Peptides |

Protein Coverage:

```
  1 MNILNLKIIM FLLISNIIVV GGAWATSTCP DWPATRIAVE INALEQQLNK WSAAYHQQGH SPVTDDIYDQ LQDKLRVWQS
 81 CRGLPDKTES QPIPGKGQFL HPVAHTGLKK LKDETALTRW MAGRKNLWVQ PKVDGVAVTL VYHGGKLVQL LSRGNGVKGQ
161 NWTEKAPFIS AIPQYIANAP ALLTLQGELF LLMDGHQQAK SGGVNARSTV AGALMRKSPS PLLAQVGVFI WAWPDGPTTM
241 KEKVALLQVM GFPFTAKYSE PVMSHLDVVQ WRQFWFQAPL PFVTDGVVVR QEEEPAGRYW QATPGQWSMA WKYPPLQHIA
321 EVKDIHFTLG RTGKGTVVLE VLPIKIDDKW IRRVNIGSVT RWKQWDIAPG DHITLALAGH GIPRLDNVVW RVHQRNTITA
401 PNWDKFHQLS CFQRLPHGCE PQFLSRLIWL SGPGGLDIGG IGGGFWQELI HHELINDLVG WLLLTPEQIA SIPGIGNARA
481 EKIYQQFQRA KQQPFSRWLL ALGFPQVVSV DAQWQVVLRR SLSEWATMAG IGQMRAKQIK HFLDHPDVQA LADFLSTQKV
561 VGFELTE
```

Supporting Peptides:

Peptide	Uniq	-10lgP	Mass	ppm	m/z	z	RT	Scan	#Spec	Start	End	PTM
H.FTLGR.T	Y	27.63	592.3333	25.7	593.3558	1	0.25	733	1	327	331	
**total 1 peptides**												

Q76N89|HECW1_HUMAN

back to list

| Protein Coverage | Supporting Peptides |

Protein Coverage:

```
   1 MLLHLCSVKN LYQNRFLGLA AMASPSRNSQ SRRRCKEPLR YSYNPDQFHN MDLRGGPHDG VTIPRSTSDT DLVTSDSRST
  81 LMVSSSYYSI GHSQDLVIHW DIKEEVDAGD WIGMYLIDEV LSENFLDYKN RGVNGSHRGQ IIWKIDASSY FVEPETKICF
 161 KYYHGVSGAL RATTPSVTVK NSAAPIFKSI GADETVQGQG SRRLISFSLS DFQAMGLKKG MFFNPDPYLK ISIQPGKHSI
 241 FPALPHHGQE RRSKIIGNTV NPIWQAEQFS FVSLPTDVLE IEVKDKFAKS RPIIKRFLGK LSMPVQRLLE RHAIGDRVVS
 321 YTLGRRLPTD HVSGQLQFRF EITSSIHPDD EEISLSTEPE SAQIQDSPMN NLMESGSGEP RSEAPESSES WKPEQLGEGS
 401 VPDGPGNQSI ELSRPAEEAA VITEAGDQGM VSVGPEGAGE LLAQVQKDIQ PAPSAEELAE QLDLGEEASA LLLEDGEAPA
 481 STKEEPLEEE ATTQSRAGRE EEEKEQEEEG DVSTLEQGEG RLQLRASVKR KSRPCSLPVS ELETVIASAC GDPETPRTHY
 561 IRIHTLLHSM PSAQGGSAAE EEDGAEEEST LKDSSEKDGL SEVDTVAADP SALEEDREEP EGATPGTAHP GHSGGHFPSL
 641 ANGAAQDGDT HPSTGSESDS SPRQGGDHSC EGCDASCCSP SCYSSSCYST SCYSSSCYSA SCYSPSCYNG NRFASHTRFS
 721 SVDSAKISES TVFSSQDDEE EENSAFESVP DSMQSPELDP ESTNGAGPWQ DELAAPSGHV ERSPEGLESP VAGPSNRREG
 801 ECPILHNSQP VSQLPSLRPE HHHYPTIDEP LPPNWEARID SHGRVFYVDH VNRTTTWQRP TAAATPDGMR RSGSIQQMEQ
 881 LNRRYQNIQR TIATERSEED SGSQSCEQAP AGGGGGGGSD SEAESSQSSL DLRREGSLSP VNSQKITLLL QSPAVKFITN
 961 PEFFTVLHAN YSAYRVFTSS TCLKHMILKV RRDARNFERY QHNRDLVNFI NMFADTRLEL PRGWEIKTDQ QGKSFFVDHN
1041 SRATTFIDPR IPLQNGRLPN HLTHRQHLQR LRSYSAGEAS EVSRNRGASL LARPGHSLVA AIRSQHQHES LPLAYNDKIV
1121 AFLRQPNIFE MLQERQPSLA RNHTLREKIH YIRTEGNHGL EKLSCDADLV ILLSLFEEEI MSYVPLQAAF HPGYSFSPRC
1201 SPCSSPQNSP GLQRASARAP SPYRRDFEAK LRNFYRKLEA KGFGQGPGKI KLIIRRDHLL EGTFNQVMAY SRKELQRNKL
1281 YVTFVGEEGL DYSGPSREFF FLLSQELFNP YYGLFEYSAN DTYTVQISPM SAFVENHLEW FRFSGRILGL ALIHQYLLDA
1361 FFTRPFYKAL LRLPCDLSDL EYLDEEFHQS LQWMKDNNIT DILDLTFTVN EEVFGQVTER ELKSGGANTQ VTEKNKKEYI
1441 ERMVKWRVER GVVQQTEALV RGFYEVVDSR LVSVFDAREL ELVIAGTAEI DLNDWRNNTE YRGGYHDGHL VIRWFWAAVE
1521 RFNNEQRLRL LQFVTGTSSV PYEGFAALRG SNGLRRFCIE KWGKITSLPR AHTCFNRLDL PPYPSYSMLY EKLLTAVEET
1601 STFGLE
```

Supporting Peptides:

Peptide	Uniq	-10lgP	Mass	ppm	m/z	z	RT	Scan	#Spec	Start	End	PTM
A.MASPE(sub S)R.N	Y	26.79	689.3167	2.0	690.3253	1	0.14	799	1	22	27	Mutation
**total 1 peptides**												

B5Z6D1|CH60_HELPG

back to list

| Protein Coverage | Supporting Peptides |

Protein Coverage:

```
   1 MAKEIKFSDS ARNLLFEGVR QLHDAVKVTM GPRGRNVLIQ KSYGAPSITK DGVSVAKEIE LSCPVANMGA QLVKEVASKT
  81 ADAAGDGTTT ATVLAYSIFK EGLRNITAGA NPIEVKRGMD KAAEAIINEL KKASKKVGGK EEITQVATIS ANSDHNIGKL
 161 IADAMEKVGK DGVITVEEAK GIEDELDVVE GMQFDRGYLS PYFVTNAEKM TAQLDNAYIL LTDKKISSMK DILPLLEKTM
 241 KEGKPLLIIA EDIEGEALTT LVVNKLRGVL NIAAVKAPGF GDRRKEMLKD IAVLTGGQVI SEELGLSLEN AEVEFLGKAG
 321 RIVIDKDNTT IVDGKGHSDD VKDRVAQIKT QIASTTSDYD KEKLQERLAK LSGGVAVIKV GAASEVEMKE KKDRVDDALS
 401 ATKAAVEEGI VIGGGAALIR AAQKVHLNLH DDEKVGYEII MRAIKAPLAQ IAINAGYDGG VVVNEVEKHE GHFGFNASNG
 481 KYVDMFKEGI IDPLKVERIA LQNAVSVSSL LLTTEATVHE IKEEKAAPAM PDMGGMGGMG GMGGMM
```

Supporting Peptides:

Peptide	Uniq	-10lgP	Mass	ppm	m/z	z	RT	Scan	#Spec	Start	End	PTM
N.D(sub T)TIVDGK.G	Y	23.45	746.3810	-35.4	747.3618	1	0.40	817	1	329	335	Mutation
**total 1 peptides**												

B6JPA7|CH60_HELP2

back to list

| Protein Coverage | Supporting Peptides |

Protein Coverage:

```
   1 MAKEIKFSDS ARNLLFEGVR QLHDAVKVTM GPRGRNVLIQ KSYGAPSITK DGVSVAKEIE LSCPVANMGA QLVKEVASKT
  81 ADAAGDGTTT ATVLAYSIFK EGLRNITAGA NPIEVKRGMD KAAEAIINEL KKASKKVGGK EEITQVATIS ANSDHNIGKL
 161 IADAMEKVGK DGVITVEEAK GIEDELDVVE GMQFDRGYLS PYFVTNAEKM TAQLDNAYIL LTDKKISSMK DILPLLEKTM
 241 KEGKPLLIIA EDIEGEALTT LVVNKLRGVL NIAAVKAPGF GDRRKEMLKD IAILTGGQVI SEELGLSLEN AEVEFLGKAG
 321 RIVIDKDNTT IVDGKGHSDD VKDRVVQIKT QIASTTSDYD KEKLQERLAK LSGGVAVIKV GAASEVEMKE KKDRVDDALS
 401 ATKAAVEEGI VIGGGAALIR AAQKVHLNLH DDEKVGYEII MRAIKAPLAQ IAINAGYDGG VVVNEVEKHE GHFGFNASNG
 481 KYVDMFKEGI IDPLKVERIA LQNAVSVSSL LLTTEATVHE IKEEKATPAM PDMGGMGGMG GMGGMM
```

Supporting Peptides:

Peptide	Uniq	-10lgP	Mass	ppm	m/z	z	RT	Scan	#Spec	Start	End	PTM
N.D(sub T)TIVDGK.G	Y	23.45	746.3810	-35.4	747.3618	1	0.40	817	1	329	335	Mutation
**total 1 peptides**												

Q7U317|CH60_HELHP

back to list

| Protein Coverage | Supporting Peptides |

Protein Coverage:

1 MASKEINFSD SARNKLYEGI KQLSDAVKVT MGPKGRNVLI QKSYGAPTIT KDGVSVAKEI ELADPIANMG AQLVKEVASK
81 TADAAGDGTT TATVLAYSIY KEGLRNITAG ANPIEVKRGM DKASAAIIEE LKKSSKKIGG KSDIAQVATI SANSDENIGA
161 LIAEAMEKVG KDGVITVEEA KGINDELSVV EGMQFDRGYL SAYFVTNTDK MTAQLENAYV LLTDKKISNM KEILPLLEAT
241 MQSGKPLLII AEDIEGEALT TLVVNKLRGV LNVSAVKAPG FGDRRKAMLQ DIAILTGGQV ISEELGKTLE AATLADLGSA
321 ARIVIDKDN**T TIVDGK**GKTK DVKDRIAQIK TEIENTTSDY DREKLQERLA KLSGGVAVIK VGAASEVEMK EKKDRVDDAL
D
401 SATKAAVDEG IVIGGGSALI RASQKVKLKL EGDEAIGYDI IKRAIKAPLA QIATNAGYDA GVVVNEVEKN SKDGFGFNAT
481 TGEYVDMFKE GIIDPLKVTR VALQNAVSVS SLLLTTEATI NEIKEDKPAP AMPDMGGMGG MGGMM

Supporting Peptides:

Peptide	Uniq	-10lgP	Mass	ppm	m/z	z	RT	Scan	#Spec	Start	End	PTM
N.D(sub T)TIVDGK.G	Y	23.45	746.3810	-35.4	747.3618	1	0.40	817	1	330	336	Mutation
**total 1 peptides**												

Q82WT5|CYSA_NITEU

back to list

| Protein Coverage | Supporting Peptides |

Protein Coverage:

1 MTIEIHDLSK QFGSFTALND INLK**VNPGEL** LALLGPSGSG KTTLLRVIAG LETADSGQVL FNEEDSTDKH IRDRHVGFVF
81 QHYALFRNMT IFENVAFGLR VRPRKQRPNA PEINHRVTEL LQLVQLDWLA DRYPHQLSGG QRQRIALARA LAVEPSVLLL
161 DEPFGALDAK VRKELRAWLR KLHDDMHITS VFVTHDQEEA LEVADRIVVM NRGRIEQIGT PDEVYEKPAN PFVYEFLGHV
241 NLFHGRVHQG HAWIGDLEVD APEYSEAEDL SAIAYVRPHD IEVDRTLNGE PALAAHIVHI LAIGPVVRLE LAGKDNQSTN
321 SIYAEISKER FRELQLARGD QVFIKPRKLD LFPNHAQNGS IH

Supporting Peptides:

Peptide	Uniq	-10lgP	Mass	ppm	m/z	z	RT	Scan	#Spec	Start	End	PTM
K.VNPGEL.L	Y	21.49	627.3228	-16.5	628.3197	1	0.28	752	1	25	30	
**total 1 peptides**												

Prepared with PEAKS ™ (bioinfor.com)
