# Supplementary figures and images for: Secreted Autotransporter Toxin (Sat) Mediates Innate Immune System Evasion
Source: Front Immunol. 2022 Feb 17;13:844878. doi: 10.3389/fimmu.2022.844878 (PMC8891578; doi:10.3389/fimmu.2022.844878)

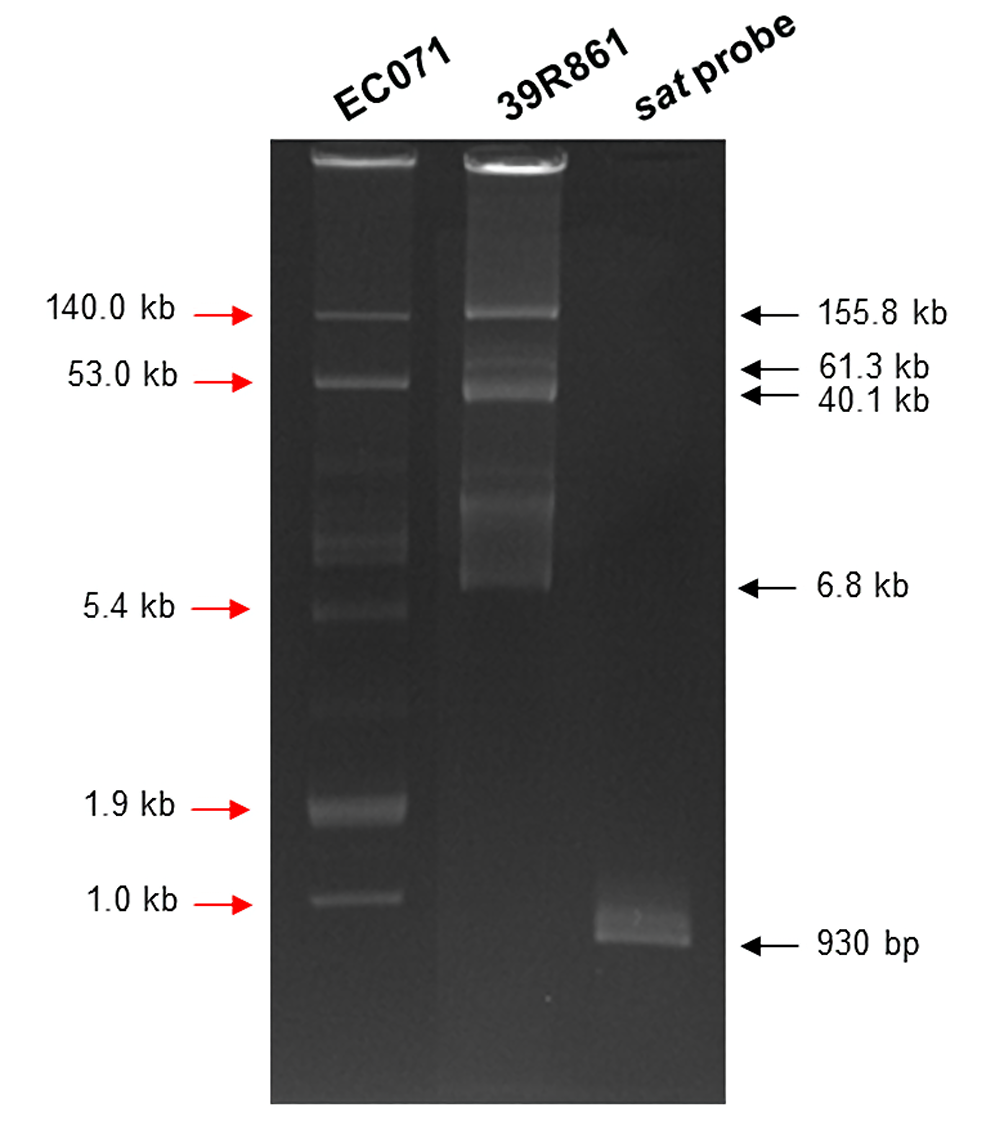

Supplement: Supplementary file 3 [file Image_1.tif]

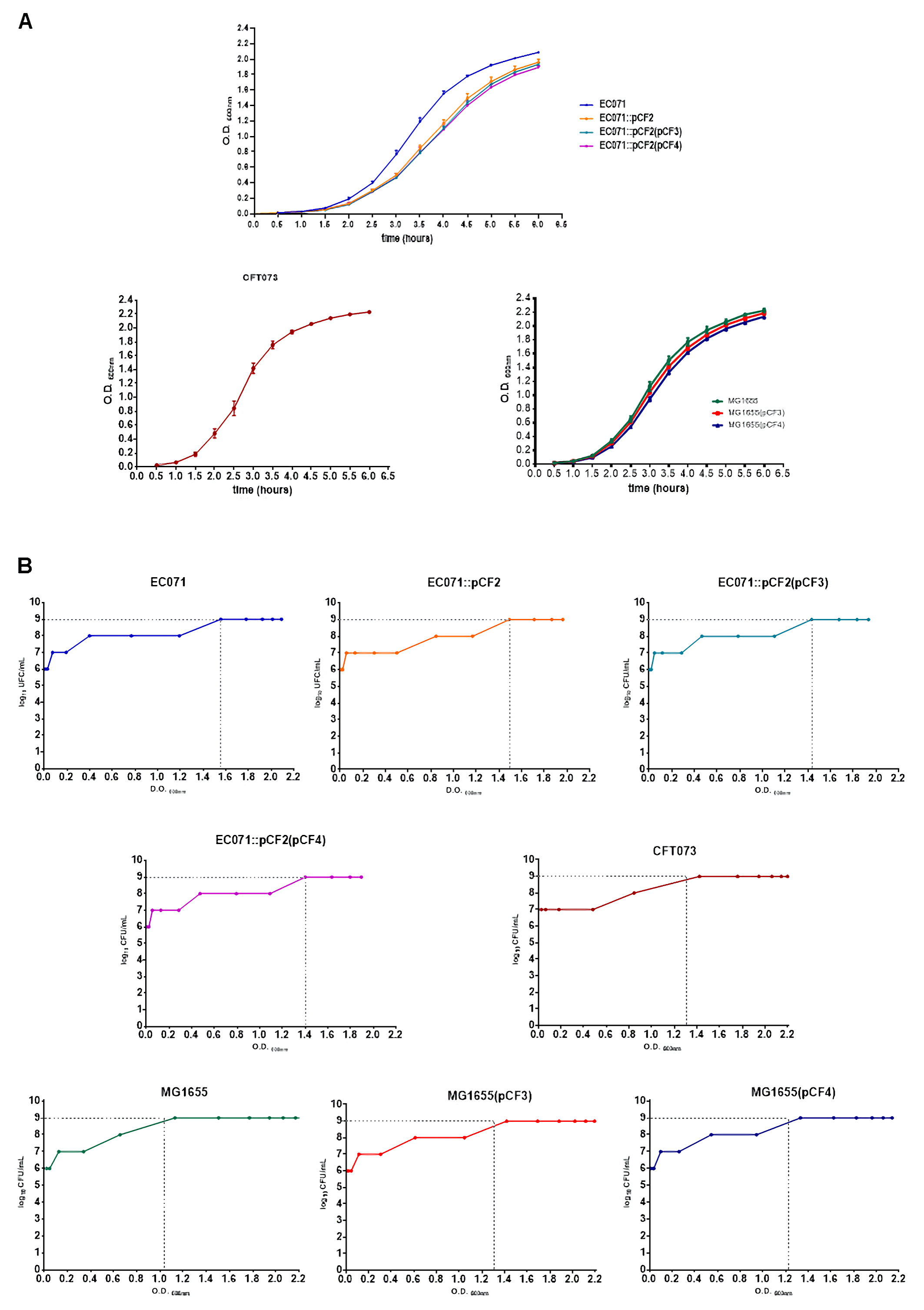

Supplement: Supplementary file 4 [file Image_2.tif]
