# Supplementary material for: Secreted Autotransporter Toxin (Sat) Mediates Innate Immune System Evasion
Source: Front Immunol. 2022 Feb 17;13:844878. doi: 10.3389/fimmu.2022.844878 (PMC8891578; doi:10.3389/fimmu.2022.844878)
Supplement: Supplementary file 5 [file Table_1.pdf]

**Supplementary table 1 –** Bacterial strains used in this study.

| Strains/plasmids | Characteristics | Reference |
|---|---|---|
| **Strains** | | |
| EC233/93 | Sat-producing EAEC used as positive control for Sat production detection | Vieira et al. (2020) |
| FBC114 | Sat-producing DAEC used as positive control for Sat production detection | Taddei et al. (2003) |
| *Shigella flexneri* M90T | SepA producing strain used as negative control for Sat production detection | Benjelloun-Touimi; Sansonetti; Parsot, (1995) |
| CFT073 | UPEC prototype strain used as positive control for murine sepsis model validation | Mobley et al. (1990) |
| DH5α | K-12 *E. coli* used as pCF3 and pCF4 host and as death control in serum resistance assays | Stratagene |
| MG1655 | K-12 *E. coli* used as pCF3 and pCF4 host and negative control for murine sepsis model validation | Jensen (1993) |
| S17λpir | Conjugative K-12 lysogenized for the *pir* gene, which permits replication of R6K plasmid replicons. Used as pCF2 host and donor strain in *sat* mutagenesis. | Simon; Proefer; Pühler (1983) |
| **Plasmids** | | |
| pGEM-T Easy | LacZ cloning vector ($Ap^r$) | Promega |
| pJP5603 | R6K suicide plasmid ($Kn^r$) | Penfold; Pemberton (1992) |
| pettac | pet22b+ derivative plasmid with tac promoter | Serre et al. (2008) |

**References**

Benjelloun-Touimi Z, Sansonetti PJ, Parsot C. SepA, the major extracellular protein of *Shigella flexneri*: autonomous secretion and involvement in tissue invasion. Mol Microbiol. 1995;17(1):123–135.

Jensen KF. The *Escherichia coli* K-12 “wild types” W3110 and MG1655 have an *rph* frameshift mutation that leads to pyrimidine starvation due to low *pyrE* expression levels. J Bacteriol. 1993;175(11):3401–3407.

Mobley HLT, Green DM, Trifillis AL, Johnson DE, Chippendale GR, et al. Pyelonephritogenic *Escherichia coli* and killing of cultured human renal proximal tubular epithelial cells: role of hemolysin in some strains. Infect Immun. 1990;58(5):1281–1289, 1990.

Penfold RJ, Pemberton JM. An improved suicide vector for construction of chromosomal insertion mutations in bacteria. Gene. 1992;118(1):145–146, 1992.

Serre K, Mohr E, Toellner KM, Cunningham AF, Granjeaud S, Bird R, et al. Molecular differences between the divergent responses of ovalbumin-specific CD4 T cells to alum-precipitated ovalbumin compared to ovalbumin expressed by *Salmonella*. Mol Immun. 2008;45(13):3558–3566.

Simon R, Proefer U, Pühler A. A broad host range mobilization system for in vivo genetic engineering: transposon mutagenesis in gram negative bacteria. Bio/Technology, 1983;1:784–791, 1983.

Taddei CR, Moreno ACR, Fernandes Filho A, Montemor LPG, Martinez MB. Prevalence of secreted autotransporter toxin gene among diffusely adhering *Escherichia coli* isolated from stools of children. FEMS Microbiol Lett. 2003;227:249–253.

Vieira PCG, Espinoza-Culupú AO, Nepomuceno R, Alves MR, Lebrun I, Elias WP, et al. Secreted autotransporter toxin (Sat) induces cell damage during enteroaggregative *Escherichia coli* infection. PLoS One. 2020;15(2):1–23.
