# Supplementary material for: Secreted Autotransporter Toxin (Sat) Mediates Innate Immune System Evasion
Source: Front Immunol. 2022 Feb 17;13:844878. doi: 10.3389/fimmu.2022.844878 (PMC8891578; doi:10.3389/fimmu.2022.844878)
Supplement: Supplementary file 6 [file Table_2.pdf]

Supplementary table 2 - Primers used for genetic constructions.

Primer	Sequence	Reference
sat	F: TCAGAAGCTCAGCGAATCATTG<br>R: CCATTATCACCAGTAAAACGCACC	Boisen et al. (2009)
M13	F: TGTAAAACGACGGCCAGT<br>R: CAGGAAACAGCTATGAC	This study
sat NdeI F	GATACGCATATGAATAAAATATACTCCCTTAA	This study
sat XhoI R	GTACTGCTCGAGTCAGAAAGAGTAACGGAAGTTGG	This study
NdeI pettac	CAGTCATGCTAGCCATATGTATATC	This study
XhoI pettac	GAACTGTATAAATGATAACTCGAGCACCAC	This study
sat2	F: GCTGGCTCTGGAGGAACAT<br>R: CCTTACCATTTCCGCTTGC	This study
pettac F	ATGGTGCATGCAAGGAGATG	This study
T7 terminator R	GCTAGTTATTGCTCAGCGG	This study
sat sdm	F: GAAATATTCGGAGACA**T**CGGC**G**CTGGAGCATACTTATATGATAAC<br>R: GTTATCATATAAGTATGCTCCAG**C**GCCG**A**TGTCTCCGAATATTTC	This study
Amp	F: CGAGTGGGTTACATCGAAC<br>R: CGCCTCCATCCAGTCTATTA	This study

Reference

Boisen N, Ruiz-Perez F, Scheutz F, Krogfelt KA, Nataro JP. Short report: high prevalence of serine protease autotransporter cytotoxins among strains of enteroaggregative *Escherichia coli*. Am J Trop Med Hyg. 2009;80(2):294–301.
