# Supplementary material for: Secreted Autotransporter Toxin (Sat) Mediates Innate Immune System Evasion
Source: Front Immunol. 2022 Feb 17;13:844878. doi: 10.3389/fimmu.2022.844878 (PMC8891578; doi:10.3389/fimmu.2022.844878)
Supplement: Supplementary file 7 [file Table_3.pdf]

Supplementary table 3 – EC071 genetic background analysis by whole genome sequencing.

Group	Gene	Reference Sequence (Genbank accesion number)	Reference Strain	EC071
SPATEs-encoding genes	*eatA*	AY163491	*E. coli* H10407	No similarity found
	epeA	AY258503.2	*E. coli* O113:H21 EH41, plasmid pO113	No similarity found
	espC	AF297061	*E. coli* E2348/69	No similarity found
	espI	AJ278144.1	*E. coli* O91:H- 4797/97	No similarity found
	espP	X97542.1	*E. coli* EDL933	No similarity found
	sigA	AF200692.2	*S. flexneri* 2a	No similarity found
	pic	NC_017626.1	*E. coli* 042	No similarity found
	sepA	CP037924.1	*S. flexneri* 5a cepa M90T, plasmid pWR100	No similarity found
	tsh	NC_009837.1	*E. coli* O1:K1:H7 APEC O1, plasmid pAPEC-O1-ColBM	No similarity found
	vat	AE014075.1	*E. coli* O6:K2:H1 CFT073	No similarity found
	pet	NC_017627.1	*E. coli* 042, plasmid pAA	No similarity found
	tagB	MH899681.1	*E. coli* QT598	No similarity found
	tagC			No similarity found
	sha	MH899684.1	*E. coli* strain QT598, plasmid Sha	No similarity found
Intrinsic virulence-related genes	*kpsM*	AE014075.1	*E. coli CFT073*	No similarity found
	kpsT			No similarity found
	iutA			No similarity found
	iucD			No similarity found
	focAICDFGH			No similarity found
	sfaADEFGSH			No similarity found
	papIBAHCDJKEFG			+
	afaABCDE	FM955458.1	*E. coli FV9863*	+
	draABCDE	AF329316.1	*E. coli IH11128*	+

Supplementary table 3 – cont.

Group	Gene	Reference Sequence (Genbank accesion number)	Reference Strain	EC071
Group 2-capsule	*kpsF-II*	NC_017631.1	*E. coli* ABU 83972	+
Group 2-capsule	*kpsE-II*	NC_017631.1	*E. coli* ABU 83972	+
Group 2-capsule	*kpsD-II*	NC_017631.1	*E. coli* ABU 83972	+
Group 2-capsule	*kpsU-II*	NC_017631.1	*E. coli* ABU 83972	+
Group 2-capsule	*kpsC-II*	NC_017631.1	*E. coli* ABU 83972	+
Group 2-capsule	*kpsS-II*	NC_017631.1	*E. coli* ABU 83972	+
Group 2-capsule	*kpsM-II-K5*	NC_017631.1	*E. coli* ABU 83972	No similarity found
Group 2-capsule	*kpsT-II-K5*	NC_017631.1	*E. coli* ABU 83972	No similarity found
Group 2-capsule	*kpsM-K2*	AE014075.1	*E. coli* CFT073 (O6:H1)	No similarity found
Group 2-capsule	*kpsT-K2*	AE014075.1	*E. coli* CFT073 (O6:H1)	No similarity found
Group 2-capsule	*neuS*	NC_007946.1	*E. coli* UTI89 (O18:H7)	No similarity found
Group 2-capsule	*neuE*	NC_007946.1	*E. coli* UTI89 (O18:H7)	No similarity found
Group 2-capsule	*neuC*	NC_007946.1	*E. coli* UTI89 (O18:H7)	No similarity found
Group 2-capsule	*neuA*	NC_007946.1	*E. coli* UTI89 (O18:H7)	No similarity found
Group 2-capsule	*neuB*	NC_007946.1	*E. coli* UTI89 (O18:H7)	No similarity found
Group 2-capsule	*neuD*	NC_007946.1	*E. coli* UTI89 (O18:H7)	No similarity found
Group 2-capsule	*kpsT-K1*	NC_007946.1	*E. coli* UTI89 (O18:H7)	No similarity found
Group 2-capsule	*kpsM-K1*	NC_007946.1	*E. coli* UTI89 (O18:H7)	No similarity found
K-15-capsule	*kpsC'-K5*	NC_008253.1	*E. coli* 536 (O6:H31)	+
K-15-capsule	*kpsS-K10*	NC_008253.1	*E. coli* 536 (O6:H31)	+
K-15-capsule	*kpsC-K10*	NC_008253.1	*E. coli* 536 (O6:H31)	No similarity found
K-15-capsule	*kpsT-K15*	NC_008253.1	*E. coli* 536 (O6:H31)	No similarity found
K-15-capsule	*kpsM-K15*	NC_008253.1	*E. coli* 536 (O6:H31)	No similarity found
Group 3-capsule	*kpsD-III*	NZ_JDRV01000043.1	*E. coli* MS499	No similarity found
Group 3-capsule	*kpsM-III*	NZ_JDRV01000043.1	*E. coli* MS499	No similarity found
Group 3-capsule	*kpsT-III*	NZ_JDRV01000043.1	*E. coli* MS499	No similarity found
Group 3-capsule	*kpsE-III*	NZ_JDRV01000043.1	*E. coli* MS499	No similarity found
Group 3-capsule	*kpsC-III*	NZ_JDRV01000043.1	*E. coli* MS499	No similarity found
Group 3-capsule	*kpsS-III*	NZ_JDRV01000043.1	*E. coli* MS499	+
Group 4-capsule	*gfcA*	NZ_CP009166.1	*E. coli* 1303 (O70:H32)	No similarity found
Group 4-capsule	*gfcB*	NZ_CP009166.1	*E. coli* 1303 (O70:H32)	No similarity found
Group 4-capsule	*gfcC*	NZ_CP009166.1	*E. coli* 1303 (O70:H32)	No similarity found
Group 4-capsule	*gfcD*	NZ_CP009166.1	*E. coli* 1303 (O70:H32)	No similarity found
Group 4-capsule	*gfcE*	NZ_CP009166.1	*E. coli* 1303 (O70:H32)	No similarity found
Group 4-capsule	*etp*	NZ_CP009166.1	*E. coli* 1303 (O70:H32)	No similarity found
Group 4-capsule	*etk*	NZ_CP009166.1	*E. coli* 1303 (O70:H32)	No similarity found

Supplementary table 3 - cont.

Group	Gene	Reference Sequence (Genbank accesion number)	Reference Strain	EC071
**Outer membrane proteins**	*ompP*	X74278.1	*E. coli* K-12 MG1655	No similarity found
	ompX (ompP)	U00096.3		+
	ompTc	NC_008563.1	*E. coli* APEC O1(O1:H7)	+
	ompTp	NC_007675.1	*E. coli* APEC A2363, plasmid pAPEC-O2-ColV	+
**Proteases**	*stcE*	CP000244.1	*E. coli* EDL933 (O157:H7)	No similarity found
	prc	NC_000913.3	*E. coli* K-12 MG1655	+
	nlpI	CU928161.2	*E. coli* S88 (O45:H7)	+
**Protectins**	*bor/ iss*	NC_009837.1	*E. coli* APEC O1 (O1:H7), plasmid pAPEC-O1-ColBM	No similarity found
	traT	J01769.1	*E. coli* plasmid R100	No similarity found
