# Supplementary material for: Secreted Autotransporter Toxin (Sat) Mediates Innate Immune System Evasion
Source: Front Immunol. 2022 Feb 17;13:844878. doi: 10.3389/fimmu.2022.844878 (PMC8891578; doi:10.3389/fimmu.2022.844878)
Supplement: Supplementary file 8 [file Table_4.pdf]

Supplementary table 4 - EC071 whole genome sequencing metrics [a].

Parameters	
Size (pb)	5 271 699
Contigs	262
Genes	5139
N50 [b]	86 427
N75 [c]	42 340
L50 [d]	19
L75 [e]	40
G+C (%)	50,53
Coverage	141x

[a] Calculated using the QUAST plataform (Gurevich et al., 2013).

[b] Base pair (bp) size that contigs with this or bigger size include half the total of bases.

[c] Base pair (bp) size that contigs with this or bigger size include ¾ of the total of bases.

[d] Number of contigs that are bigger or have the same N50 size.

[e] Number of contigs that are bigger or have the same N75 size.

Reference

Gurevich A, Saveliev V, Vyahhi N, Tesler G. QUAST: Quality assessment tool for genome assemblies. Bioinformatics, 2013;29:1072-1075.
